# Impact of Bidentate Pyridyl-Mesoionic Carbene Ligands: Structural, (Spectro)Electrochemical, Photophysical, and Theoretical Investigations on Ruthenium(II) Complexes

**DOI:** 10.1021/acsorginorgau.3c00005

**Published:** 2023-05-03

**Authors:** Tobias Bens, Jasmin A. Kübler, Robert R. M. Walter, Julia Beerhues, Oliver S. Wenger, Biprajit Sarkar

**Affiliations:** † Institut für Anorganische Chemie, Universität Stuttgart, Pfaffenwaldring 55, D-70569 Stuttgart, Germany; ‡ Institut für Chemie und Biochemie, Freie Universität Berlin, Fabeckstraße 34-36, 14195 Berlin, Germany; § Department of Chemistry, University of Basel, 4056 Basel, Switzerland

**Keywords:** mesoionic carbenes, (spectro)electrochemistry, photochemistry, ruthenium, bipyridine, metal to ligand charge transfer

## Abstract

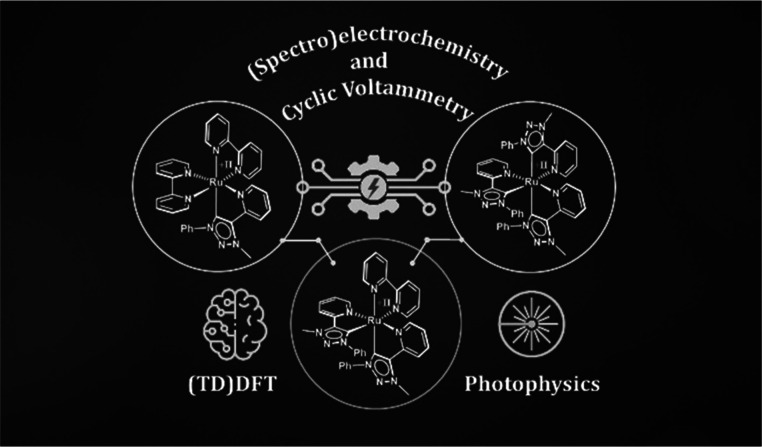

We present here new synthetic strategies for the isolation of a series of Ru(II) complexes with pyridyl-mesoionic carbene ligands (MIC) of the 1,2,3-triazole-5-ylidene type, in which the bpy ligands (bpy = 2,2′-bipyridine) of the archetypical [Ru(bpy)_3_]^2+^ have been successively replaced by one, two, or three pyridyl-MIC ligands. Three new complexes have been isolated and investigated via NMR spectroscopy and single-crystal X-ray diffraction analysis. The incorporation of one MIC unit shifts the potential of the metal-centered oxidation about 160 mV to more cathodic potential in cyclic voltammetry, demonstrating the extraordinary σ-donor ability of the pyridyl-MIC ligand, while the π-acceptor capacities are dominated by the bpy ligand, as indicated by electron paramagnetic resonance spectroelectrochemistry (EPR-SEC). The replacement of all bpy ligands by the pyridyl-MIC ligand results in an anoidic shift of the ligand-centered reduction by 390 mV compared to the well-established [Ru(bpy)_3_]^2+^ complex. In addition, UV/vis/NIR-SEC in combination with theoretical calculations provided detailed insights into the electronic structures of the respective redox states, taking into account the total number of pyridyl-MIC ligands incorporated in the Ru(II) complexes. The luminescence quantum yield and lifetimes were determined by time-resolved absorption and emission spectroscopy. An estimation of the excited state redox potentials conclusively showed that the pyridyl-MIC ligand can tune the photoredox activity of the isolated complexes to stronger photoreductants. These observations can provide new strategies for the design of photocatalysts and photosensitizers based on MICs.

## Introduction

**Figure 1:**
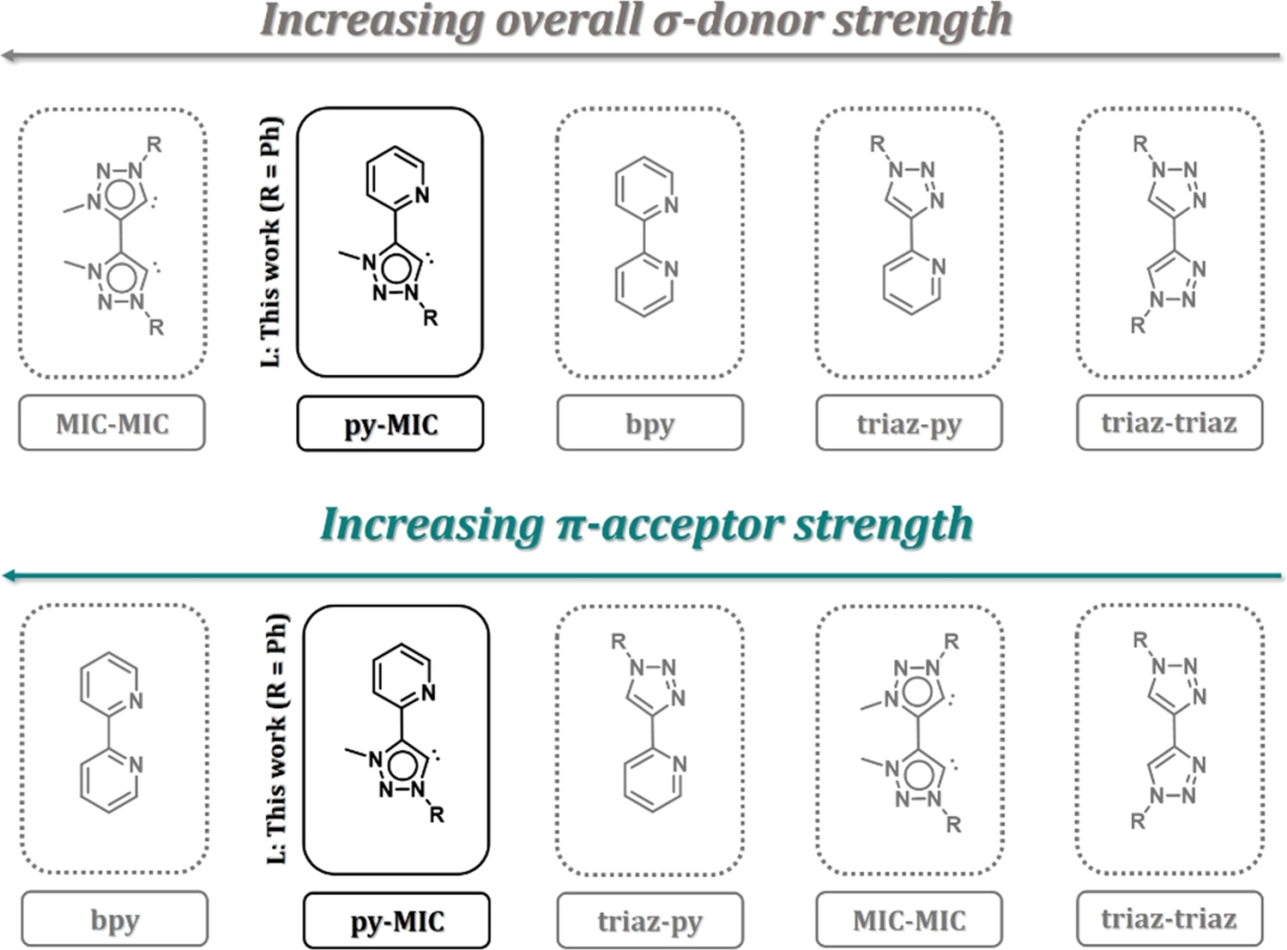
Comparison of the overall σ-donor properties and π-acceptor abilities in click-derived triazole and triazolylidene *fac*-[ReCl(CO)_3_] complexes by Suntrup et al.^9^ including the reported photoredox investigations in Fe(II)/(III) or Ru(II) complexes with the respective ligands.^10,13,16,17,35−37,39,40,42−45,47^

In recent years, mesoionic carbenes (MICs) of the 1,2,3-triazole-5-ylidene type have attracted increasing attention due to the versatility in ligand design and tunability of electronic properties. In comparison to their classical NHC counterparts, MICs feature stronger σ-donating properties and higher π-acceptor abilities leading to extensive utilization in both transition metal and main group chemistry.^1−8^ The incorporation of an additional donor substituent to the existing ligand backbone enables a bridge between the famous 2,2′-bipyridine (bpy, Figure 1) and newly designed tailor-made bidentate MIC-based ligands for transition metal complexes.

Recently, some of us investigated the overall σ-donor- and π-acceptor properties of chelating click-derived triazole and triazolylidene ligands in rhenium(I) complexes, combining cyclic voltammetry and IR spectroscopy (Figure 1).^9^ The study revealed a comparatively weaker σ-donor and π-acceptor ability of the bis-triazole ligand (triaz-triaz) in comparison to bpy, while the bis-MIC ligand (MIC-MIC) exhibits moderate π-acceptor and strong σ-donor strength. Including a single pyridyl moiety (triaz-py and py-MIC) in such ligands already drastically increases their π-acceptor capacities. In the case of the pyridyl-MIC (R = 2,6-diisopropylphenyl), the outstanding tunability of this class of ligands can be revealed. The ligand shows strong overall σ-donor and high π-acceptor properties, making it a suitable candidate for electrocatalytic and photocatalytic applications.

The growing interest in MICs is particularly evident in the photo-^10−23^ and redox-active chemistry,^9,24−31^ providing access to various applications in homogeneous catalysis^2−4,7,32^ and dyes for dye-sensitized solar cells.^33,34^ The modular synthesis and tunability of the electronic structure of the redox- and photoactive metal centers enable new generations of photoredox catalysts that have different properties compared to the well-established [Ru(bpy)_3_]^2+^.^32,35−47^

The lifetime of the photoactive ^3^MLCT state in the aforementioned complexes is primarily influenced by the ligand field splitting imparted by the coordination environment and high symmetry. Excellent reviews on the conceptual design of the ligand framework to increase the ligand field splitting have been published by several different working groups, showing the significant contributions in the past decades.^7,15,48−50^ Therefore, it is not surprising that the easily modulable bidentate triazoles and triazolylidene ligands have been investigated with various Ru(II) complexes to enhance their photophysical and photoredox properties.

Elliot and Skoglund first reported on triazole-containing ligands (triaz-triaz and triaz-py) and showed that excitation of the corresponding Ru(II) complexes leads to a destabilization of the ^3^MLCT state and a rapid depopulation via the nonradiatively deactivating ^3^MC state. Therefore, to generate luminescent metal complexes, the excitation of unoccupied triazole-based orbitals is not desirable due to their luminescence quenching character.^10,37,39,46^

In 2013, Albrecht and co-workers investigated the pyridyl-MIC ligand (R = Me) in the complex [Ru(bpy)_2_(py-MIC)]^2+^.^40^ They showed that the strong electron-donating nature of the MIC-based ligand resulted in the destabilization of the metal-centered highest occupied molecular orbital (HOMO), as indicated by the shift of the first metal-centered oxidation to more cathodic potential in cyclic voltammetry. According to the first ligand-centered reduction, the π-acceptor property of the pyridyl-MIC ligand leads to a minor destabilization of the lowest unoccupied molecular orbital (LUMO) orbital compared to the well-established [Ru(bpy)_3_]^2+^ complex.

Moreover, Wärnmark and Sundström in successive reports revealed the unique photophysical properties of MIC-containing transition metal complexes.^13,16,44^ The homoleptic tris-(MIC–MIC) Fe(III)^13^ complex was shown to be stabilized by the extreme electron-donating nature of the bidentate MIC ligands, resulting in an outstanding lifetime of 100 ps at room temperature, originating from an unusual LMCT.

In addition, a record-breaking MLCT lifetime of 528 ps was observed for the corresponding Fe(II) complex,^16^ which was only to be surpassed by the scorpion-type tris-NHC Fe(III) complex published in 2019 by Wärnmark and co-workers. The octahedral complex with two mono-anionic ligands showed a remarkable LMCT lifetime of 2.0 ns at room temperature.^51^

**Figure 2:**
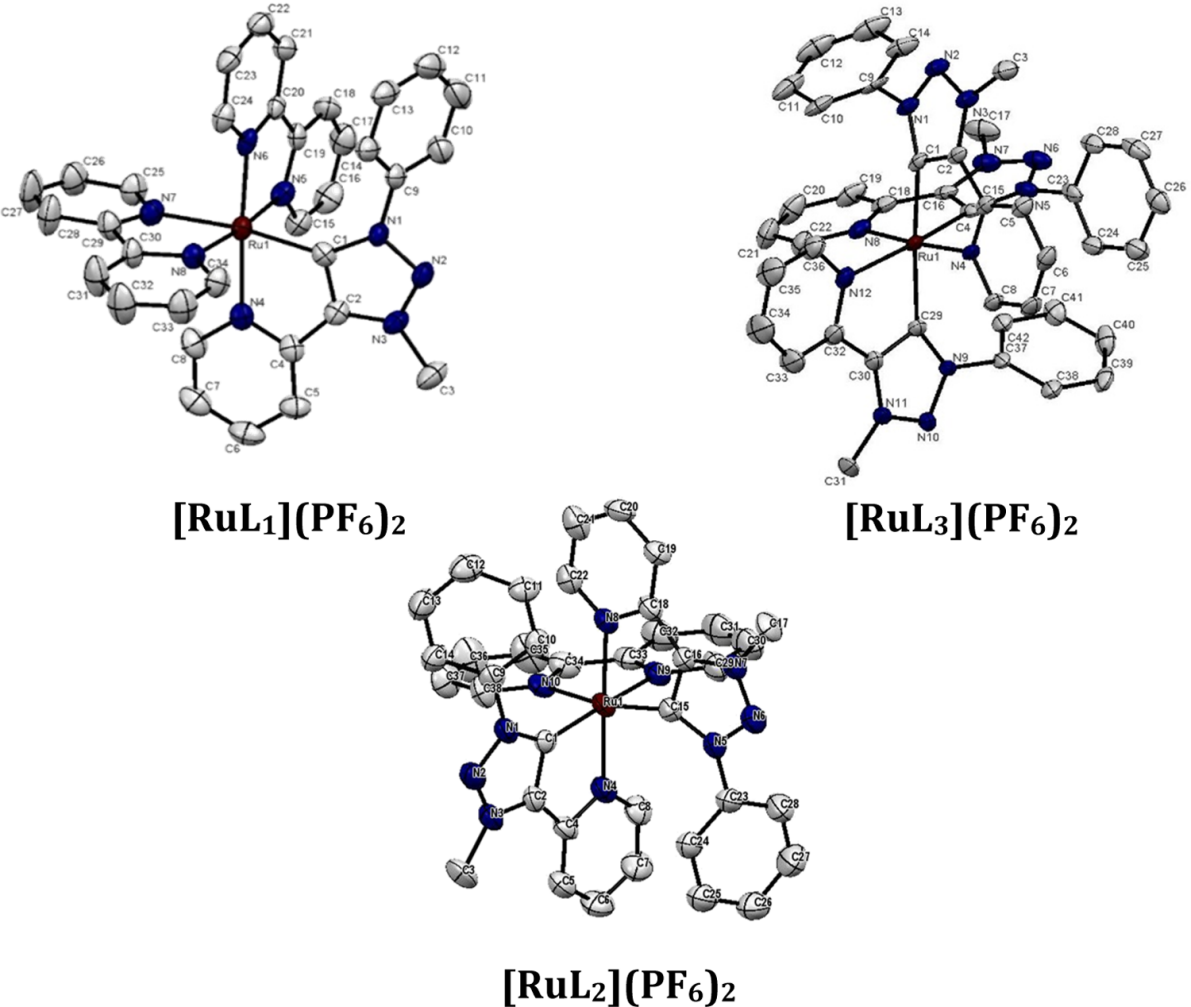
ORTEP representation of top left: **[RuL**_**1**_**](PF**_**6**_**)**_**2**_, bottom center: **[RuL**_**2**_**](PF**_**6**_**)**_**2**_, and top right: **[RuL**_**3**_**](PF**_**6**_**)**_**2**_ (hydrogen atoms and counter ions are omitted for clarity). Ellipsoids are drawn with 50% probability.

**Scheme 1:**
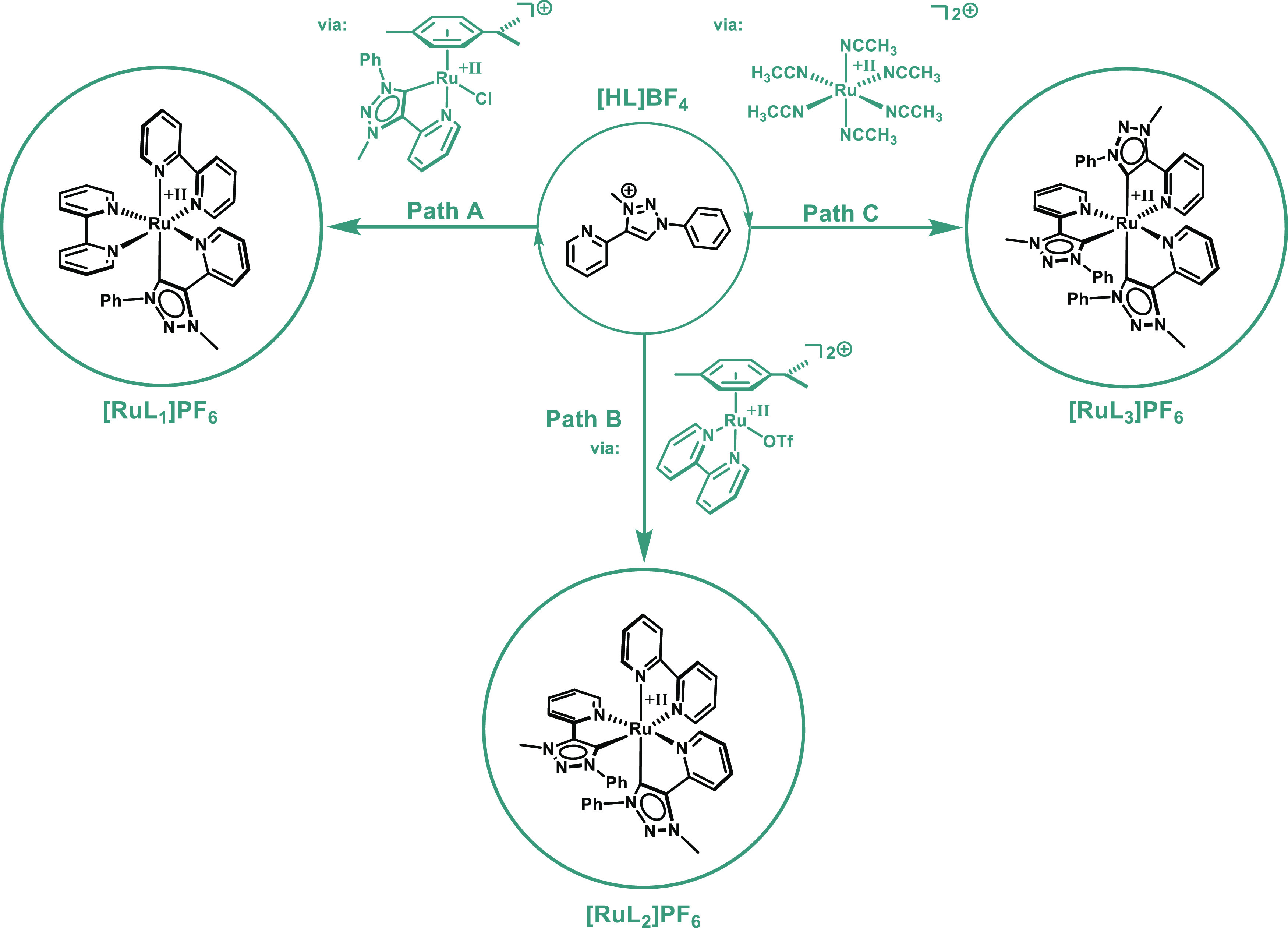
Synthetic Strategies for **[RuL**_**1**_**]**^**2+**^, **[RuL**_**2**_**]**^**2+**^, and **[RuL**_**3**_**]**^**2+**^ Path A: **[HL]BF_4_**, Ag_2_O, CH_3_CN, rt, 4 days; [Ru(*p*-cymene)Cl_2_]_2_, rt, 2 h (74%);^52^ bpy, AgPF_6_, ethylene glycol, 150 °C, 12 h; aq KPF_6_ (74%).^17^ Path B: [Ru(*p*-cymene)Cl_2_]_2_, bpy, MeOH, 2 h, rt; aq NH_4_PF_6_, 1 h (72%);^55^ HOTF, DCM, rt, 12 h (83%);^56^**[HL]BF_4_**, ethylene glycol, 180 °C, 12 h; aq KPF_6_ (49%, crude after workup); 2 weeks under *hv* in acetone/Et_2_O (16%).^57^ Path C: modified: RuCl_3_·3H_2_O, Zn(act), CH_3_CN, reflux, 2 days; AgBF_4_, CH_3_CN, reflux, 12 h (81%);^58^ modified: **[HL]BF_4_**, K_2_CO_3_, ethylene glycol, 160 °C, 16 h; aq NH_4_PF_6_ (46%).^57^

The immense influence of MICs on the photoredox properties of transition metal complexes has motivated us to conduct systematical studies on [Ru(L)*_n_*(bpy)*_m_*]^2+^ (*n* = 1–3, *m* = 0–2) complexes containing the pyridyl-MIC ligand **L**. Complexes **[RuL**_**1**_**]**^**2+**^, **[RuL**_**2**_**]**^**2+**^, and **[RuL**_**3**_**]**^**2+**^ were isolated and characterized by spectroscopic and crystallographic methods (Scheme 1 and Figure 2). Additionally, cyclic voltammetry, density functional theory (DFT), UV/Vis/NIR, and electron paramagnetic resonance–(EPR) spectroelectrochemistry (SEC) were performed to investigate the redox stability in different redox states as a function of the number of MIC units bound to the Ru(II) center. Time-resolved absorption and emission spectroscopic studies were performed to explore the influence of the MIC moiety on the electronic structure, with the aim to assess their potential application as photocatalysts in the future.

## Results and Discussion

### Synthesis and Characterization

The synthesis of **[HL]BF**_**4**_ as ligand precursor was performed according to a modified literature procedure.^52^ The reductive cleavage of the *N*-oxide at the pyridyl-triazole, after methylation with Meerwein’s salt, was realized using activated zinc in refluxing methanol overnight, instead of the expensive and toxic [Mo(CO)_6_], yielding nearly quantitative yields of 91% (see Supporting Information, Scheme S1).^53^

In order to obtain the complexes **[RuL**_**1**_**]**^**2+**^, **[RuL**_**2**_**]**^**2**+^, and **[RuL**_**3**_**]**^**2+**^, different synthetic strategies were used (Path **A**, Path **B**, and Path **C**, Scheme 1). **[RuL**_**1**_**]**^**2+**^ was synthesized based on a previously reported route.^17,54^ In the first step, the ligand **L** is generated in situ and transferred to form the respective [(**L**)Ru(*p*-cymene)Cl](PF_6_) complex via the well-established silver(I)-transmetalation route. After purification by column chromatography on alumina, the 1,2,3-triazolylidene half-sandwich complex was further reacted with bpy in the presence of AgPF_6_ in ethylene glycol at 150 °C.

Aqueous workup with KPF_6_, followed by column chromatography on alumina gave **[RuL**_**1**_**]**^**2+**^ in 74% yield (see Supporting Information, Section S2.30). These results are in good agreement with the previously described yields for 1,2,3-triazolylidene-based Ru(II) bpy complexes.^17,40,54^

In contrast, synthetic access to **[RuL**_**2**_**]**^**2+**^ and **[RuL**_**3**_**]**^**2+**^ has proven to be challenging. The isolation of **[RuL**_**2**_**]**^**2+**^, following the well-established synthetic approaches for similar reported NHC-based Fe(II) and Ru(II) complexes, resulted in a poor selectivity and purity of the crude product, making the isolation of **[RuL**_**2**_**]**^**2+**^ difficult.^59−66^ Therefore, we changed our strategy accordingly to Path **B**.^55−57^

The resulting crude product (see Supporting Information, Section S2.40) points to the formation of at least two regioisomers as indicated by ^1^H NMR and ^13^C{H} NMR spectroscopy, elemental analysis, and ESI-ToF-MS. Two well-separated methyl groups of two chemical inequivalent **L** at 4.52 and 4.40 ppm are observed in the ^1^H NMR spectra, while the ^13^C{H} NMR spectra show two signals of the methyl groups at 39.98 and 39.50 ppm besides two MIC-carbene signals at 188.29 and 186.62 ppm.

Upon recrystallization in acetone/Et_2_O by slow diffusion at room temperature, the main product decomposes in the presence of light, indicated by a drastic color change from orange to dark brown. After one month single crystals suitable for X-ray diffraction were isolated from the crude product mixture (16%, see Supporting Information, Section S2.40). Complete characterization of the isolated product indicated the isolation of the minor product of higher symmetry. The two methyl groups of **L** show a total integral of 6 H at 4.42 ppm and one methyl group at 39.65 ppm in the ^13^C{H} NMR next to the MIC-carbene signal at 185.09 ppm. The total number of 17 carbon signals underlines the higher symmetry of isolated **[RuL**_**2**_**]**^**2+**^. Unfortunately, ^1^H NMR spectroscopy of the remaining crude product solution clearly shows the decomposition of the main product during crystallization. Any attempt to identify the nature of the decomposition product remained unsuccessful.

Direct synthesis of **[RuL**_**3**_**]**^**2**^, starting from RuCl_3_·*x*H_2_O as described by Son et al.,^57^ resulted in a crude reaction mixture. We were not able to isolate the pure product due to the decomposition of **[RuL**_**3**_**]**^**2+**^ during column chromatography (SiO_2_ and basic/neutral aluminum oxide). Therefore, we focused our attention on the right choice of precursor to achieve good product selectivity combined with an easy workup of the crude product.

As a precursor, we chose the homoleptic [Ru(MeCN)_6_]^2+^ complex (see Supporting Information, Section S2.50). Starting from RuCl_3_·*x*H_2_O, activated zinc in acetonitrile was added as a reducing agent. The reaction mixture was refluxed for 2 days. After filtration, AgBF_4_ was added to abstract the chloride ligand in the remaining [RuCl_2_(MeCN)_4_], yielding 81% of [Ru(MeCN)_6_](BF_4_)_2_.^58^

With the precursor in hand, we started reinvestigating the literature procedure of Son et al.^57^ and observed a higher product selectivity for **[RuL**_**3**_**]**^**2+**^. The addition of K_2_CO_3_ led to an even higher selectivity and allowed milder reaction temperatures of 160 °C. Direct crystallization (slow diffusion/vapor diffusion) of the crude product in common solvents at room temperature, close to 0 °C, or lower temperatures (up to −20 °C) failed.

To avoid precipitation, the crude product was dissolved in acetone and cooled with liquid nitrogen until the mixture solidified. Et_2_O was added and cooled with liquid nitrogen until it solidified, too. The capped flask was transferred to a freezer to avoid decomposition in the presence of light and stored for 1 month at −20 °C resulting in dark orange crystals (46%) suitable for single X-ray diffraction analysis.

In the molecular structure of the crystal, all complexes **[RuL**_**1**_**]**^**2+**^, **[RuL**_**2**_**]**^**2+**^, and **[RuL**_**3**_**]**^**2+**^ display a distorted octahedral geometry (Figure 2). All bond angles along the axis in the Ru(II) center range between 169 and 177°, while the C–Ru–N and N–Ru–N angles are between 77 and 79°. The Ru-bond lengths are in the range of 2.05–2.16 Å, slightly longer than the respective Ru–C bond lengths (1.99–2.06 Å). Interestingly, the M–C bonds in [**RuL**_**3**_**]**^**2+**^, which are trans to each other show longer bond distances (2.06 Å) compared to the M–C (1.99 Å) bond trans to the M–N bond (2.17 Å). A plausible explanation is the increased trans-influence caused by the strong σ-donating properties of the MIC-carbenes. The bond length and angles of the chelating ligands are within the expected range.^9,17,19,24,67,68^

### (Spectro)Electrochemistry and (TD)DFT Calculations

#### Cyclic Voltammetry

To investigate the influence of the total number of pyridyl-MICs in the complexes **[RuL**_**1**_**]**^**2+**^, **[RuL**_**2**_**]**^**2+**^, and **[RuL**_**3**_**]**^**2+**^, cyclic voltammetry, UV/Vis/NIR-, EPR-SEC, and (TD)DFT calculations were performed. The electrochemical investigations provide further evidence for the HOMO/LUMO gap in the presented pyridyl-MIC-containing Ru(II) complexes.

**Figure 3:**
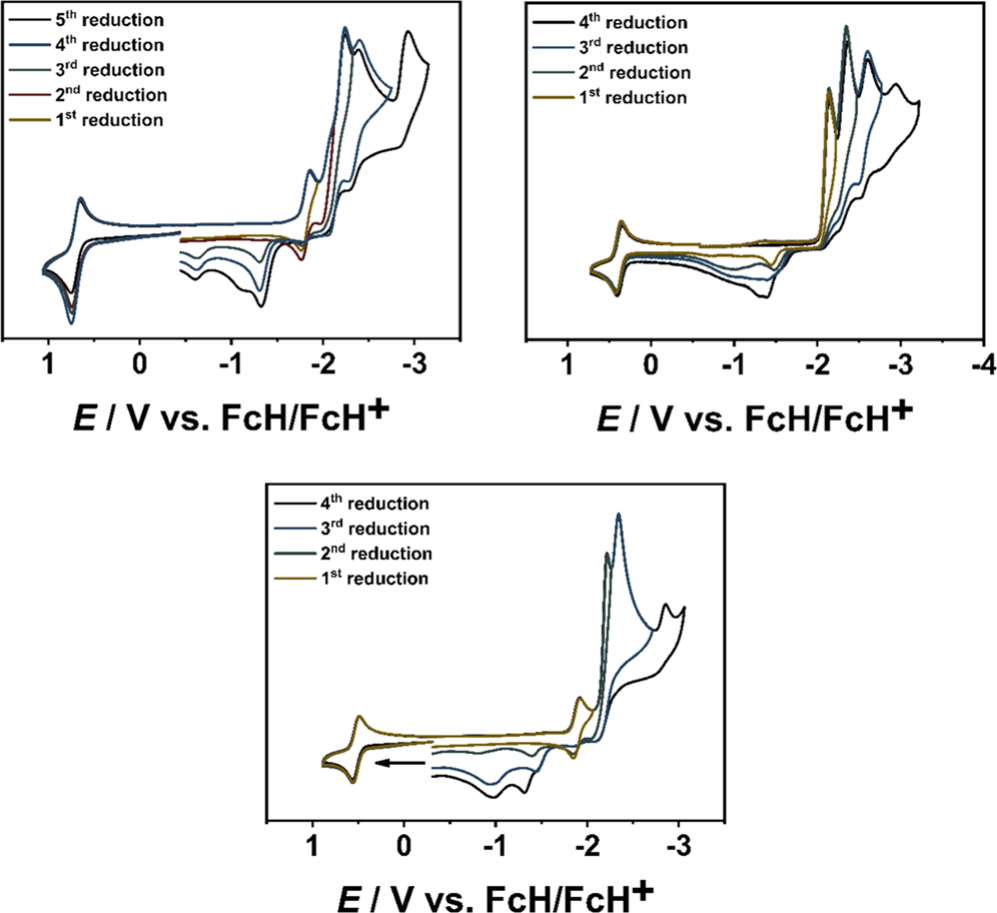
Cyclic voltammograms of **[RuL**_**1**_**]**^**2+**^ (top left), **[RuL**_**1**_**]**^**2+**^ (bottom center), and **[RuL**_**3**_**]**^**2+**^ (top right) in CH_3_CN and 0.1 M Bu_4_NPF_6_ with a scan rate of 100 mV/s.

All complexes exhibit redox-rich cyclic voltammograms with one reversible oxidation and multiple reductions (Figures 3 and S13–S15).

**Figure 4:**
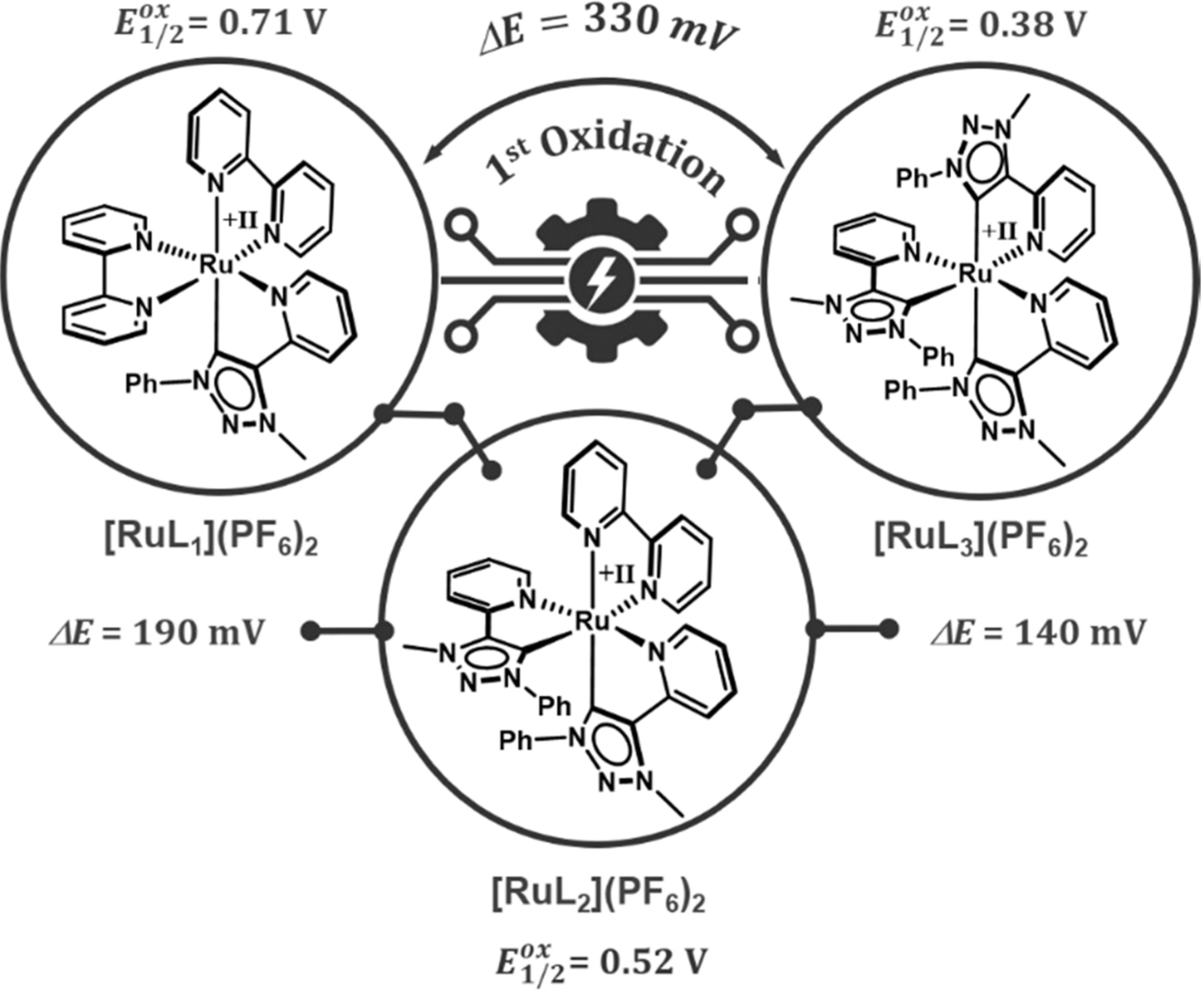
Effect of incorporating MIC moieties on the oxidation potential in **[RuL**_**1**_**]**^**2+**^, **[RuL**_**2**_**]**^**2+**^, and **[RuL**_**3**_**]**^**2+**^.

In the reported complexes, oxidation can be assigned to a predominantly metal-centered Ru(II)/Ru(III) redox couple (see Figure 7, EPR-SEC). The incorporation of one or more MIC units shifts the oxidation potential to more cathodic potential. Taking the archetypical complex **[Ru(bpy)**_**3**_**]**^**2+**^ into account, a shift between 140 and 190 mV per MIC unit can be estimated for the oxidation (Figures 3, 4, and Table 1).^17,69,70^ This is in good agreement with previously reported oxidation potentials for MIC-containing Ru(II) complexes.^17^ Considering earlier reports by Albrecht and co-workers, the replacement of the phenyl substituent by an electron-donating methyl substituent at the N^1^ position further destabilizes the metal-centered HOMO, as indicated by the decreased oxidation potential.^40^

**Table 1:** Redox Potentials of **[RuL**_**1**_**]**^**2+**^, **[RuL**_**2**_**]**^**2+**^, **[RuL**_**3**_**]**^**2+**^, and **[Ru(bpy)**_**3**_**]**^**2+**^(69,70) in CH_3_CN and 0.1 M NBu_4_PF_6_ at 100 mV/s vs FcH/FcH^+^

	*E*_1/2_^ox^ (Δ*E*) (V)	*E*_1/2_^red1^(Δ*E*) (V)	*E*_p_^red2^ (V)	*E*_p_^red3^ (V)	*E*_p_^red4^ (V)	*E*_p_^red5^ (V)
**[RuL_1_]^2+^**	0.71(0.11)	–1.81(0.09)	–2.02	–2.11	–2.34	–2.89
**[RuL_2_]^2+^**	0.52(0.10)	–1.89(0.08)	–2.22	–2.34	–2.86	
**[RuL_3_]^2+^**	0.38(0.08)	–2.14^a^	–2.31	–2.56	–2.85	
**[Ru(bpy)_3_]^2+^**	0.89	–1.73	–1.92	–2.16		

a *E*_1/2_^red1^ = *E*_p_^red1^.

The first reduction is primarily determined by the π-acceptor properties of the ligand. Since bpy is a better π-acceptor than the pyridyl-MIC ligand **L**, the first reduction processes in **[RuL**_**1**_**]**^**2+**^ and **[RuL**_**2**_**]**^**2+**^ are bpy-based (Figure 7, EPR-SEC), resulting in a reversible first reduction around −1.85 V. Similar observations have been made for bpy-containing Ru(II)MIC complexes.^17,40^ The influence of **L** on the reduction potential is particularly evident in the complex **[RuL**_**3**_**]**^**2+**^, containing only **L**. The reduction potentials display a cathodic shift from **[Ru(bpy)**_**3**_**]**^**2+**^ to **[RuL**_**1**_**]**^**2+**^ and **[RuL**_**2**_**]**^**2+**^ by about 100 mV. Going from **[RuL**_**2**_**]**^**2+**^ to **[RuL**_**3**_**]**^**2+**^, a shift of 250 mV in the reduction potential is observed, indicating a strong change in the π-acceptor properties of the complex. However, **[RuL**_**3**_**]**^**2+**^ displays only irreversible reduction processes. The electron-rich nature of **[RuL**_**3**_**]**^**2+**^ could lead to dissociation of the ligand arm upon reduction, as described for analogous electron-rich Ru(II) terpyridine complexes.^71^

#### UV/Vis/NIR- and EPR-SEC

To gain a detailed insight into the redox stability and the nature of the metal-centered oxidation, as well as the ligand-centered reduction, EPR-, and UV/Vis/NIR-SEC were performed on **[RuL**_**1**_**]**^**2+**^, **[RuL**_**2**_**]**^**2+**^, and **[RuL**_**3**_**]**^**2+**^.

In the native form, all three complexes exhibit MLCT bands in the region of 300–550 nm as described for similar systems (Figure 4).^17,42,61,69^ Time-dependent density functional theory (TD-DFT) calculations (see Supporting Information, Sections S7.10–S7.30) assign the electronic transitions from a metal d-orbital to a uniformly distributed ligand-centered charge transfer (d(Ru) → π*(L)) to all ligands.

**Figure 5:**
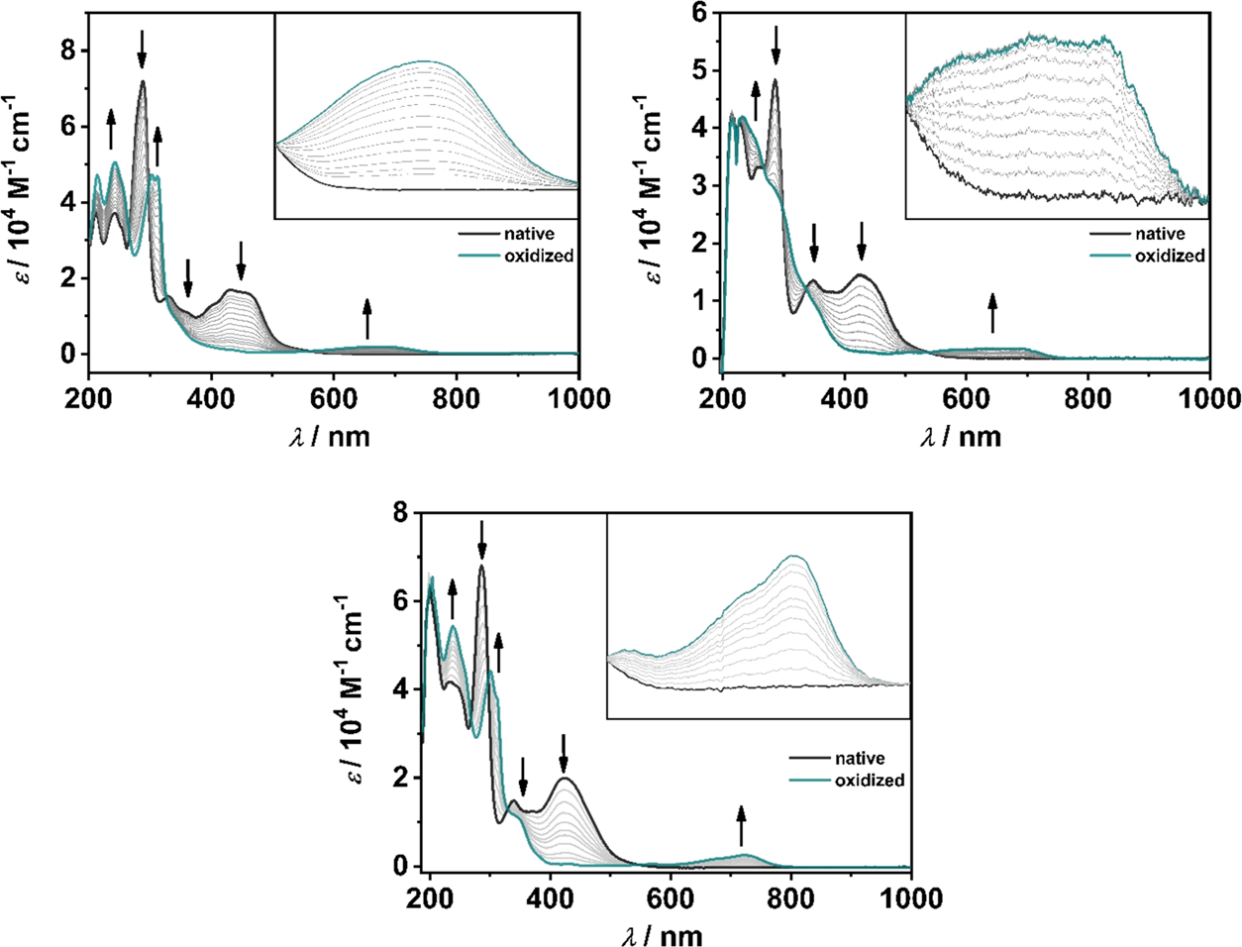
Changes in the UV/vis/NIR spectra of **[RuL**_**1**_**]**^**2+**^ (top left, inset: 560–810 nm), **[RuL**_**2**_**]**^**2+**^ (bottom center, inset: 550–830 nm), and **[RuL**_**3**_**]**^**2+**^ (top right, inset: 535–780 nm) in CH_3_CN/0.1 M Bu_4_NPF_6_ during the first oxidation with a Au working electrode.

**Figure 6:**
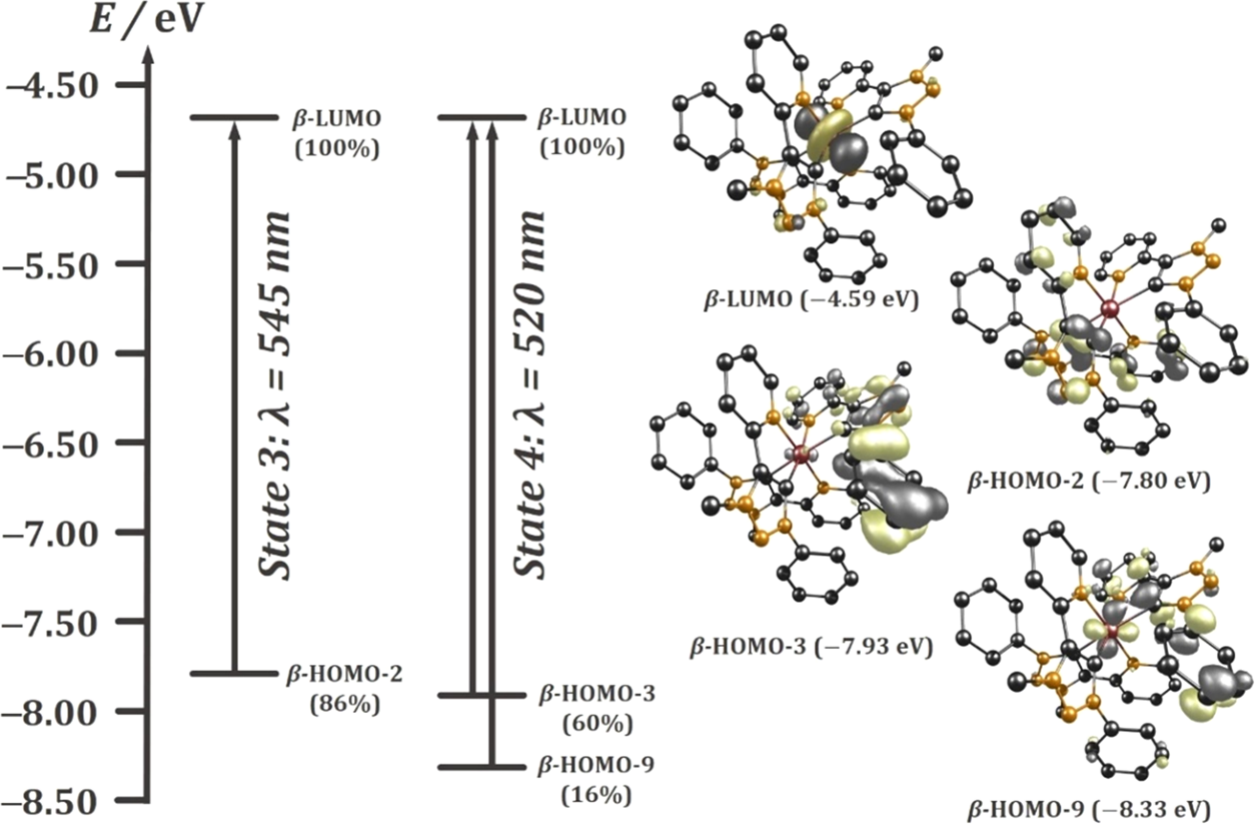
Involved molecular orbitals in LMCT of complex **[RuL**_**3**_**]**^**3**+^ calculated by TD-DFT (PBE0/RIJCOSX/D3(BJ)/def2-TZVP/CPCM, iso value = 0.052).

Upon oxidation, all MLCT bands diminish and new bands appear in the 535–830 nm range (Figure 5). In the case of **[RuL**_**1**_**]**^**3+**^, the bands can be assigned to a metal–ligand to metal (MLMCT) charge transfer (see Supporting Information, Section S7.10). However, the picture changes drastically when moving from **[RuL**_**1**_**]**^**3+**^ to **[RuL**_**2**_**]**^**3+**^ and **[RuL**_**3**_**]**^**3+**^. The metal contribution significantly decreases, shifting the mixed MLMCT to a dominant ligand to metal (LMCT) charge transfer in the case of **[RuL**_**3**_**]**^**3+**^ (Figure 6, see Supporting Information, Section S7.30).

Similar observations were made by Kalyanasundaram and co-workers.^72^ The incorporation of strong σ-donating ligands with good π-accepting ligands in Ru(III) complexes leads to a larger mixing of the metal d(π)-orbitals with the π*-orbitals of the π-accepting ligand. The resulting HOMO-orbitals display a stronger mixed-metal–ligand character. In contrast, electron-rich ligands with weak π-acceptor properties result in predominantly ligand-centered HOMO-orbitals of the homoleptic Ru(III).

Interestingly, the contribution in the LMCT is not only limited to the pyridyl-MIC framework but also to the phenyl substituent on the triazolylidene moiety, revealing the strong impact of the substituents on the stability and photoredox chemistry in **[RuL**_**2**_**]**^**3+**^ and **[RuL**_**3**_**]**^**3+**^.^13,44^ Notably, the reduction of the oxidized **[RuL**_**1**_**]**^**3+**^, **[RuL**_**2**_**]**^**3+**^, and **[RuL**_**3**_**]**^**3+**^ restores the starting spectra indicating a completely reversible oxidation (see Supporting Information, Figures S23–S25) further underlining the redox stability caused by the pyridyl-MIC ligand and its phenyl substituent.

**Figure 7:**
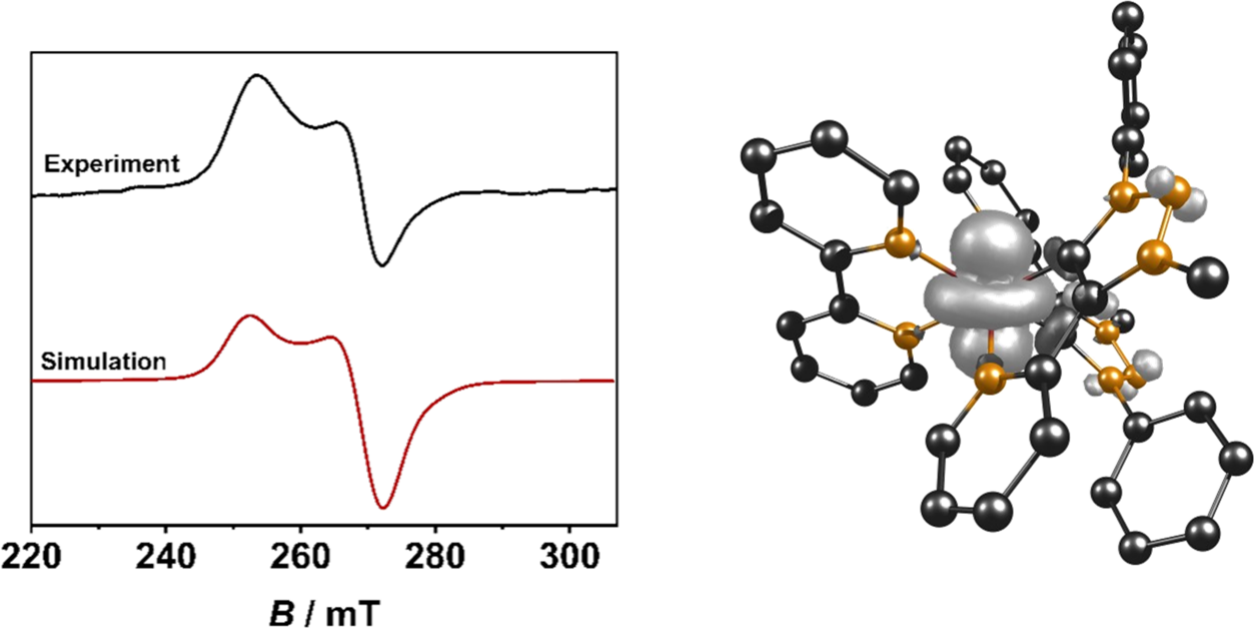
EPR spectrum of **[RuL**_**2**_**]**^**3+**^ at −175 °C in CH_3_CN/0.1 M Bu_4_NPF_6_ with a Pt working electrode (left, *g*_⊥_ = 2.50 and *g*_||_ = 2.69) and spin density plot (right, iso value = 0.003, PBE0/RIJCOSX/D3(BJ)/def2-TZVP/CPCM).

The oxidized species **[RuL**_**1**_**]**^**3+**^ and **[RuL**_**3**_**]**^**3+**^ generated through EPR-SEC with a platinum working electrode in CH_3_CN/0.1 M Bu_4_NPF_6_ at −175 °C shows an unresolved axial signal at *g* = 2.60 for **[RuL**_**1**_**]**^**2+**^ and *g* = 2.55 for **[RuL**_**3**_**]**^**2+**^ (see Supporting Information, Figures S17 and S21), while **[RuL**_**2**_**]**^**2+**^ displays a well-resolved axial signal at *g*_⊥_ = 2.50 and *g*_||_ = 2.69 (Figure 7). The observation of *g*_||_ > *g*_⊥_ is rather unusual for octahedrally coordinated Ru(III) species where the trend is usually the other way around.^73^

The high *g*-anisotropy of Δ*g* = 0.19 indicates a predominantly metal-centered oxidation, while spin density calculations further confirm the metal-centered spin of 90%, for all three complexes. Only a small ligand contribution can be assigned based on spin density calculations, as shown by the spin density plots (Figure 7, see Supporting Information, Figures S18 and S22).^17,74,75^

**Figure 8:**
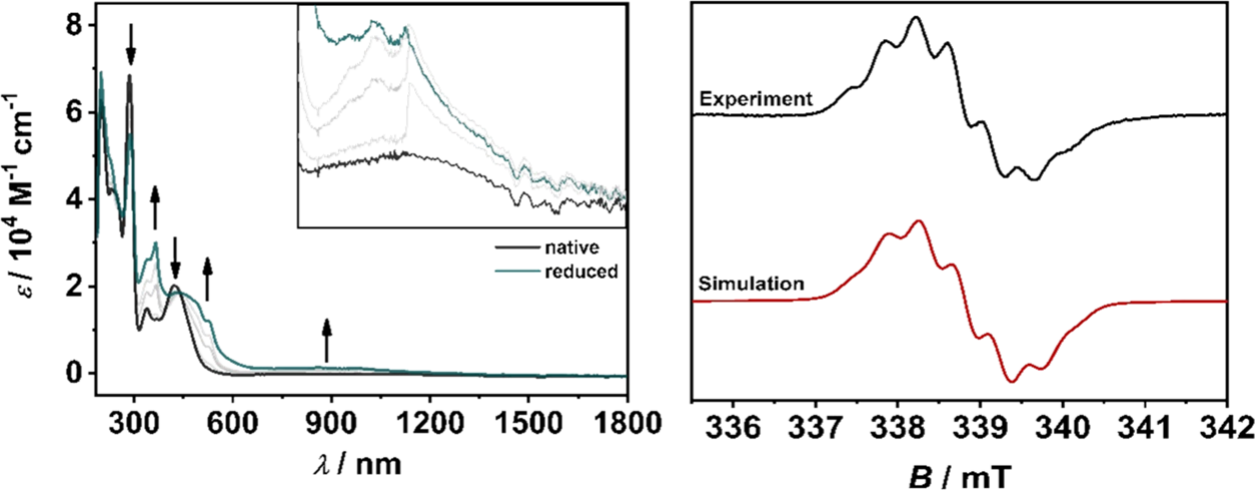
Changes in the UV/Vis/NIR spectra of **[RuL**_**2**_**]**^**2+**^ (left) in CH_3_CN/0.1 M Bu_4_NPF_6_ during first reduction (inset: 580–1800 nm) with a Au working electrode. EPR spectrum of **[RuL**_**2**_**]**^**1+**^ (right, *g* = 1.998) at room temperature in CH_3_CN/0.1 M Bu_4_NPF_6_ with a Pt working electrode.

In the case of **[RuL**_**1**_**]**^**2+**^ and **[RuL**_**2**_**]**^**2+**^, the first reduction already appears to be reversible in the cyclic voltammetry experiments (Figures 3 and 8; see Supporting Information, Figure S23). The reversibility of the first reduction in **[RuL**_**1**_**]**^**2+**^ could be further verified by UV/Vis/NIR-SEC (see Supporting Information, Figure S23), while in the case of **[RuL**_**2**_**]**^**2+**^, a partial degradation is observed after bulk electrolysis (see Supporting Information, Figure S24). It is plausible that the electron-rich nature of the two pyridyl-MIC ligands in **[RuL**_**2**_**]**^**2+**^ results in ligand dissociation upon reduction leading to partial degradation of the complex.

Both complexes show broad bands in the range from 550 to 2000 nm in the one-electron reduced form. TD-DFT calculations assign the broad bands to numerous ligand-based intra- and interligand charge transfer bands (ILCT) with a minor metal contribution (see Supporting Information, Sections S7.10 and S7.20). The MLLCT bands (450–600 nm) are red-shifted due to the increased electron density in **[RuL**_**1**_**]**^**1+**^ and **[RuL**_**2**_**]**^**1+**^ compared to **[RuL**_**1**_**]**^**2+**^ and **[RuL**_**2**_**]**^**2+**^ (Figure 8).

EPR-SEC and spin density calculations of the reduced **[RuL**_**1**_**]**^**2+**^ and **[RuL**_**2**_**]**^**2+**^ complexes further confirm the dominant ligand-centered reduction (Figure 8, see Supporting Information, Figure S18). The isotropic EPR signal at *g* = 1.996 for **[RuL**_**1**_**]**^**1+**^ and *g* = 1.998 for **[RuL**_**2**_**]**^**1+**^ shows a typical bpy^•–^/Ru(II) situation.^75−78^ In the case of **[RuL**_**2**_**]**^**1+**^, hyperfine coupling to the nitrogen and the four hydrogens of the bpy ligand was observed (Figure 8). A plausible explanation for the unresolved hyperfine coupling in **[RuL**_**1**_**]**^**1+**^ can be found in the total number of bpy ligands in **[RuL**_**1**_**]**^**1+**^ and the electron-rich nature of the pyridyl-MIC ligand. The electron-rich pyridyl-MIC ligand forces the electron into the bpy ligand of **[RuL**_**2**_**]**^**1+**^, while in **[RuL**_**1**_**]**^**1+**^, two bpy ligands can serve as electron reservoirs, resulting in a poorly localized electronic spin and higher dynamics.

#### Photophysics

**Figure 9:**
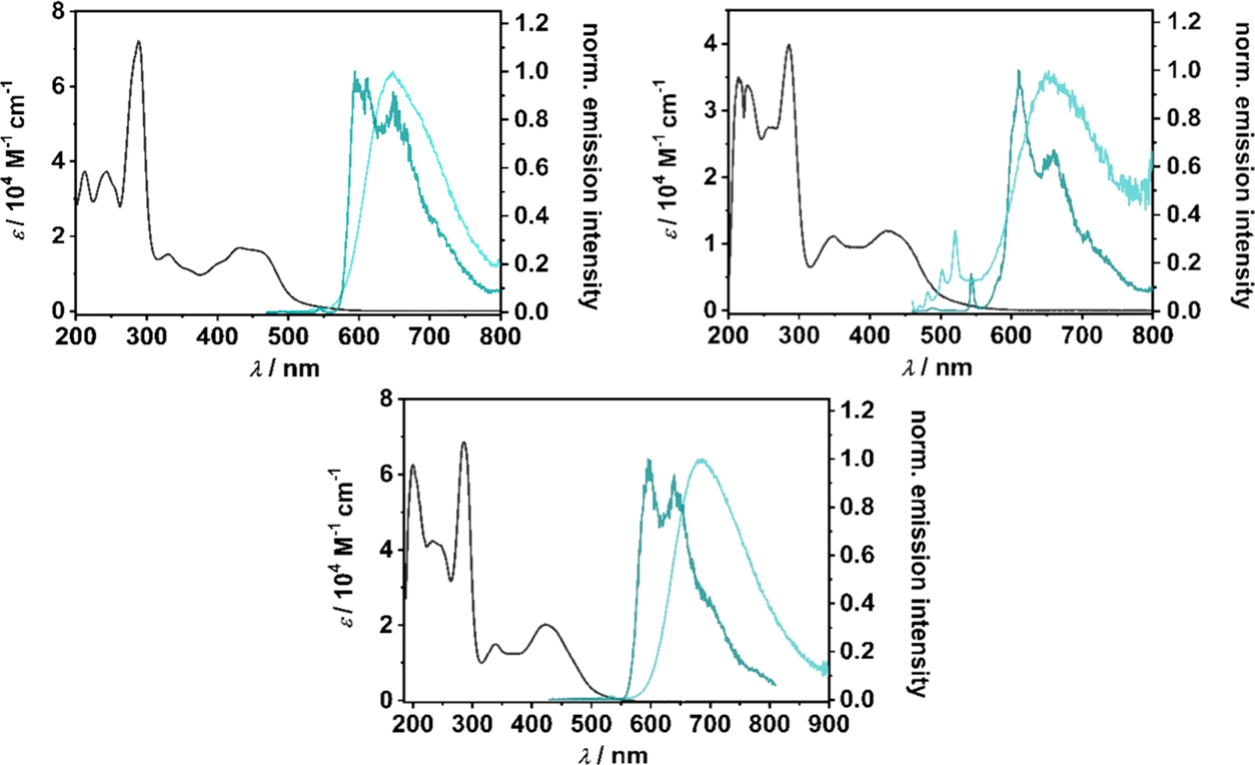
UV/Vis absorption (black) at 293 K and normalized emission spectra at 293 K (light blue line) and 77 K (dark blue line) of **[RuL**_**1**_**]**^**2+**^ (top left, excited at 460 nm), **[RuL**_**2**_**]**^**2+**^ (bottom center, excited at rt: 460 nm and 77 K: 420 nm), and **[RuL**_**3**_**]**^**2+**^ (top right, excited at 450 nm).

Time-resolved absorption and emission spectroscopy are powerful techniques to further explore the electronic structure of the pyridyl-MIC Ru(II) complexes. Emission studies in MeCN at room temperature and 77 K were performed to investigate the effects of the total number of MICs incorporated into the presented polypyridine Ru(II) complexes (Table 2 and Figure 9).

**Table 2:** Spectroscopic Data of **[RuL**_**1**_**]**^**2+**^, **[RuL**_**2**_**]**^**2+**^, **[RuL**_**3**_**]**^**2+**^, and **[Ru(bpy)**_**3**_**]**^**2+**^ in MeCN

	λ_em_ (nm)	τ_abs_ (ns)	Φ_em_ (%)	*E*_00_ (eV)	τ_em_ (ns)
**[RuL_1_]^2+^**	594, 649	71^a^	0.88^e^	2.21	62^f^
**[RuL_2_]^2+^**	597, 640	99^b^, 103^c^	1.05^e^	2.07	99^g^
**[RuL_3_]^2+^**	610, 653	26, 344^d^^,^^i^	0.64^e^	2.07	28, 288^h^^,^^i^
**[Ru(bpy)_3_]^2+^**	611^80^	890^80^	5.9^80^	2.10^69^	890^80^

a At λ = 368 nm (λ_ex_ = 460 nm).

b λ = 370 nm (λ_ex_ = 430 nm).

c λ = 420 nm (λ_ex_ = 430 nm).

d at λ = 420 nm (λ_ex_ = 435 nm).

e Average out of 5 measurements.

f At λ = 620 nm (λ_ex_ = 460 nm).

g At λ = 660 nm (λ_ex_ = 370 nm).

h At λ = 644 nm (λ_ex_ = 435 nm).

i The biexponential decay behavior is tentatively attributed to a photodegradation process (Figure S68).

**[RuL**_**1**_**]**^**2+**^, **[RuL**_**2**_**]**^**2+**^, and **[RuL**_**3**_**]**^**2+**^ show emission spectra with typical vibrational structures for polypyridine Ru(II) complexes in the 600–900 nm range.^79^ Such systems typically show a fast intersystem crossing (*k*_isc_) from the ^1^MLCT state to the ^3^MLCT state upon excitation. The thermal population (*E*_a_) of the ^3^MC state from the ^1/3^MLCT nesting states results in a nonradiative decay (*k*_nr_). MICs as strong σ-donor ligands can destabilize the metal-centered ^3^MC state, while the π-acceptor properties of the pyridyl moiety lead to the stabilization of the ^1/3^MLCT state. Consequently, the energy barrier for *E*_a_ increases, disfavoring quenching of the ^3^MLCT state.^48−50^

However, the incorporation of pyridyl-MIC ligands leads to a decrease in the lifetimes of the ^3^MLCT excited state (τ_abs_) in all three cases compared to the well-established **[Ru(bpy)**_**3**_**]**^**2+**^ (Table 2).^17,40,80^ The same trend is observed for the photoluminescence quantum yields.

After irradiation of **[RuL**_**3**_**]**^**2+**^, decomposition products emitting in the range of 400–550 nm are formed (Figure S64), featuring luminescence lifetimes in the order of 16–28 ns (Figure S67). This decomposition process is significantly less prevalent at lower temperatures. It seems plausible that the thermal population of the ^3^MC state plays a decisive role in the photo-decomposition of **[RuL**_**3**_**]**^**2+**^. The population of the e_g_^*^-orbitals can result in dissociation of the electron-rich pyridyl-MIC ligand, while steric bulk and an unsymmetrical geometry (Table S1) around the Ru(II) center can potentially further lower the ligand field strength, giving access to a fast population of the ^3^MC state.^48,49,71,81−83^

The MLCT excited state lifetimes of **[RuL**_**1**_**]**^**2+**^, **[RuL**_**2**_**]**^**2+**^, and **[RuL**_**3**_**]**^**2+**^ at 293 K are long enough to allow for photo-induced electron transfer reactions in photocatalysis, or for electron injection into semiconductors.^84,85^ Interestingly, comparison of the excited state ^3^MLCT energies (*E*_00_) of **[RuL**_**1**_**]**^**2+**^, **[RuL**_**2**_**]**^**2+**^, and **[RuL**_**3**_**]**^**2+**^ with **[Ru(bpy)**_**3**_**]**^**2+**^ shows that the incorporation of the pyridiyl-MIC ligand does not significantly change the relative energy of *E*_00_.

Combining the oxidation or reduction potentials of the ground states with the excited state ^3^MLCT energies (*E*_00_) allows us to estimate the potentials for the oxidative *E_red_ and reductive *E_ox_ quenching of the ^3^MLCT states to assess the application potential of the presented complexes as photocatalysts or photosensitizers, as illustrated in a Latimer diagram (Table 3).

**Table 3:**
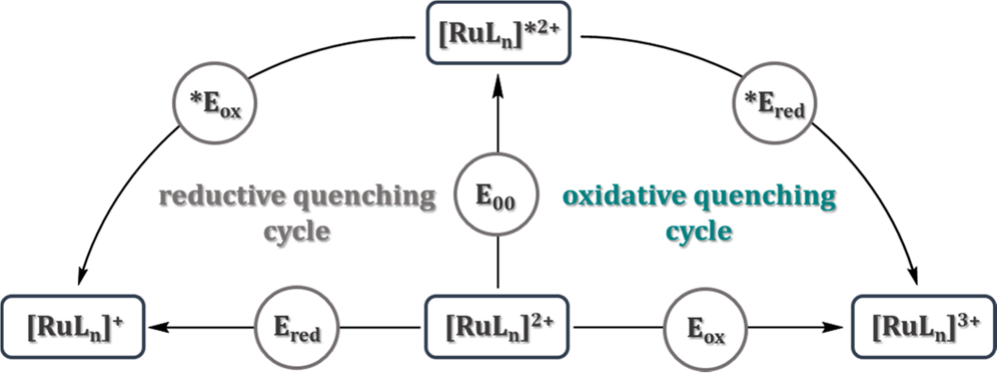
Latimer Diagram with Relevant Potentials for Photoexcitation^86^^,^^a^^,^^b^

	*E*_red_ (V)	*E*_ox_ (V)	*E_red_ (V)	*E_ox_ (V)	*E*_00_ (eV)
**[RuL_1_]^2+^**	–1.43	1.09	1.12	–0.78	2.21
**[RuL_2_]^2+^**	–1.51	0.90	1.17	–0.56	2.07
**[RuL_3_]^2+^**	–1.76	0.76	1.29	–0.29	2.05
**[Ru(bpy)_3_]^2+^**(69)	–1.34	1.27	0.83	–0.76	2.10

a The data in the table shows that *E_red_ follow the trend **[Ru(bpy)**_**3**_**]**^**2+**^ < **[RuL**_**1**_**]**^**2+**^ < **[RuL**_**2**_**]**^**2+**^ < **[RuL**_**3**_**]**^**2+**^. A similar observation was already described by Suntrup et al. exploring the influence of pyridyl-MICs and bi-MICs on Ru(II) and Os(II) bipyridine complexes. Increasing the number of MICs within the transition metal complexes results in higher *E_red_,^17^ making them in particular attractive as photoreductant, while the oxidative quenching potential *E_ox_ follows the trend **[Ru(bpy)**_**3**_**]**^**2+**^ ≈ **[RuL**_**1**_**]**^**2+**^ > **[RuL**_**2**_**]**^**2+**^ > **[RuL**_**3**_**]**^**2+**^, demonstrating the strong electron-donating nature of the MIC ligands.

b All potentials with FcH/FcH^+^ were converted vs SCE = 0.38 V in MeCN/0.1 M NBu_4_PF_6_ at room temperature.

These results indicate that the redox potentials of the excited states of such ruthenium complexes can indeed be tuned over a broad range, making metal complexes containing MIC ligands interesting candidates for photocatalytic applications.

## Conclusions

In summary, a series of three new pyridyl-MIC-based Ru(II) complexes were synthesized and fully characterized by ^1^H and ^13^C NMR spectroscopy, mass spectrometry, elemental analysis, and single-crystal X-ray diffraction. Cyclic voltammetry reveals an interesting trend: the incorporation of an MIC moiety in the Ru(II) polypyridine complex shifts the metal-centered reversible oxidation by ∼160 mV to a more cathodic potential, while the first bpy-centered reduction shifts by ∼100 mV to a more negative potential after an additional MIC unit is incorporated. The replacement of all bpy ligands by the strongly electron-donating pyridyl-MIC ligand **L** shifts the reduction potential up by 250 mV. The changes in the electronic structures of the different redox states were further investigated using EPR- and UV/Vis/NIR-SEC and supported by (TD)DFT. Upon oxidation, the MLLCT character of **[RuL**_**1**_**]**^**3+**^ is reverted to a dominant LMCT going to **[RuL**_**3**_**]**^**3+**^. Our photophysical investigations showed that within the series from **[RuL**_**1**_**]**^**2+**^ to **[RuL**_**2**_**]**^**2+**^, and **[RuL**_**3**_**]**^**2+**^, the excited-state reduction potential *E_red_ increases and the excited-state oxidation potential *E_ox_ becomes less negative. The MLCT lifetimes are within the expected range and in combination with the electrochemical data, a new series of potential photocatalysts could be developed. The presented complexes are in particular interesting as photoreductants, while the incorporation of the strongly σ-donating pyridyl-MIC ligands decreases the photooxidative capability. Extrapolation of the total number of MICs incorporated in Ru(II) complexes could even lead to stable Ru(III) metal complexes opening up new opportunities for photosensitizers and photocatalytic applications.

## Experimental Section

### General Procedures, Materials, and Instrumentation

*Caution!*: Compounds containing azides are potentially explosive. Although we never experienced any problems during synthesis or analysis, all compounds should be synthesized only in small quantities and handled with great care!

Unless otherwise noted, all reactions were carried out using standard Schlenk line techniques under an inert atmosphere of argon (Linde Argon 4.8, purity 99.998%). All reactions which require heating were performed with an oil bath.

Commercially available chemicals were used without further purification. The solvents used for metal complex synthesis and catalysis were available from MBRAUN MB-SPS-800 solvent system and degassed by standard techniques prior to use. The identity and purity of compounds were established via ^1^H and ^13^C{H} NMR spectroscopy, elemental analysis, and mass spectrometry.

Solvents for cyclic voltammetry and UV/vis- and EPR-spectroelectrochemical measurements were dried and distilled under argon and degassed by common techniques prior to use. Column chromatography was performed over silica 60 M (0.04–0.063 mm).

^1^H and ^13^C{^1^H} NMR spectra were recorded on a Bruker Avance 500 spectrometer at 19–22 °C. Chemical shifts are reported in ppm referenced to the residual solvent peaks.^87^

The following abbreviations are used to represent the multiplicity of the signals: s (singlet), d (doublet), t (triplet), q (quartet), p (pentet), sept (septet).

Mass spectrometry was performed on an Agilent 6210 ESI-TOF.

Elemental analyses were performed with an Elementar Micro Cube elemental analyzer.

### X-ray Diffraction

X-ray data were collected on a BRUKER Smart AXS, BRUKER D8 Venture, or Bruker Kappa Apex2duo system. Data were collected at 100(2) or 140(2) K, respectively, using graphite-monochromatic Mo Kα radiation (λ_α_ = 0.71073 Å). The strategy for the data collection was evaluated by using the APEX2 or Smart software. The data were collected by standard “ω scan techniques” or “ω–φ scan techniques” and were scaled and reduced using APEX2, SAINT+, and SADABS software.

The structures were solved by direct methods using SHELXL-97 or intrinsic phasing using SHELXL-2014/7 and refined by full-matrix least squares with SHELXL-2014/7, refining on *F*^2^. Nonhydrogen atoms were refined anisotropically. If it is noted, bond length and angles were measured with Mercury, version 3.8.^88−95^

### Electrochemistry

Cyclic voltammograms were recorded with a Metrohm Autolab potentiostat (PGSTAT 204) with a conventional three-electrode configuration consisting of a glassy carbon working electrode, a platinum auxiliary electrode, and a coiled silver wire as a pseudo reference electrode. The (decamethyl)ferrocene/(decamethyl)ferrocenium couple was used as internal reference. All measurements were performed at room temperature with a scan rate between 25 and 1000 mV s^–1^. The experiments were carried out in absolute acetonitrile containing 0.1 M Bu_4_NPF_6_ (Sigma-Aldrich, ≥99.0%, electrochemical grade) as the supporting electrolyte.

### Spectroelectrochemistry

UV/vis spectra were recorded with a J&M TIDAS spectrometer. UV/vis-spectroelectrochemical measurements were carried out in an optically transparent thin-layer electrochemical (OTTLE)^96^ cell (CaF_2_ windows) with a gold-mesh working electrode, a platinum-mesh counter electrode, and a silver-foil pseudo reference. EPR spectra at the X-band frequency (ca. 9.5 GHz) were obtained with a Magnettech MS-5000 benchtop EPR spectrometer equipped with a rectangular TE 102 cavity and a TC HO4 temperature controller. The measurements were carried out in synthetic quartz glass tubes. For EPR spectroelectrochemistry, a three-electrode setup was employed using two Teflon-coated platinum wires (0.005 in. bare and 0.008 in. coated) as the working and counter electrodes and a Teflon-coated silver wire (0.005 in. bare and 0.007 in. coated) as the pseudo reference electrode. The low-temperature EPR spectra were recorded at −175 °C. The experiments were carried out in absolute acetonitrile or CH_2_Cl_2_ containing 0.1 M Bu_4_NPF_6_ as the supporting electrolyte. The same solvents as for the CV measurements were used for each compound.

### DFT

The program package ORCA 4.1. was used for all DFT calculations.^97^ Starting from the molecular structure obtained from X-ray diffraction geometry optimizations were carried out using the PBE0^98^ functional and no symmetry restrictions were imposed during the optimization. All calculations were performed with empirical van der Waals correction (D3).^99−102^ The restricted and unrestricted DFT methods were employed for closed- and open-shell molecules, respectively, unless stated otherwise. Convergence criteria were set to default for geometry optimization (OPT), and tight for SCF calculations (TIGHTSCF). Triple-ζ-valence basis sets (def2-TZVP)^103^ were employed for all atoms. Calculations were performed using resolution of the identity approximation^104−110^ with matching auxiliary basis sets^111,112^ for geometry optimizations and numerical frequency calculations and the RIJCOSX (combination of the resolution of the identity and chain of spheres algorithms) approximation for single-point calculations using the PBE0 functional.^98^ Low-lying excitation energies were calculated with time-dependent DFT (TD-DFT). Solvent effects were taken into account with the conductor-like polarizable continuum model, CPCM.^113^ Spin densities were calculated according to the Mulliken population analysis.^114^ The absence of imaginary frequency spin densities, molecular orbitals, and difference densities were visualized with the Chemcraft 1.8 program.^115^ All molecular orbitals are illustrated with an iso value of 0.052. All calculated TD-DFT spectra are Gaussian broadened with a bandwidth of 25 nm at half-height.

### Photophysical Measurements

Steady-state luminescence spectra at room temperature and 77 K were measured using a Fluorolog-3-22 instrument from Horiba Jobin-Yvon. Transient absorption and kinetic emission and absorption measurements were performed on an LP920-KS instrument from Edinburgh Instruments in MeCN. Excitation source was a pulsed Quantel Brilliant b ND:YAG laser equipped with a Rainbow optical parameter oscillator (OPO) with a pulse energy of 13 mJ at 435 nm and 20 mJ at 460 nm. The solutions typically had an optical density below 0.4 and were deaerated through three cycles of freeze–pump–thaw. The quantum yields were measured on a Hamamatsu absolute photoluminescence quantum yield spectrometer C11347 Quantaurus QY with a sample concentration of 2.5 × 10^–5^ M, and the solutions were deaerated by bubbling Ar for 10 min.

## Synthetic Procedures

### Synthetic Strategy for [HL]BF_4_

According to a modified procedure, the triazole-containing *N*-oxide [HL–O] (1.04 g, 3.21 mmol) was dissolved in 20 mL of CH_2_Cl_2_. Me_3_OBF_4_ (1.04 g, 7.03 mmol) was added, and the mixture was stirred at room temperature for 5 days. The solvent was evaporated and the residue was suspended in abs. MeOH (80 mL). Activated zinc (0.835 g, 12.84 mmol) was added and heated under reflux overnight. The reaction mixture was filtered through celite and purified by column chromatography CH_2_Cl_2_/MeOH (10:1) to obtain the product as an off-white solid (0.74 g, 2.92 mmol, 91%). ^1^H NMR (400 MHz, CD_3_CN) δ (ppm) = 9.23 (s, 1H), 8.89–8.82 (m, 1H), 8.10 (td, *J* = 7.8, 1.7 Hz, 1H), 8.03–7.89 (m, 3H), 7.80–7.72 (m, 3H), 7.68–7.62 (m, 1H), 4.66 (s, 3H); ^13^C {^1^H} NMR (101 MHz, CD_3_CN) δ (ppm) = 151.4, 143.9, 142.7, 139.4, 136.0, 133.2, 131.6, 128.3, 127.2, 125.7, 122.7, 42.1; Anal. calcd for C_14_H_13_BF_4_N_4_: C, 51.89, H, 4.04, N, 17.29; found: C, 51.99, H, 4.15, N, 17.29.^52,53^

### Synthetic Strategy for [RuL_1_](PF_6_)_2_

In a 15 mL Schlenk flask, the respective [Ru(L)(*p*-cymene)Cl]BF_4_ (50 mg, 0.084 mmol, 1 equiv), bipyridine (26 mg, 0.169 mmol, 2 equiv), and AgPF_6_ (43 mg, 0.169 mmol, 2 equiv) were dissolved in degassed ethylene glycol (4 mL). The reaction mixture was capped and heated to 150 °C for 12 h. After cooling to room temperature, the resulting dark orange mixture was treated with aqueous KPF_6_ and extracted with CH_2_Cl_2_ (3 × 40 mL). The organic phase was washed with water (5 × 50 mL) and dried over Na_2_SO_4_. Additional crystallization from slow diffusion of Et_2_O into a concentrated solution of [RuL_1_](PF_6_)_2_ dissolved in CH_2_Cl_2_ yielded in an orange crystalline solid of [RuL_1_](PF_6_)_2_ (57 mg, 0.061 mmol, 72%). In the case of an insufficient conversion, purification by column chromatography (aluminum oxide, activated with 5 wt % water; CH_2_Cl_2_/acetone 100:0 → 100:15) resulted in pure [RuL_1_](PF_6_)_2_.

Single crystals suitable for X-ray diffraction were obtained by slow vapor diffusion of Et_2_O into a concentrated solution of [RuL_1_](PF_6_)_2_ in acetone at 4 °C.

^1^H NMR (500 MHz, CD_3_CN) δ (ppm) = 8.45 (d, *J* = 8.2 Hz, 1H), 8.42 (d, *J* = 8.1 Hz, 1H), 8.35 (d, *J* = 5.6 Hz, 1H), 8.27 (d, *J* = 8.1 Hz, 1H), 8.16 (d, *J* = 8.1 Hz, 1H), 8.06–7.95 (m, 5H), 7.70–7.68 (m, 1H), 7.67 (d, *J* = 5.6 Hz, 1H), 7.61–7.56 (m, 2H), 7.43–7.36 (m, 3H), 7.27–7.21 (m, 2H), 7.20–7.16 (m, 1H), 7.08 (t, *J* = 8.0 Hz, 2H), 6.92 (dd, *J* = 8.4, 1.1 Hz, 2H), 6.82 (ddd, *J* = 7.3, 5.7, 1.3 Hz, 1H), 4.58 (s, 3H); ^13^C{^1^H} NMR (126 MHz, CD_3_CN) δ (ppm) = 185.6, 158.0, 157.6, 157.5, 156.5, 156.5, 153.9, 153.0, 152.5, 152.1, 150.5, 146.8, 139.2, 139.0, 138.5, 138.1, 137.7, 137.2, 131.2, 130.3, 128.6, 128.3, 128.0, 127.4, 126.0, 125.912, 125.1, 124.8, 124.4, 124.1, 122.6, 39.9; HRMS (ESI) *m*/*z*: [RuL_1_](PF_6_)^+^ calcd for [C_34_H_27_F_6_N_8_PRu]^+^ 795.1126; found 795.1120, [RuL_1_]^2+^ calcd for [C_34_H_28_N_8_Ru]^2+^ 325.0739; found 325.0731; Anal. calcd for C_34_H_28_F_12_N_8_P_2_Ru: C, 43.46, H, 3.00, N, 11.93; found: C, 43.60, H, 3.06, N, 11.60.

### Synthetic Strategy for [RuL_2_](PF_6_)_2_

In a 15 mL Schlenk flask, [Ru(bpy)(*p*-cymene)OTf]OTf (34 mg, 0.049 mmol, 1 equiv) and [HL]BF_4_ (30 mg, 0.093 mmol, 2 equiv) were dissolved in degassed ethylene glycol (4 mL). The reaction mixture was capped and heated to 180 °C for 12 h. After cooling to room temperature, the resulting dark orange mixture was treated with aqueous KPF_6_ and extracted with CH_2_Cl_2_ (3 × 40 mL). The organic phase was washed with water (5 × 50 mL) and dried over Na_2_SO_4_. The crude product was dry-loaded on Celite and purified by column chromatography (inversed column, interchim puriFlash XS 520 Plus, column: PF-15C18AQ-F0040; H_2_O/CH_3_CN 100:0 → 80:20). The crude product was extracted with CH_2_Cl_2_ (3 × 40 mL) and dried over Na_2_SO_4_. The solvent was removed under reduced pressure, and the remaining dark orange solid (23 mg, 0.022 mmol, 46% crude) was dissolved in (CH_3_)_2_CO (3 mL) and overlayed with Et_2_O under light. The dark orange solution turned dark brown after 2 days, and after 1 month, single crystals suitable for X-ray diffraction have been obtained.

The collected crystals were used as seed crystals to induce crystallization from the crude product in the follow-up reactions. The resulting red crystals (7.8 mg, 0.077 mmol, 16%) were used without further purification.

^1^H NMR (700 MHz, CD_3_CN) δ (ppm) = 8.42 (d, *J* = 7.0 Hz, 2H), 8.29 (d, *J* = 6.5 Hz, 2H), 8.00 (td, *J* = 8.1, 1.5 Hz, 2H), 7.58 (d, *J* = 8.0 Hz, 2H), 7.53 (td, *J* = 7.9, 1.4 Hz, 2H), 7.42 (ddd, *J* = 7.5, 5.5, 1.2 Hz, 2H), 7.28–7.23 (m, 4H), 7.11–7.08 (m, 4H), 7.05 (dd, *J* = 8.4, 1.2 Hz, 4H), 6.71 (ddd, *J* = 7.3, 5.7, 1.4 Hz, 2H), 4.42 (s, 6H); ^13^C{^1^H} NMR (176 MHz CD_3_CN) δ (ppm) = 185.1, 156.6, 154.4, 153.3, 151.6, 145.6, 139.1, 138.5, 137.0, 131.2, 130.0, 128.3, 125.9, 124.8, 124.8, 121.6, 39.6; HRMS (ESI) *m*/*z*: [RuL_2_](PF_6_)^+^ calcd for [C_38_H_32_F_6_N_10_PRu]^+^ 875.1491; found 875.1487, [RuL_2_]^2+^ calcd for [C_38_H_32_N_10_Ru]^2+^ 365.0922; found 365.0925; Anal. calcd for C_38_H_32_F_12_N_10_P_2_Ru: C, 44.76, H, 3.16, N, 13.74; found: C, 44.71, H, 3.20, N, 13.40.

### Synthetic Strategy for [RuL_3_](PF_6_)_2_

In a 15 mL Schlenk flask, [Ru(CH_3_CN)_6_](BF_4_)_2_ (54 mg, 0.103 mmol, 1 equiv), [HL]BF_4_ (100 mg, 0.310 mmol, 3 equiv), and K_2_CO_3_ (47 mg, 0.341 mmol, 3.3 equiv) were dissolved in degassed ethylene glycol (4 mL). The reaction mixture was capped and heated to 160 °C for 16 h. After cooling to room temperature, the resulting dark red mixture was treated with aqueous NH_4_PF_6_, whereas a dark orange precipitate was formed. The orange solid was filtered off and extensively washed with H_2_O, EtOAc, and Et_2_O. The remaining solid was dissolved in acetone (15 mL), cooled down with liquid nitrogen, overlayed with Et_2_O, and cooled down with liquid nitrogen, too. The capped flask was stored at −20 °C for 1 month inducing crystallization of dark orange crystals suitable for X-ray diffraction analysis. The remaining crystalline solid was vigorously washed with Et_2_O and decanted to remove the remaining brownish solid yielding a dark orange crystalline solid of [RuL_3_](PF_6_)_2_ (52 mg, 0.047 mmol, 46%).

^1^H NMR (500 MHz, CD_3_CN) δ (ppm) = 8.25 (d, *J* = 5.4 Hz, 1H), 8.13 (d, *J* = 8.1 Hz, 1H), 7.99 (td, *J* = 7.9, 1.5 Hz, 1H), 7.82 (d, *J* = 5.5 Hz, 1H), 7.60–7.47 (m, 5H), 7.46–7.39 (m, 3H), 7.34–7.23 (m, 2H), 7.19 (t, *J* = 7.0 Hz, 1H), 7.14–7.04 (m, 4H), 7.04–6.98 (m, 4H), 6.75 (dddd, *J* = 13.2, 7.3, 5.8, 1.8 Hz, 2H), 6.51 (dd, *J* = 8.4, 1.1 Hz, 2H), 4.55 (s, 3H), 4.43 (s, 3H),4.30 (s, 3H); ^13^C{^1^H} NMR (126 MHz, CD_3_CN) δ (ppm) = 191.1, 187.0, 186.6, 157.8, 156.0, 153.7, 151.9, 151.4, 150.9, 147.1, 146.3, 144.1, 140.0, 139.3, 139.0, 138.4, 136.6, 135.8, 131.4, 130.8, 130.6, 129.99, 129.96, 129.6, 126.5, 126.1, 125.5, 125.3, 124.7, 124.2, 122.2, 121.49, 121.1, 39.9, 39.6, 39.3; HRMS (ESI) *m*/*z*: [RuL_3_](PF_6_)^+^ calcd for [C_42_H_36_F_6_N_12_PRu]^+^ 995.1866; found 995.1874, [RuL_2_]^2+^ calcd for [C_42_H_36_N_12_Ru]^2+^ 405.1113; found 405.115; Anal. calcd for C_42_H_36_F_12_N_12_P_2_Ru: C, 45.87, H, 3.30, N, 15.28; found: C, 45.52, H, 3.34, N, 15.00.

## Data Availability

The data underlying this study are available in the published article and its Supporting Information.

## Supporting Information Available

Synthesis; ^1^H and ^13^C{H} NMR spectroscopy; single X-ray diffraction data; cyclic voltammetry; differential pulsed voltammetry; UV/vis/NIR and EPR spectroelectrochemistry; (TD)DFT calculations; and photophysical measurments (PDF)
