# Supplementary material for: Impact of Bidentate Pyridyl-Mesoionic Carbene Ligands: Structural, (Spectro)Electrochemical, Photophysical, and Theoretical Investigations on Ruthenium(II) Complexes
Source: ACS Org Inorg Au. 2023 May 3;3(4):184–98. doi: 10.1021/acsorginorgau.3c00005 (PMC10401885; doi:10.1021/acsorginorgau.3c00005)
Supplement: Supplementary file 1 — gg3c00005_si_001.pdf [file gg3c00005_si_001.pdf]

The Impact of Bidentate Pyridyl-Mesoionic Carbene Ligands: Structural, (Spectro)Electrochemical, Photophysical, and Theoretical Investigations on Ruthenium(II) Complexes

Tobias Bens,[†,§] Jasmin A. Kübler,[‡] Robert R. M. Walter,[†,§] Julia Beerhues,[†,§,^] Oliver S. Wenger,[‡] Biprajit Sarkar[†,§,*]

[†]Institut für Anorganische Chemie, Universität Stuttgart, Pfaffenwaldring 55, D-70569 Stuttgart, Germany, Email: biprajit.sarkar@iac.uni-stuttgart.de

[§]Institut für Chemie und Biochemie, Freie Universität Berlin, Fabeckstraße 34-36, 14195, Berlin, Germany.

[^]Current Adress: Institute of Chemical Research of Catalonia (ICIQ), Barcelona Institute of Science and Technology (BIST), Av. Països Catalans 16, 43007 Tarragona, Spain.

[‡]Department of Chemistry, University of Basel, 4056 Basel, Switzerland; Email: oliver.wenger@unibas.ch

Keywords: mesoionic carbenes, (spectro)electrochemistry, photochemistry, ruthenium, bipyridine, metal to ligand charge transfer

1. Experimental Section 3
1.10 General Procedures, Materials and Instrumentation 3
1.20 X-ray Diffraction 4
1.30 Electrochemistry 4
1.40 Spectroelectrochemistry 5
1.50 DFT 5
1.60 Photophysical Measurements 7
2. Synthesis and Characterization 8
2.10 Synthetic Strategy for [HL]BF_4 8
2.20 Synthetic Strategy for $[RuL_1]^{2+}$, $[RuL_2]^{2+}$ and $[RuL_3]^{2+}$ 9
2.30 Preparation of $[RuL_1](PF_6)_2$ 11
2.40 Preparation of $[RuL_2](PF_6)_2$ 13
2.50 Preparation of $[RuL_3](PF_6)_2$ 18
3. Single-Crystal X-Ray Diffraction Data & Crystal Structures 20
4. Cyclic Voltammetry and Differential Pulse Voltammetry 24
4.10 Cyclic Voltammetry and Differential Pulse Voltammetry of $[RuL_1](PF_6)_2$ 24
4.20 Cyclic Voltammetry and Differential Pulse Voltammetry of $[RuL_2](PF_6)_2$ 25
4.30 Cyclic Voltammetry and Differential Pulse Voltammetry of $[RuL_3](PF_6)_2$ 26
5. EPR-Spectroelectrochemistry and Spin Density Calculations of $[RuL_1](PF_6)_2$, $[RuL_2](PF_6)_2$ and $[RuL_3](PF_6)_2$ 28
5.10 EPR-Spectroelectrochemistry and Spin Density Calculations of $[RuL_1](PF_6)_2$ 28
5.20 EPR-Spectroelectrochemistry and Spin Density Calculations of $[RuL_2](PF_6)_2$ 29
5.30 EPR-Spectroelectrochemistry and Spin Density Calculations of $[RuL_3](PF_6)_2$ 31
6. UV/vis/NIR-Spectroelectrochemistry of $[RuL_1](PF_6)_2$ and $[RuL_3](PF_6)_2$ 32
6.10 UV/vis/NIR-Spectroelectrochemistry of $[RuL_1](PF_6)_2$ 32
6.20 UV/vis/NIR-Spectroelectrochemistry of $[RuL_2](PF_6)_2$ 33
6.30 UV/vis/NIR-Spectroelectrochemistry of $[RuL_3](PF_6)_2$ 34
7. (TD)-DFT of $[RuL_1](PF_6)_2$, $[RuL_2](PF_6)_2$ and $[RuL_3](PF_6)_2$ 34
7.10 (TD)-DFT of $[RuL_1](PF_6)_2$ 34
7.20 (TD)-DFT of $[RuL_2](PF_6)_2$ 48
7.30 (TD)-DFT of $[RuL_3](PF_6)_2$ 63
7.40 Latimer Diagram 72
8. Photophysical Measurments 73
8.10 Photophysic of $[RuL_1](PF_6)_2$ 73
8.20 Photophysic of $[RuL_2](PF_6)_2$ 75

8.30 Photophysic of $[RuL_3](PF_6)_2$ 77

1. Experimental Section

1.10 General Procedures, Materials and Instrumentation

Caution! *Compounds containing azides are potentially explosive. Although we never experienced any problems during synthesis or analysis, all compounds should be synthesized only in small quantities and handled with great care!*

Unless otherwise noted, all reactions were carried out using standard Schlenk-line-techniques under an inert atmosphere of argon (Linde Argon 4.8, purity 99.998%). All reactions, which require heating were performed with an oil bath.

Commercially available chemicals were used without further purification. The solvents used for metal complex synthesis and catalysis were available from MBRAUN MB-SPS-800 solvent System and degassed by standard techniques prior to use. The identity and purity of compounds were established *via* ^{1}H and ^{13}C NMR spectroscopy, elemental analysis and mass spectrometry.

Solvents for cyclic voltammetry and UV/vis- and EPR-spectroelectrochemical measurements were dried and distilled under argon and degassed by common techniques prior to use. Column chromatography was performed over silica 60 M (0.04 – 0.063 mm).

^{1}H and ^{13}C{^{1}H} NMR spectra were recorded on a Bruker Avance 500 spectrometer at 19 – 22 °C. Chemical shifts are reported in ppm referenced to the residual solvent peaks.[1]

The following abbreviations are used to represent the multiplicity of the signals: s (singlet), d (doublet), t (triplet), q (quartet), p (pentet), sept (septet).

Mass spectrometry was performed on an Agilent 6210 ESI-TOF.

Elemental analyses were performed with an Elementar Micro Cube elemental analyser.

[1] S. Budavari (Ed.) *The Merck-index. An encyclopedia of chemicals, drugs, and biologicals*, Rahway, NJ, **1991**.

1.20 X-ray Diffraction

X-ray data were collected on a BRUKER Smart AXS, BRUKER D8 Venture or Bruker Kappa Apex2duo system. Data were collected at 100(2) or 140(2) K, respectively, using graphite-monochromatic Mo K_α radiation ($\lambda_\alpha = 0.71073$ Å). The strategy for the data collection was evaluated by using the APEX2 or Smart software. The data were collected by standard "ω scan techniques" or "$\omega - \varphi$ scan techniques" and were scaled and reduced using APEX2, SAINT+, and SADABS software. The structures were solved by direct methods using SHELXL-97 or intrinsic phasing using SHELXL-2014/7 and refined by full matrix least-squares with SHELXL-2014/7, refining on F^2. Non-hydrogen atoms were refined anisotropically. If it is noted, bond length and angles were measured with Diamond Crystal and Molecular Structure Visualization, version 3.1.[2]

[2] a) G. M. Sheldrick, *Acta Cryst.*, **2015**, *C71,* 3; b) G. M. Sheldrick, G.M. *Acta Cryst.*, **2008**, *A64,* 112–122; c) Sheldrick, G.M. *SHELXS-97 and SHELXL-97, Program for Crystal Structure Solution and Refinement;* University of Gottingen: Göttingen, Germany, **1997**; d) G. M. Sheldrick. *SHELXL Version 2014/7, Program for Crystal Structure Solution and Refinement.* University of Göttingen: Göttingen, Germany, **2014**; e) SAINT+ *Data Integration Engine,* Version 8.27b ©; Bruker AXS Inc.: Madison, Wisconsin, USA, **1997-2012**; f) Sheldrick, G.M. *Program for Empirical Absorption Correction,* SADABS Version 2008/1; University of Göttingen: Göttingen, Germany, **2008**; g) *APEX3,* v2015.5-2; Bruker AXS Inc: Madison, Wisconsin, USA, **2015**.

1.30 Electrochemistry

Cyclic voltammograms were recorded with a Metrohm Autolab potentiostat (PGSTAT 204) with a conventional three-electrode configuration consisting of a glassy carbon working electrode, a platinum auxiliary electrode, and a coiled silver wire as a pseudo reference electrode. The (decamethyl)ferrocene/(decamethyl)ferrocenium couple was used as internal reference. All measurements were performed at room temperature with a scan rate between 25 and 1000 mVs^{-1}. The experiments were carried out in absolute Acetonitrile containing 0.1 M Bu_4NPF_6 (Sigma Aldrich, ≥ 99.0%, electrochemical grade) as the supporting electrolyte.

1.40 Spectroelectrochemistry

UV/vis spectra were recorded with a J&M TIDAS spectrometer. UV/vis-spectroelectrochemical measurements were carried out in an optically transparent thin-layer electrochemical (OTTLE)[3] cell (CaF_2 windows) with a gold-mesh working electrode, a platinum-mesh counter electrode, and a silver-foil pseudo reference. EPR spectra at the X-band frequency (ca. 9.5 GHz) were obtained with a Magnettech MS-5000 benchtop EPR spectrometer equipped with a rectangular TE 102 cavity and a TC HO4 temperature controller. The measurements were carried out in synthetic quartz glass tubes. For EPR spectroelectrochemistry, a three-electrode setup was employed using two Teflon-coated platinum wires (0.005 in. bare and 0.008 in. coated) as the working and counter electrodes and a Teflon-coated silver wire (0.005 in. bare and 0.007 in coated) as the pseudo reference electrode. The low temperature EPR-spectra were performed at −175 °C. The experiments were carried out in absolute Acetonitrile or CH_2Cl_2 containing 0.1 M Bu_4NPF_6 as the supporting electrolyte. The same solvents as for the CV measurements were used for each compound.

[3] a) J. Klein, A. Stuckmann, S. Sobottka, L. Suntrup, M. van der Meer, P. Hommes, H.-U. Reissig, B. Sarkar, *Chem. Eur. J.* **2017**, *23*, 12314; b) M. Krejčik, M. Daněk, M., F. Hartl, *J. Electroanal. Chem. Interf. Electrochem.* **1991**, *317*, 179.

1.50 DFT

The program package ORCA 4.1. was used for all DFT calculations.[4] Starting from the molecular structure obtained from X-ray diffraction geometry optimizations were carried out using the PBE0[5] functional and no symmetry restrictions were imposed during the optimization. All calculations were performed with empirical Van der Waals correction (D3).[6] The restricted and unrestricted DFT methods were employed for closed and open shell molecules respectively unless stated otherwise. Convergence criteria were set to default for geometry-optimization (OPT), and tight for SCF calculations (TIGHTSCF). Triple-ζ–valence basis sets (def2-TZVP)[7] were employed for all atoms. Calculations were performed using resolution of the identity approximation[8] with matching auxiliary basis sets[9] for geometry optimizations and numerical frequency calculations and the RIJCOSX (combination of the resolution of the identity and chain of spheres algorithms) approximation for single point calculations using the PBE0 functional.[8] Low-lying excitation energies were calculated with time-dependent DFT (TD-DFT).

Solvent effects were taken into account with the conductor-like polarizable continuum model, CPCM.[10] Spin densities were calculated according to the Mulliken population analysis.[11] The absence of imaginary frequency Spin densities, molecular orbitals and difference densities were visualized with the modified Chemcraft 1.8 program.[12] All molecular orbitals are illustrated with an iso value of 0.052. All calculated TD-DFT spectra are Gaussian broadened with a band width of 25 nm at half height.

[4] F. Neese, *Wiley Interdiscip. Rev. Comput. Mol. Sci*. **2012**, *2*, 73.

[5] C. Adamo, V. Barone; Toward Reliable Density Functional Methods without Adjustable Parameters: The PBE0Model. *J. Chem. Phys*. **1999**, *110*, 6158–6170.

[6] a) S. Grimme, S. Ehrlich, L. Goerigk, *J. Comput. Chem.* **2011**, *32*, 1456; b) S. Grimme, J. Antony, S. Ehrlich, H. Krieg, *J. Chem. Phys*. **2010**, *132*, 154104; c) S. Grimme, *J. Comput. Chem*. **2004**, *25*, 1463; d) S. Grimme, *J. Comput. Chem*. **2006**, *27*, 1787.

[7] F. Weigend, R. Ahlrichs, *Phys. Chem. Chem. Phys*. **2005**, *7*, 3297.

[8] a) F. Neese, *J. Comput. Chem*. **2003**, *24*, 1740; b) F. Neese, F. Wenmohs, A. Hansen, U. Becker, *Chem. Phys.* **2009**, *356*, 98; c) O. Vahtras, J. Almloef, M. W. Feyereisen, *Chem. Phys. Lett*. **1993**, *213*, 514; d) J. L. Whitten, *J. Chem. Phys.* **1973**, *58*, 4496; e) R. Izsak; F. Neese, *J. Chem. Phys*. **2011**, *135*, 144105; f) F. Neese, G. Olbrich, *Chem. Phys. Lett*. **2002**, *362*, 170; g) T. Petrenko, S. Kossmann, F. Neese, *J. Chem. Phys.* **2011**, *134*, 054116.

[9] a) K. Eichkorn, F. Weigend, O. Treutler, R. Ahlrichs, *Theor. Chem. Acc*. **1997**, *97*, 119; b) K. Eichhorn, O. Treutler, H. Öhm, M. Häser, R. Ahlrichs, *Chem. Phys. Lett*. **1995**, *242*, 652.

[10] V. Barone, M. Cossi *J. Phys. Chem. A* **1998**, *102*, 1995.

[11] R. S. Mulliken, *J. Chem. Phys*. **1955**, *23*, 1833.

[12] Zhurko, G.A.; *Chemcraft-Graphical Program for Visualization of Quantum Chemistry Computations,* Ver. 1.8. Available Online: http://www.chemcraftprog.com/: Ivanovo, Russia, **2023**.

1.60 Photophysical Measurements

Steady-state luminescence spectra at room temperature and 77 K were measured using a Fluorolog-3-22 instrument from Horiba Jobin-Yvon. Transient absorption and kinetic emission and absorption measurements were performed on a LP920-KS instrument from Edinburgh Instruments in MeCN. Excitation source was a pulsed Quantel Brilliant b ND:YAG laser equipped with a Rainbow optical parameter oscillator (OPO) with a pulse energy of 13 mJ at 435 nm and 20 mJ at 460 nm. The solutions typically had an optical density below 0.4 and were deaerated through three cycles of freeze-pump-thaw.

The quantum yields were measured on a Hamamatsu absolute photoluminescence quantum yield spectrometer C11347 Quantaurus QY with a concentration of $2.5 \cdot 10^{-5}$ M and the solutions were deaerated by bubbling Ar through for 10 min.

2. Synthesis and Characterization

2.10 Synthetic Strategy for [HL]BF_4

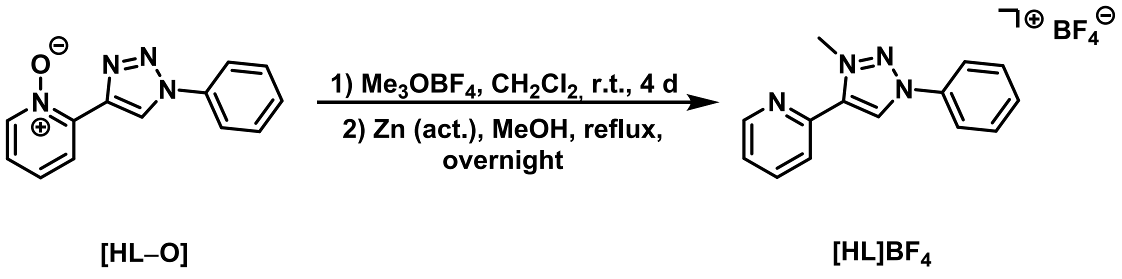

Scheme S1: Synthetic Strategy for **[HL]BF_4**.

According to a modified procedure the triazole-containing *N*-Oxide **[HL–O]** (1.04 g, 3.21 mmol) was dissolved in 20 mL mL CH_2Cl_2. Me_3OBF_4 (1.04 g, 7.03 mmol) was added and the mixture was stirred at room temperature for 5 days. The solvent was evaporated and the residue suspended in abs. MeOH (80 mL). Activated zinc (0.835 g, 12.84 mmol) was added and heated under reflux overnight. The reaction mixture was filtered through celite and purified by column chromatography CH_2Cl_2/MeOH (10:1) to obtain the product as white-off solid (0.74 g, 2.92 mmol, 91%).

^{1}H NMR (400 MHz, CD_3CN) δ (ppm) = 9.23 (s, 1H), 8.89 – 8.82 (m, 1H), 8.10 (td, *J* = 7.8, 1.7 Hz, 1H), 8.03 – 7.89 (m, 3H), 7.80 – 7.72 (m, 3H), 7.68 – 7.62 (m, 1H), 4.66 (s, 3H); **^{13}C {^{1}H} NMR** (101 MHz, CD_3CN) δ (ppm) = 151.4, 143.9, 142.7, 139.4, 136.0, 133.2, 131.6, 128.3, 127.2, 125.7, 122.7, 42.1; **Anal. calcd. for $C_{14}H_{13}BF_4N_4$:** C, 51.89, H, 4.04, N, 17.29; **found:** C, 51.99, H, 4.15, N, 17.29.[13]

2.20 Synthetic Strategy for $[RuL_1]^{2+}$, $[RuL_2]^{2+}$ and $[RuL_3]^{2+}$

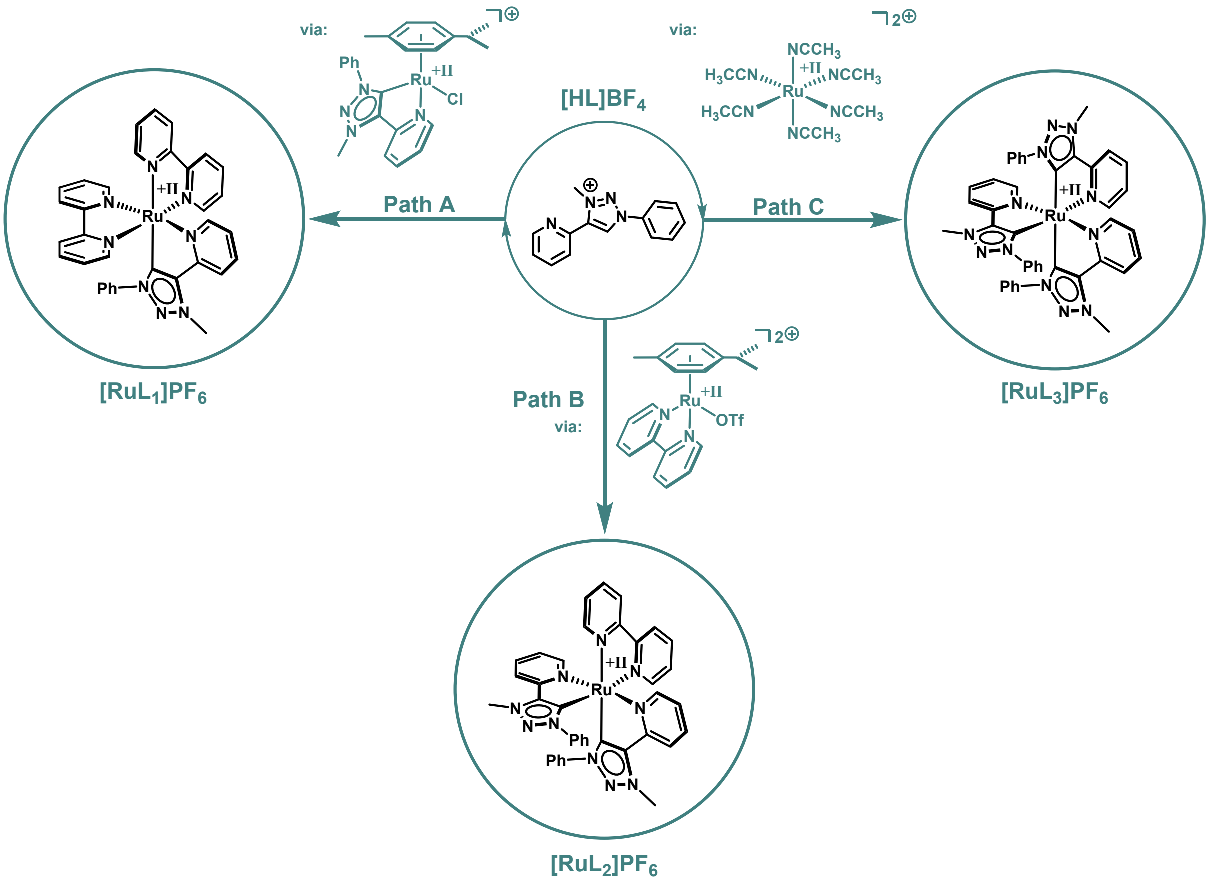

Scheme S2. Synthetic strategy for $\mathbf{[RuL_1]^{2+}}$, $\mathbf{[RuL_2]^{2+}}$ and $\mathbf{[RuL_3]^{2+}}$. **Path A: [HL]BF$_4$,** Ag_2O, CH_3CN, rt, 4 d; [Ru(*p*-cymene)Cl_2]$_2$, rt, 2 h (74%);[14] bpy, $AgPF_6$, ethylene glycol, 150 °C, 12 h; aq. KPF_6 (74%).[15] **Path B:** [Ru(*p*-cymene)Cl_2]$_2$, bpy, MeOH, 2 h, rt; aq. NH_4PF_6, 1 h (72%);[16] HOTF, DCM, rt, 12 h (83%);[17] **[HL]BF$_4$**, ethylene glycol, 180 °C, 12 h; aq. KPF_6 (49%, crude); 2 weeks under *hv* in acetone/Et_2O (16%).[18] **Path C**: *modified*: $RuCl_3 \cdot 3\ H_2O$, Zn(act), CH_3CN, reflux, 2 d; $AgBF_4$, CH_3CN, reflux, 12 h (81%);[18] *modified*: **[HL]BF$_4$**, K_2CO_3, ethylene glycol, 160 °C, 16 h; aq. NH_4PF_6 (46%).[19]

[13] (a) **Ligandsynthesis**: A. Bolje, J. Košmrlj, *Org. Lett.* **2013***, 15*, 19, 5084; (b) Y. B. R. D. Rajesh, *J. Heterocyclic Chem.* **2018**, *55*, 486.

[14] A. Bolje, S. Hohloch, D. Urankar, A. Pevec, M. Gazvoda, B. Sarkar, J. Košmrlj, *Organometallics* **2014**, *33*, 2588.

[15] L. Suntrup, F. Stein, G. Hermann, M. Kleoff, M. Kuss-Petermann, J. Klein, O. S. Wenger, J. C. Tremblay, B. Sarkar*, Inorg. Chem*. **2018**, *57*, 13973.

[16] G. K. Mutua, R. Bellam, D. Jaganyi, A. Mambanda, *J. Coord. Chem.* **2019**, *72*, 2931.

[17] N. Queyriaux, E. Giannoudis, J.-F. Lefebvre, V. Artero, M. Chavarot-Kerlidou, *Eur. J. Inorg. Chem.* **2019**, *2019*, 2154.

[18] M. A. Abbas, C. D. McMillien, J. L. Brumaghim, *Inorg. Chim. Acta* **2017**, *468*, 308.

[19] S. U. Son, K. H. Park, Y.-S. Lee, B. Y. Kim, C. H. Choi, M. S. Lah, Y. H. Jang, D.-J. Jang, Y. K. Chung, *Inorg. Chem*. **2004**, *43*, 6896.

2.30 Preparation of [RuL$_1$](PF$_6$)$_2$

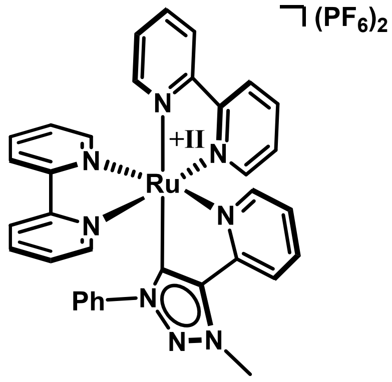

In a 15 mL Schlenk flask, the respective [Ru(**L**)(*p*-cymene)Cl]BF$_4$ (50 mg, 0.084 mmol, 1 eq.), bipyridine (26 mg, 0.169 mmol, 2 eq.) and AgPF$_6$ (43 mg, 0.169 mmol, 2 eq.) were dissolved in degassed ethylene glycol (4 mL). The reaction mixture was capped and heated to 150 °C for 12 h. After cooling to room temperature, the resulting dark orange mixture was treated with aqueous KPF$_6$ and extracted with CH$_2$Cl$_2$ (3 x 40 mL). The organic phase was washed with water (5 x 50 mL) and dried over Na$_2$SO$_4$. Additional crystallization from slow diffusion of Et$_2$O into a concentrated solution of **[RuL$_1$](PF$_6$)$_2$** dissolved in CH$_2$Cl$_2$ yielded in a orange crystalline solid of **[RuL$_1$](PF$_6$)$_2$** (57 mg, 0.061 mmol, 72%). In case of an insufficient conversion, purification by column chromatography (aluminum oxide, activated with 5 w% water; CH$_2$Cl$_2$/acetone 100:0 → 100:15) resulted in pure **[RuL$_1$](PF$_6$)$_2$**. Single crystals suitable for X-ray diffraction were obtained by slow vapor diffusion of Et$_2$O into a concentrated solution of **[RuL$_1$](PF$_6$)$_2$** in acetone at 4 °C.

^{1}H NMR (500 MHz, CD$_3$CN) δ (ppm) = 8.45 (d, *J* = 8.2 Hz, 1H), 8.42 (d, *J* = 8.1 Hz, 1H), 8.35 (d, *J* = 5.6 Hz, 1H), 8.27 (d, *J* = 8.1 Hz, 1H), 8.16 (d, *J* = 8.1 Hz, 1H), 8.06 – 7.95 (m, 5H), 7.70 – 7.68 (m, 1H), 7.67 (d, *J* = 5.6 Hz, 1H), 7.61 – 7.56 (m, 2H), 7.43 – 7.36 (m, 3H), 7.27 – 7.21 (m, 2H), 7.20 – 7.16 (m, 1H), 7.08 (t, *J* = 8.0 Hz, 2H), 6.92 (dd, *J* = 8.4, 1.1 Hz, 2H), 6.82 (ddd, *J* = 7.3, 5.7, 1.3 Hz, 1H), 4.58 (s, 3H); **^{13}C{^{1}H} NMR** (126 MHz, CD$_3$CN) δ (ppm) = 185.6, 158.0, 157.6, 157.5, 156.5, 156.5, 153.9, 153.0, 152.5, 152.1, 150.5, 146.8, 139.2, 139.0, 138.5, 138.1, 137.7, 137.2, 131.2, 130.3, 128.6, 128.3, 128.0, 127.4, 126.0, 125.9, 125.1, 124.8, 124.4, 124.1, 122.6, 39.9; **MS (ESI)**: m/z found: 795.10, calcd: 795.1038 (C$_{34}$H$_{27}$F$_6$N$_8$PRu$^+$), found: 325.07, calcd: 325.5774 (C$_{34}$H$_{28}$N$_8$Ru^{2+}); **Anal. calcd. for C$_{34}$H$_{28}$F$_{12}$N$_8$P$_2$Ru:** C, 43.46, H, 3.00, N, 11.93; **found:** C, 43.60, H, 3.06, N, 11.60.

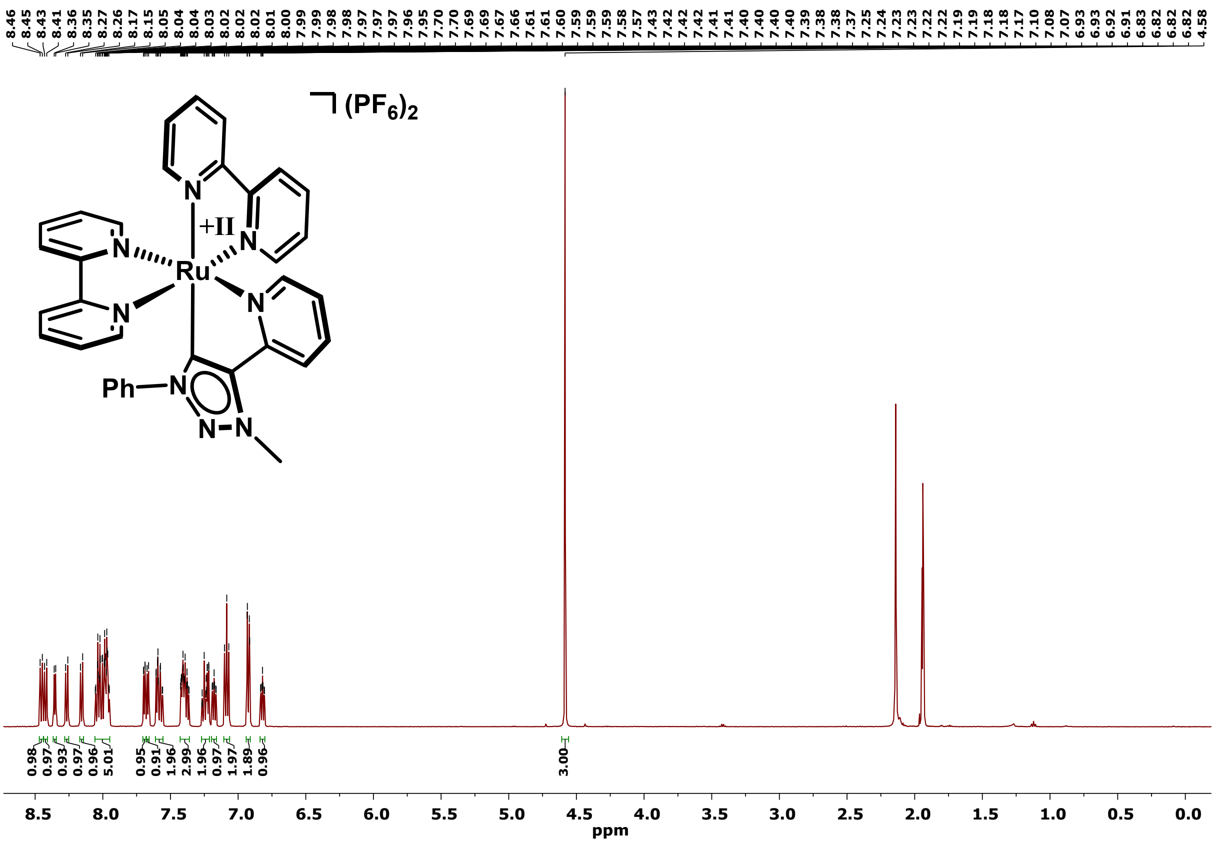

Figure S1. ^{1}H NMR (500 MHz, CD_3CN) spectrum of **[RuL$_1$](PF$_6$)$_2$**.

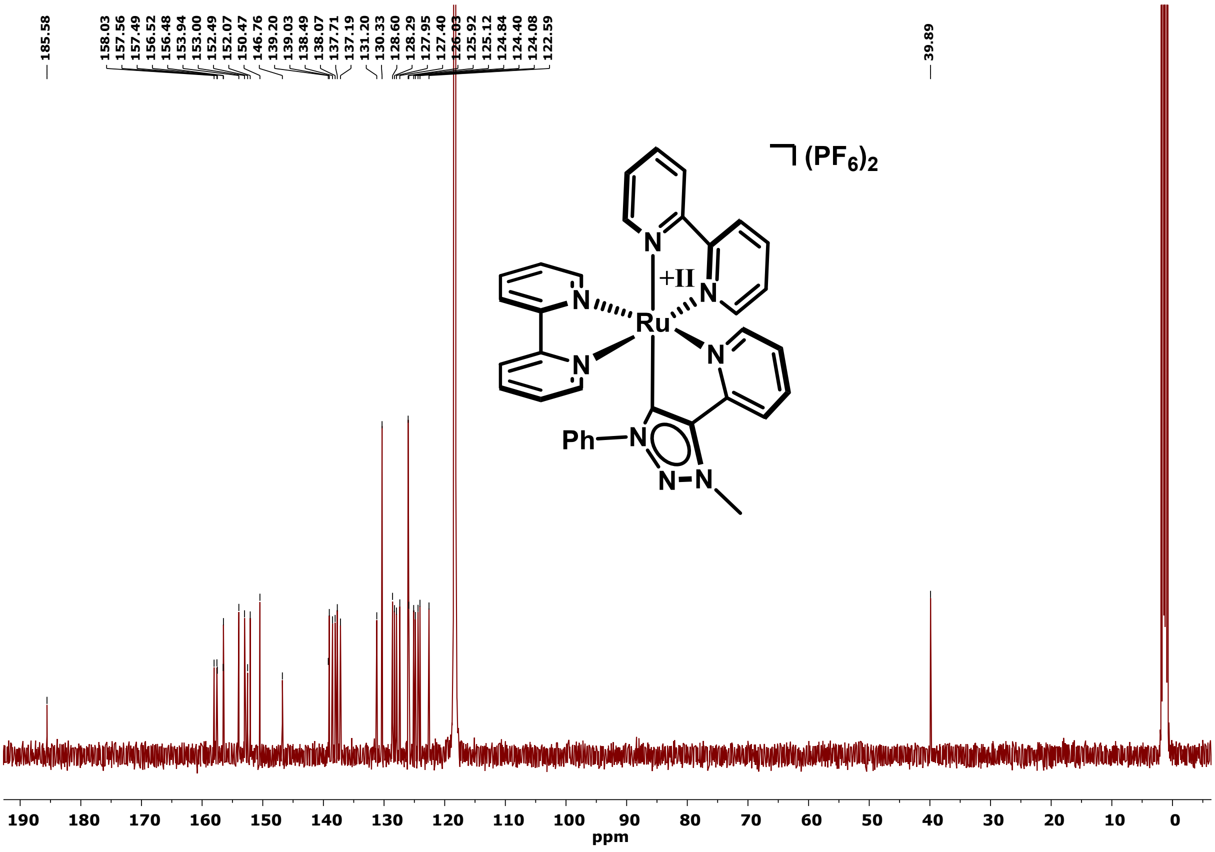

Figure S2. ^{13}C{H} NMR (126 MHz, CD_3CN) spectrum of **[RuL$_1$](PF$_6$)$_2$**.

2.40 Preparation of $[RuL_2](PF_6)_2$

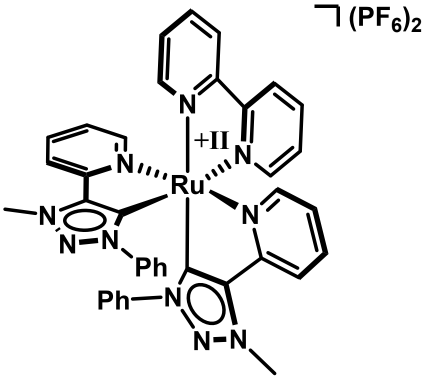

In a 15 mL Schlenk flask, [Ru(bpy)(*p*-cymene)OTf]OTf (34 mg, 0.049 mmol, 1 eq.) and [H**L**]BF_4 (30 mg, 0.093 mmol, 2 eq.) were dissolved in degassed ethylene glycol (4 mL). The reaction mixture was capped and heated to 180 °C for 12 h. After cooling to room temperature, the resulting dark orange mixture was treated with aqueous KPF_6 and extracted with CH_2Cl_2 (3 x 40 mL). The organic phase was washed with water (5 x 50 mL) and dried over Na_2SO_4. The crude product was dry loaded on Celite and purified by column chromatography (inversed column, interchim puriFlash XS 520 Plus, column: PF-15C18AQ-F0040; H_2O/CH_3CN 100:0 → 80:20). The crude product was extracted with CH_2Cl_2 (3 x 40 mL) and dried over Na_2SO_4. The solvent was removed under reduced pressure and the remaining dark orange solid (23 mg, 0.022 mmol, 46% crude) was dissolved in $(CH_3)_2CO$ (3 mL) and overlayed with Et_2O under light. The dark orange solution turned dark brown after two days and after one-month single crystals suitable for x-ray diffraction have been obtained. The collected crystals were used as seed crystals to induce crystallization from the crude product in the follow up reactions. The resulting red crystals (7.8 mg, 0.077 mmol, 16%) were used without further purification.

Crude Product (before crystallization): ^{1}H NMR (250 MHz, CD_3CN) δ (ppm) = 8.27 (d, *J* = 8.1 Hz, 1H), 8.14 – 8.03 (m, 2H), 8.03 – 7.88 (m, 3H), 7.76 – 7.71 (m, 1H), 7.64 (ddd, *J* = 5.5, 1.6, 0.8 Hz, 1H), 7.58 – 7.38 (m, 5H), 7.25 – 7.12 (m, 2H), 7.09 – 7.02 (m, 2H), 6.95 (t, *J* = 7.9 Hz, 2H), 6.78 (ddd, *J* = 7.4, 5.7, 1.4 Hz, 1H), 6.36 – 6.27 (m, 2H), 4.52 (s, 3H), 4.40 (s, 3H). **^{13}C{^{1}H} NMR** (126 MHz, CD_3CN) δ (ppm) = 188.3, 186.6, 157.4, 156.4, 156.3, 154.5, 154.4, 153.3, 151.8, 151.5, 151.2, 151.1, 146.5, 145.8, 139.8, 138.9, 138.8, 138.6, 138.3, 137.5, 137.1, 136.7, 131.7, 130.7, 130.1, 129.9, 128.1, 127.0, 126.1, 125.6, 125.4, 124.1, 124.0, 122.2, 122.1, 121.6, 40.0, 39.5;

MS (ESI): m/z found: 875.15, calcd: 875.1491 ($C_{38}H_{32}F_6N_{10}PRu^+$), found: 365.09, calcd: 365.0922 ($C_{38}H_{32}N_{10}Ru^{2+}$); **Anal. calcd. for $C_{38}H_{32}F_{12}N_{10}P_2Ru$:** C, 44.76, H, 3.16, N, 13.74; **found:** C, 44.55, H, 3.36, N, 13.61.

$[RuL_2](PF_6)_2$ (single crystals): **^{1}H NMR** (700 MHz, CD_3CN) δ (ppm) = 8.42 (d, *J* = 7.0 Hz, 2H), 8.29 (d, *J* = 6.5 Hz, 2H), 8.00 (td, *J* = 8.1, 1.5 Hz, 2H), 7.58 (d, *J* = 8.0 Hz, 2H), 7.53 (td, *J* = 7.9, 1.4 Hz, 2H), 7.42 (ddd, *J* = 7.5, 5.5, 1.2 Hz, 2H), 7.28 – 7.23 (m, 4H), 7.11 – 7.08 (m, 4H), 7.05 (dd, *J* = 8.4, 1.2 Hz, 4H), 6.71 (ddd, *J* = 7.3, 5.7, 1.4 Hz, 2H), 4.42 (s, 6H).

^{13}C{^{1}H} NMR (176 MHz CD_3CN) δ (ppm) = 185.1, 156.6, 154.4, 153.3, 151.6, 145.6, 139.1, 138.5, 137.0, 131.2, 130.0, 128.3, 125.9, 124.8, 124.8, 121.6, 39.6; **HRMS (ESI)** m/z: $[RuL_2](PF_6)^+$ Calcd. for $[C_{38}H_{32}F_6N_{10}PRu]^+$ 875.1491; found 875.1487, $[RuL_2]^{2+}$ Calcd. for $[C_{38}H_{32}N_{10}Ru]^{2+}$ 365.0922; found 365.0925; **Anal. calcd. for $C_{38}H_{32}F_{12}N_{10}P_2Ru$:** C, 44.76, H, 3.16, N, 13.74; **found:** C, 44.71, H, 3.20, N, 13.40.

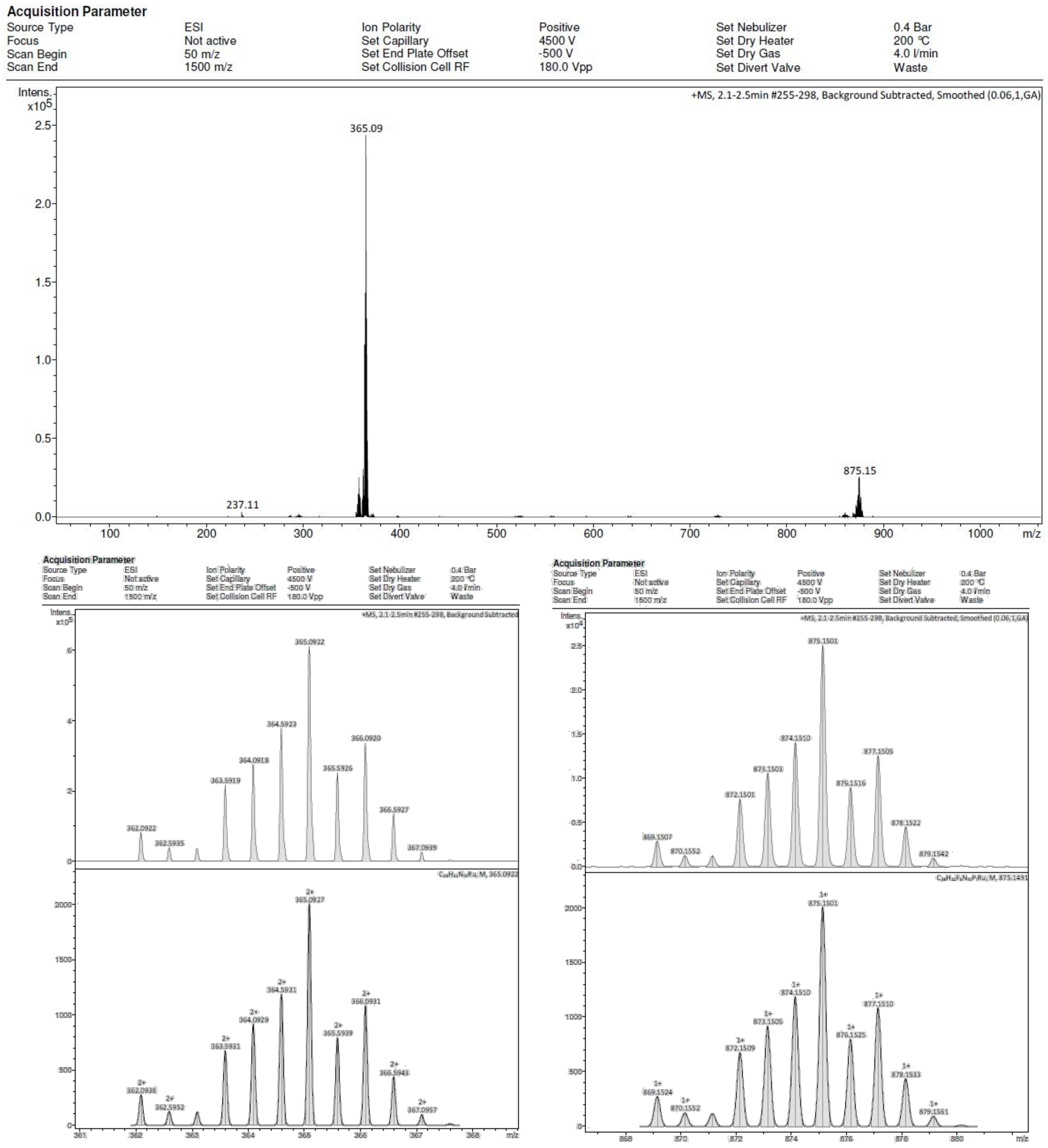

Figure S3. ESI mass spectrum of **[RuL$_2$](PF$_6$)$_2$** (top) and simulated mass spectrum of **[RuL$_2$]$^{1+}$** (bottom, right) **[RuL$_2$]$^{2+}$** (bottom, left).

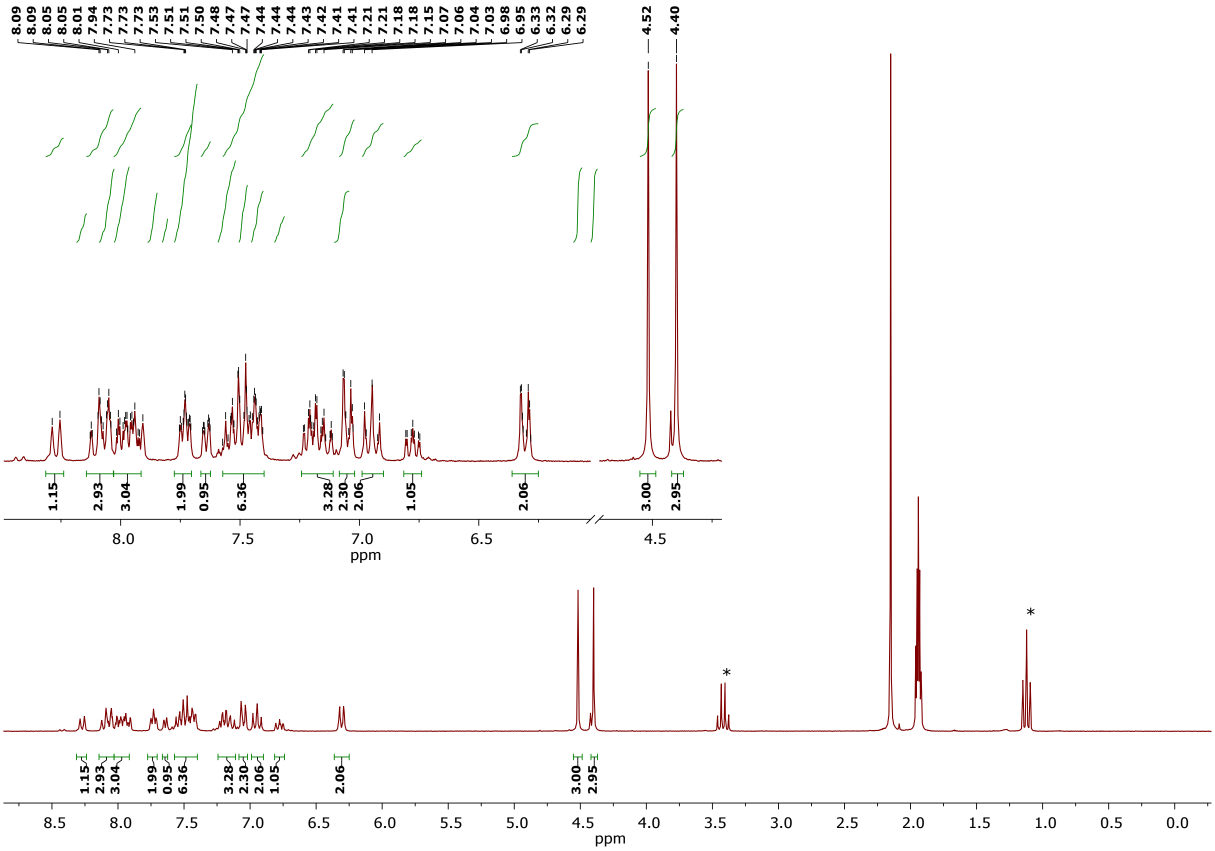

Figure S4. Best obtained ^{1}H NMR (250 MHz, CD_3CN) spectrum of crude **[RuL$_2$](PF$_6$)$_2$** (*: Et_2O). Top left: Inset of ^{1}H NMR spectrum showing crude product mixture.

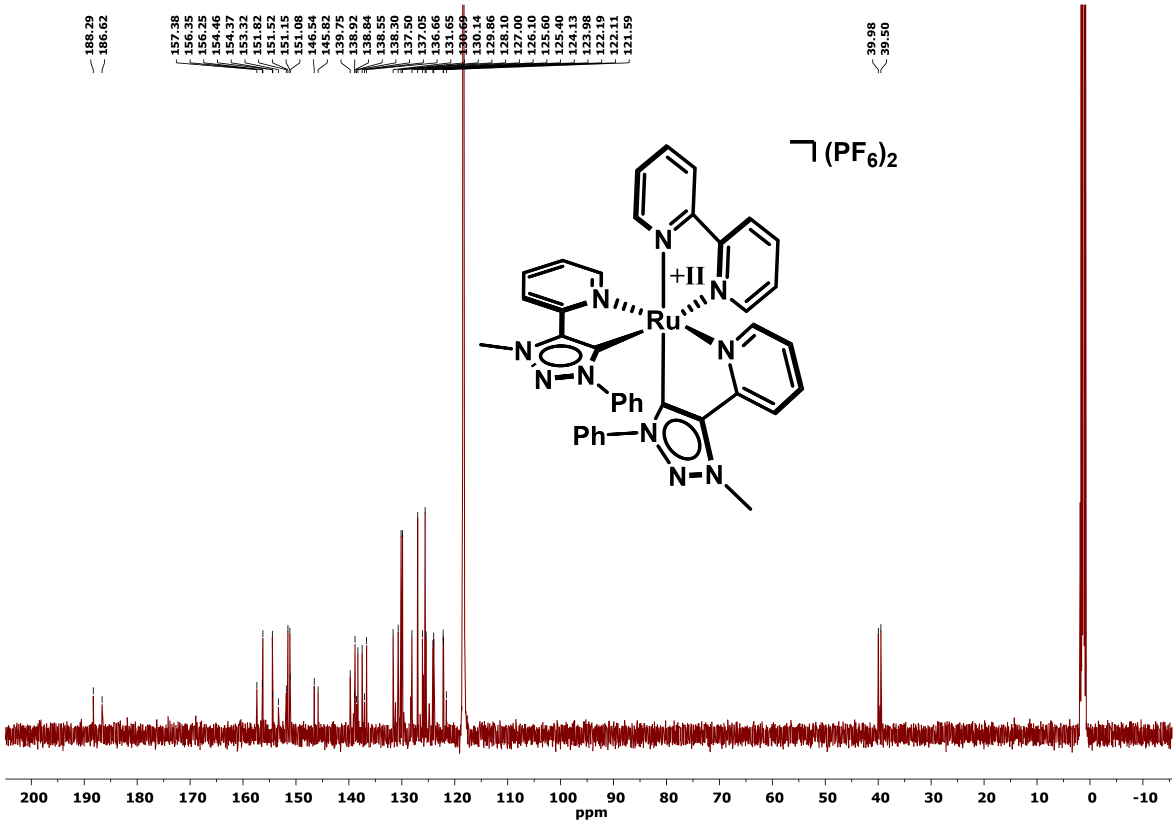

Figure S5. ^{13}C{H} NMR (126 MHz, CD_3CN) spectrum of crude **[RuL$_2$](PF$_6$)$_2$**.

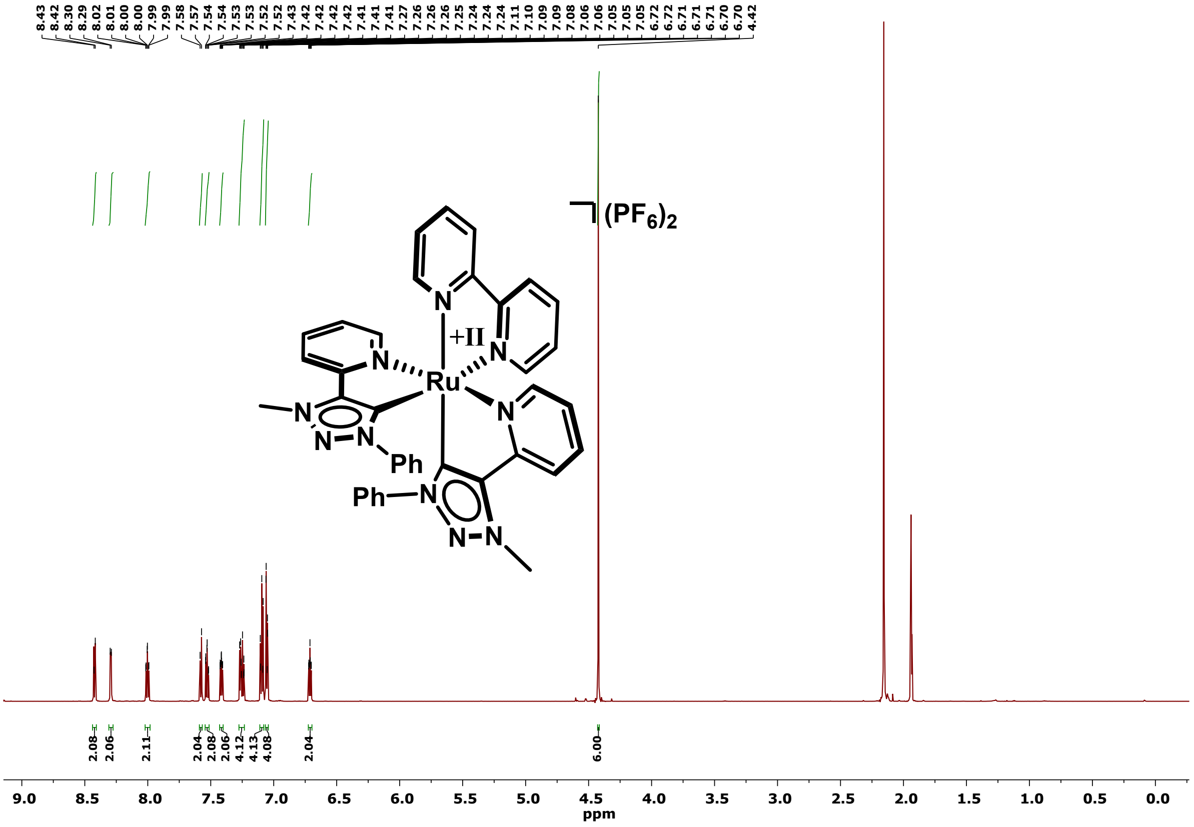

Figure S6. ^{1}H NMR (700 MHz, CD_3CN) spectrum of **$[RuL_2](PF_6)$**.

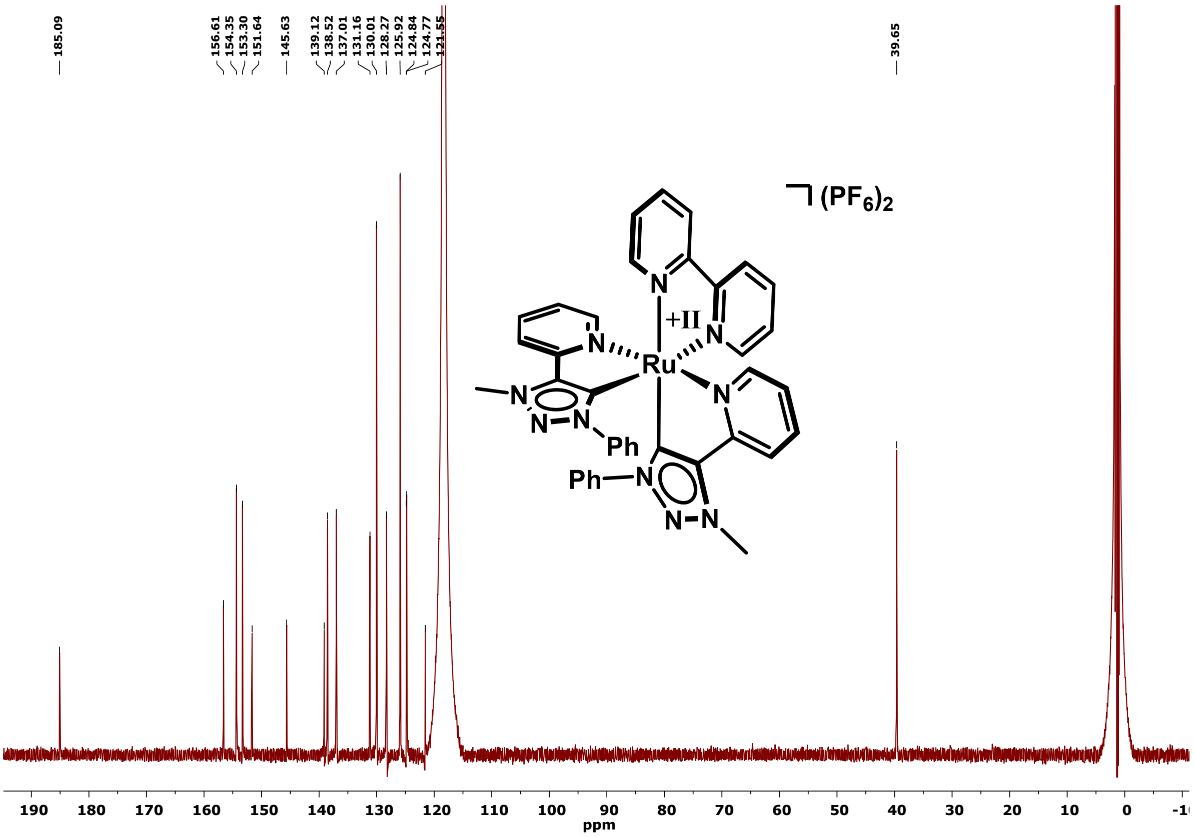

Figure S7. ^{13}C{H} NMR (176 MHz, CD_3CN) spectrum of **$[RuL_2](PF_6)$**.

2.50 Preparation of $[RuL_3](PF_6)_2$

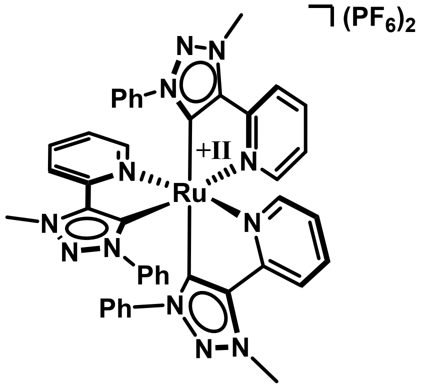

In a 15 mL Schlenk flask, $[Ru(CH_3CN)_6](BF_4)_2$ (54 mg, 0.103 mmol, 1 eq.), [H**L**]BF_4 (100 mg, 0.310 mmol, 3 eq.) and K_2CO_3 (47 mg, 0.341 mmol, 3.3 eq.) were dissolved in degassed ethylene glycol (4 mL). The reaction mixture was capped and heated to 160 °C for 16 h. After cooling to room temperature, the resulting dark red mixture was treated with aqueous NH_4PF_6 whereas a dark orange preicpitate was formed. The orange solid was filtered off and extensively washed with H_2O, EtOAc, Et_2O. The remaining solid was dissolved in acetone (15 mL), cooled down with liquid nitrogen, overlayed with Et_2O and cooled down with liquid nitrogen, too. The capped flask was stored at –20 °C for 1 month inducing crystallization of dark orange crystals suitable for X-ray diffraction analysis. The remaining crystalline solid was vigorously washed with Et_2O and decanted to remove remaining brownish solid yielding a dark orange crystalline solid of **$[RuL_3](PF_6)_2$** (52 mg, 0.047 mmol, 46%).

^{1}H NMR (500 MHz, CD_3CN) δ (ppm) = 8.25 (d, J = 5.4 Hz, 1H), 8.13 (d, J = 8.1 Hz, 1H), 7.99 (td, J = 7.9, 1.5 Hz, 1H), 7.82 (d, J = 5.5 Hz, 1H), 7.60 – 7.47 (m, 5H), 7.46 – 7.39 (m, 3H), 7.34 – 7.23 (m, 2H), 7.19 (t, J = 7.0 Hz, 1H), 7.14 – 7.04 (m, 4H), 7.04 – 6.98 (m, 4H), 6.75 (dddd, J = 13.2, 7.3, 5.8, 1.8 Hz, 2H), 6.51 (dd, J = 8.4, 1.1 Hz, 2H), 4.55 (s, 3H), 4.43 (s, 3H),4.30 (s, 3H); **^{13}C{^{1}H} NMR** (126 MHz, CD_3CN) δ (ppm) = 191.1, 187.0, 186.6, 157.8, 156.0, 153.7, 151.9, 151.4, 150.9, 147.1, 146.3, 144.1, 140.0, 139.3, 139.0, 138.4, 136.6, 135.8, 131.4, 130.8, 130.6, 129.99, 129.96, 129.6, 126.5, 126.1, 125.5, 125.3, 124.7, 124.2, 122.2, 121.49, 121.1, 39.9, 39.6, 39.3; **HRMS (ESI)** m/z: $[RuL_3](PF_6)^+$ Calcd. for $[C_{42}H_{36}F_6N_{12}PRu^+]^+$ 995.1866; found 995.1874, $[RuL_2]^{2+}$ Calcd. for $[C_{42}H_{36}N_{12}Ru]^{2+}$ 405.1113; found 405.115; **Anal. calcd. for $C_{42}H_{36}F_{12}N_{12}P_2Ru$:** C, 45.87, H, 3.30, N, 15.28; **found:** C, 45.52, H, 3.34, N, 15.00.

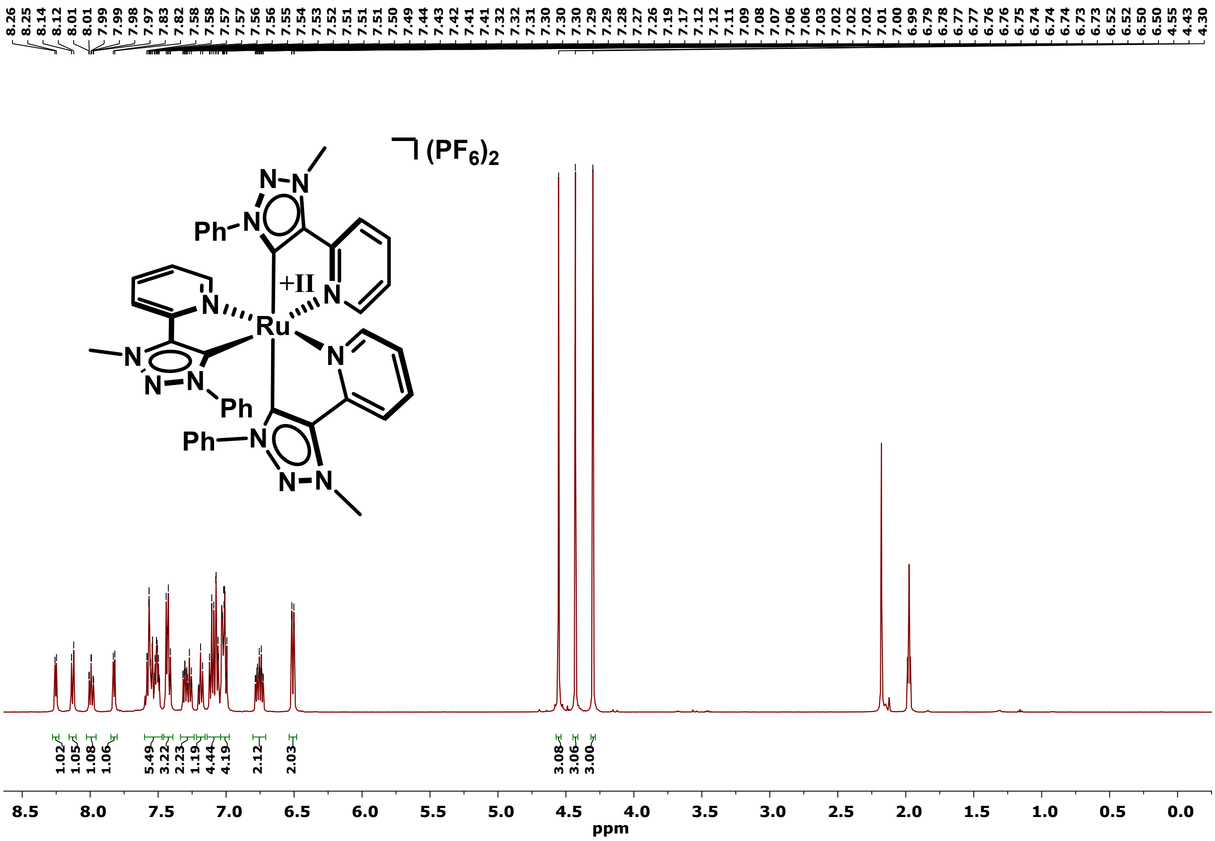

Figure S8. ^{1}H NMR (500 MHz, CD_3CN) spectrum of **$[RuL_3](PF_6)_2$**.

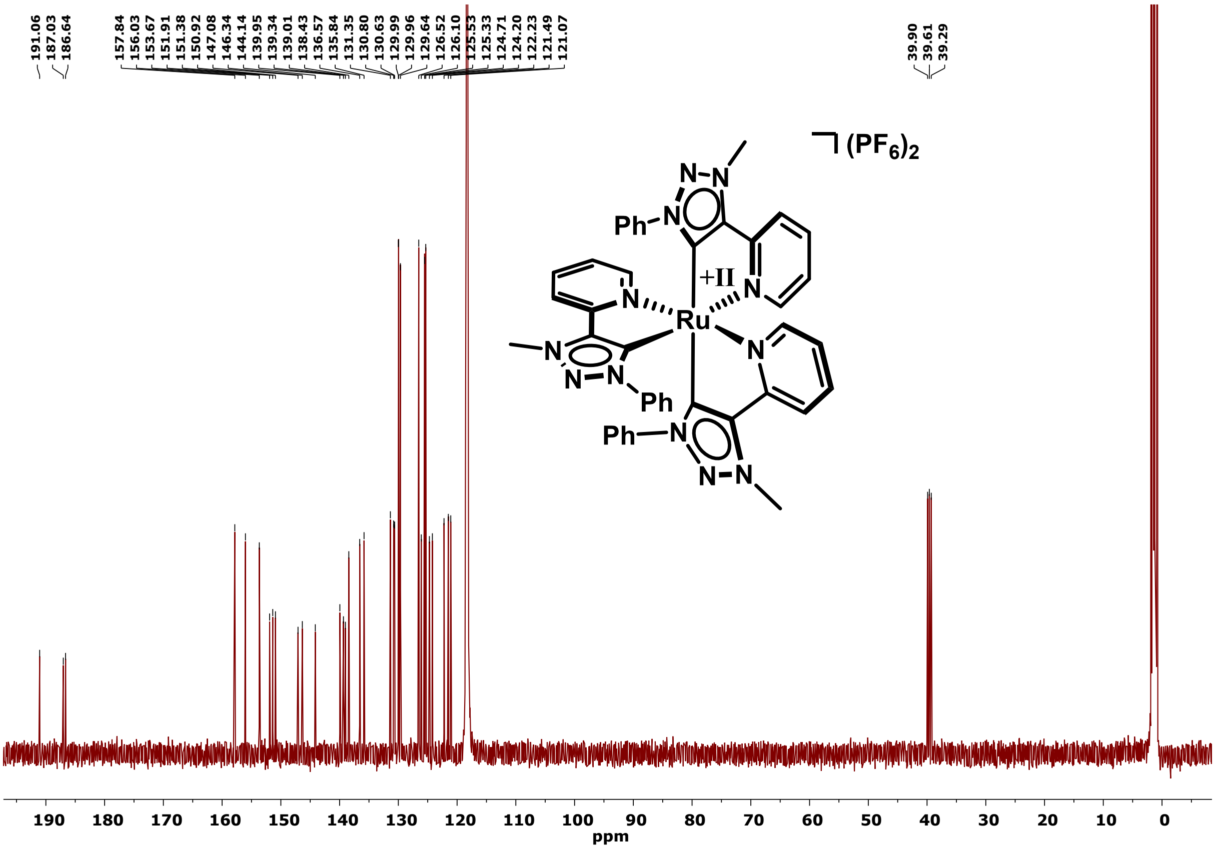

Figure S9. ^{13}C{H} NMR (126 MHz, CD_3CN) spectrum of **$[RuL_3](PF_6)_2$**.

3. Single-Crystal X-Ray Diffraction Data & Crystal Structures

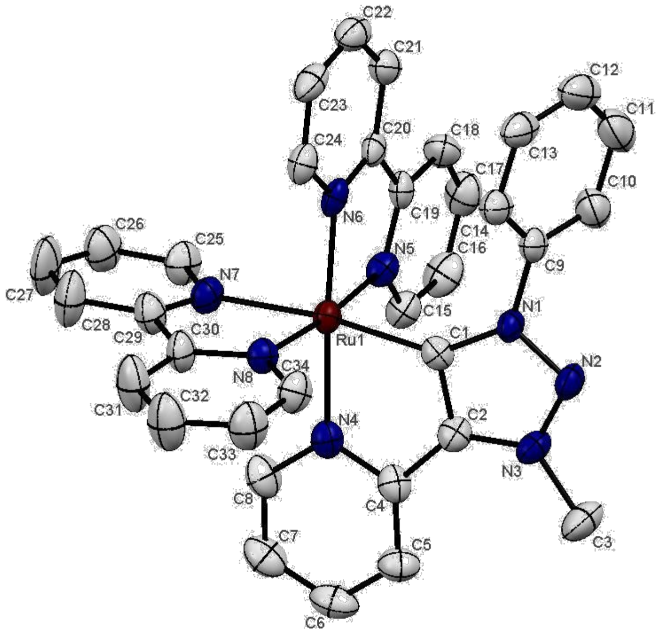

Figure S10. ORTEP representation of **[RuL$_1$](PF$_6$)$_2$** (hydrogen atoms and counter ions are omitted for clarity). Ellipsoids are drawn with 50% probability.

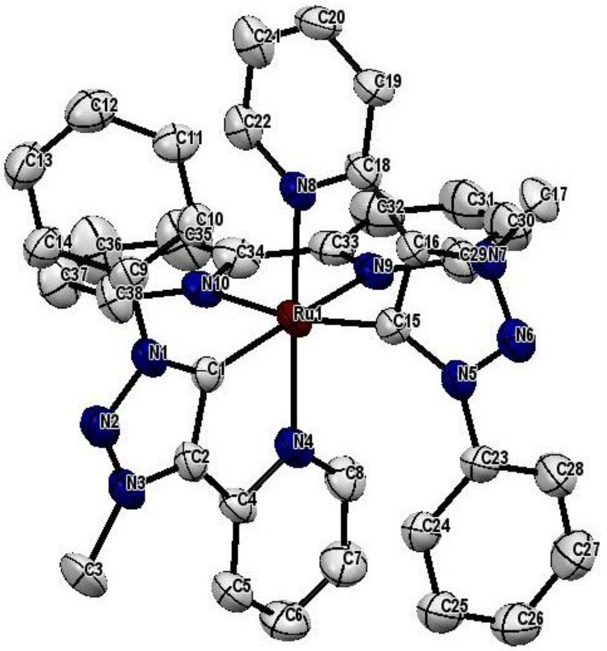

Figure S11. ORTEP representation of **[RuL$_2$](PF$_6$)$_2$** (hydrogen atoms and counter ions are omitted for clarity). Ellipsoids are drawn with 50% probability.

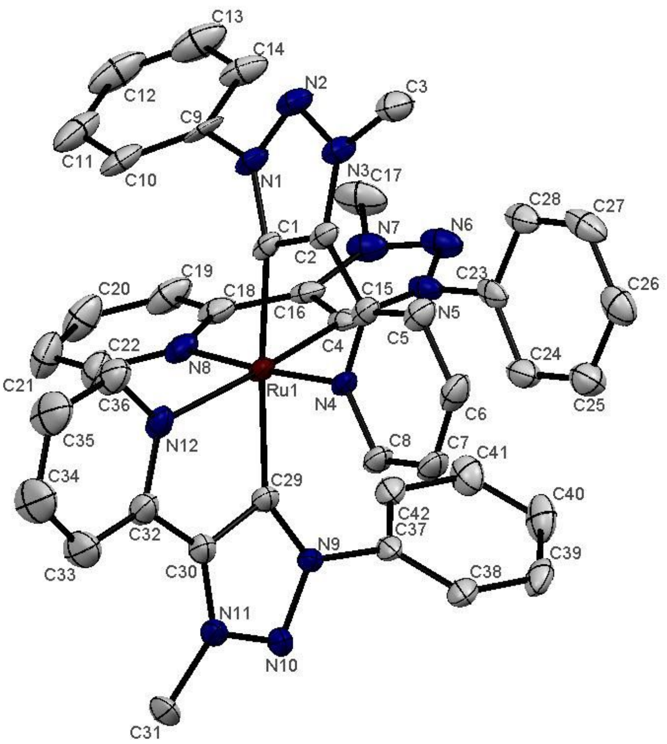

Figure S12. ORTEP representation of **$[RuL_3](PF_6)_2$** (hydrogen atoms, counter ions and solvent molecules are omitted for clarity). Ellipsoids are drawn with 50% probability.

Table S1. Selected bond lengths and angles of **$[RuL_1](PF_6)_2$** and **$[RuL_3](PF_6)_2$**.

Atoms	$[RuL_1](PF_6)_2$	Atoms	$[RuL_2](PF_6)_2$	Atoms	$[RuL_3](PF_6)_2$
**Bond lengths / Å**					
Ru1–C1	2.006(5)	Ru1–C1	2.012(5)	Ru1–C1	2.060(5)
Ru1–N4	2.061(4)	Ru1–N4	2.091(5)	Ru1–C15	1.993(4)
Ru1–N5	2.053(4)	Ru1–C15	2.012(6)	Ru1–C29	2.056(5)
Ru1–N6	2.111(4)	Ru1–N8	2.101(5)	Ru1–N4	2.113(4)
Ru1–N7	2.071(4)	Ru1–N9	2.115(4)	Ru1–N8	2.104(4)
Ru1–N8	2.114(4)	Ru1–N10	2.132(5)	Ru1–N12	2.166(4)
C1–C2	1.401(7)	C1–C2	1.389(8)	C1–C2	1.375(6)
C2–N3	1.362(7)	C2–N3	1.363(7)	C2–N3	1.358(6)
N2–N3	1.320(6)	N2–N3	1.312(7)	N2–N3	1.320(5)
N1–N2	1.352(6)	N1–N2	1.355(6)	N1–N2	1.356(6)
N1–C1	1.436(7)	N1–C1	1.370(7)	N1–C1	1.375(6)
C2–C4	1.401(7)	C2–C4	1.440(9)	C2–C4	1.453(6)
C4–N4	1.363(7)	C4–N4	1.381(7)	C4–N4	1.367(6)
N4–C8	1.358(7)	N4–C8	1.345(8)	N4–C8	1.355(6)
C8–C7	1.380(8)	C8–C7	1.376(9)	C8–C7	1.387(7)
C7–C6	1.384(9)	C7–C6	1.376(1)	C7–C6	1.382(8)
C6–C5	1.385(8)	C6–C5	1.376(1)	C6–C5	1.382(7)

**Bond lengths / Å**					
C5–C4	1.386(7)	C5–C4	1.392 (9)	C5–C4	1.384(7)
N5–C19	1.358(7)	N8–C18	1.380 (7)	C15–C16	1.397(7)
C19–C20	1.476(7)	C18–C16	1.441 (8)	C16–C18	1.451(7)
C20–N6	1.349(7)	C16–C15	1.390 (8)	C18–N8	1.380(6)
N7–C29	1.350(7)	N9–C33	1.348 (8)	C29–C30	1.393(6)
C29–C30	1.472(8)	C33–C34	1.482 (9)	C30–C32	1.458(7)
C30–N8	1.360(7)	C34–N10	1.366 (7)	C32–N12	1.368(7)
**Bond angles / °**					
C1–Ru1–N7	172.4(2)	C1–Ru1–N9	170.2 (2)	C1–Ru1–C29	170.5(2)
N4–Ru1–N6	176.2(2)	C15–Ru1–N10	169.0(2)	C15–Ru1–N12	172.7(2)
N5–Ru1–N8	174.1(2)	N4–Ru1–N8	177.2(2)	N4–Ru1–N8	176.7(2)
C1–Ru1–N5	86.3(2)	C1–Ru1–N4	78.1(2)	C1–Ru1–C15	81.8(2)
N5–Ru1–N7	97.5(2)	C15–Ru1–N8	78.5(2)	C1–Ru1–N8	99.7(2)
N8–Ru1–C1	98.9(2)	N9–Ru1–N10	77.3(2)	C1–Ru1–N12	99.8(2)
N4–Ru1–N5	98.5(2)	C1–Ru1–N8	101.4(2)	C1–Ru1–N4	77.4(2)
N4–Ru1–N7	95.1(2)	N8–Ru1–N9	85.8(2)	N4–Ru1–C15	100.0(2)
N4–Ru1–N8	85.3(2)	N9–Ru1–N4	95.0(2)	C15–Ru1–N8	78.1(2)
N6–Ru1–N7	87.9(2)	N10–Ru1–N4	88.4(2)	N8–Ru1–N12	94.6(2)
N6–Ru1–C1	99.4(2)	N10–Ru1–C1	95.3(2)	N12–Ru1–N4	87.3(2)
N6–Ru1–N8	97.8(2)	N10–Ru1–N8	101.4(2)	C29–Ru1–N4	93.5(2)
N4–Ru1–C1	77.7(2)	C15–Ru1–C1	94.2(2)	C29–Ru1–C15	102.3 (2)
N6–Ru1–N5	78.6(2)	C15–Ru1–N4	98.8(2)	C29–Ru1–N8	78.1(2)
N8–Ru1–N7	77.7(2)	C15–Ru1–N9	93.8(2)	C29–Ru1–N12	77.2(2)
Ru1–C1–C2	114.8(4)	Ru1–C1–C2	114.5(4)	Ru1–C1–C2	114.1(3)
C1–C2–C4	119.5(5)	C1–C2–C4	119.9(5)	C1–C2–C4	119.5(4)
C2–C4–N4	109.8(5)	C2–C4–N4	109.1(5)	C2–C4–N4	110.0(4)
Ru1–N4–C4	118.1(3)	Ru1–N4–C4	118.2(4)	Ru1–N4–C4	118.7(3)
C1–C2–N3	108.0(5)	C1–C2–N3	108.1(5)	C1–C2–N3	109.0(4)
C2–N3–N2	112.1(4)	C2–N3–N2	112.1(5)	C2–N3–N2	111.9(4)
N3–N2–N1	103.3(4)	N3–N2–N1	103.5(4)	N3–N2–N1	103.0(3)
N1–C1–C2	101.5(4)	N1–C1–C2	101.9(4)	N1–C1–C2	100.9(4)
N4–C4–C5	121.4(5)	N4–C4–C5	121.2(6)	N4–C4–C5	122.6(4)
C4–C5–C6	119.8(6)	C4–C5–C6	120.1(6)	C4–C5–C6	119.5(5)
C5–C6–C7	118.7(6)	C5–C6–C7	119.0(6)	C5–C6–C7	118.6(5)
C6–C7–C8	119.6(6)	C6–C7–C8	118.8(7)	C6–C7–C8	119.5(4)
C7–C8–N4	122.2(6)	C7–C8–N4	124.2(6)	C7–C8–N4	122.8(5)
Ru1–N5–C19	115.6(3)	Ru1–C15–C16	114.6(4)	Ru1–C15–C16	115.4(3)
N5–C19–C20	114.5(4)	C15–C16–C18	119.6(5)	C15–C16–C18	119.2(4)
C19–C20–N6	114.9(4)	C16–C18–N8	110.1(5)	C16–C18–N8	108.8(4)
C20–N6–Ru1	116.1(3)	C18–N8–Ru1	117.1(4)	C18–N8–Ru1	118.2(3)
Ru1–N7–C29	115.5(3)	Ru1–N9–C33	115.9(4)	Ru1–C29–C30	114.9(3)
N7–C29–C30	115.0(5)	N9–C33–C34	116.5(5)	C29–C30–C32	120.0(4)
C29–C30–N8	115.1(5)	C33–C34–N10	114.6(5)	C30–C32–N12	110.5(4)
C30–N8–Ru1	116.5(3)	C34–N10–Ru1	115.7(4)	C32–N12–Ru1	117.3(3)

Table S2. Crystallographic data for **$[RuL_1](PF_6)_2$**, **$[RuL_2](PF_6)_2$** and **$[RuL_3](PF_6)_2$**.

	**$[RuL_1](PF_6)_2$**	**$[RuL_2](PF_6)_2$**	**$[RuL_3](PF_6)_2$**
Chemical formula	$C_{34}H_{28}F_{12}N_8P_2Ru$	$C_{38}H_{32}F_{12}N_{10}$ P_2Ru	$C_{48}H_{48}F_{12}N_{12}O_2P_2Ru$
M_r	939.65	1019.74	1215.99
Crystal system	Monoclinic	Monoclinic	Monoclinic
Space group	$P2(1)/c$	$P2(1)/c$	$P2(1)/c$
a (Å)	20.0489(12)	11.4148 (14)	20.8964(11)
b (Å)	13.2818(7)	29.627 (3)	13.6387(8)
c (Å)	14.1975(7)	13.5033 (13)	18.6342(9)
[illegible] (°)	90	90	90
β (°)	98.187(4)	103.737 (6)	98.509(2)
γ (°)	90	90	90
V ($Å^3$)	3742.1(4)	4435.9 (8)	5252.3(5)
Z	4	4	4
Densitiy (g · cm^{-3})	1.668	1.527	1.538
F(000)	1880	2048	2472
Radiation Type	CuK[illegible]	CuK_α	MoK[illegible]
μ (mm^{-1})	5.098	4.37	0.454
Crystal size	0.218 x 0.125 x 0.025	0.35 × 0.12 × 0.10	0.32 x 0.28 x 0.08
Meas. Refl.	31668	41866	48263
Indep. Refl.	6290	7220	9638
Obsvd. [$I > 2\sigma(I)$] refl.	4792	6609	7657
R_{int}	0.0718	0.058	0.0553
R [$F^2 > 2\sigma(F^2)$], wR(F^2), S	0.0520, 0.1346, 1.029	0.069, 0.143, 1.17	0.0609, 0.1558, 1.028
$\Delta\rho_{max}$, $\Delta\rho_{min}$ (e · $Å^{-3}$)	1.014, −0.714	0.96, −0.52	1.184, −0.979
**Deposit number**	**2075647**	**2220128**	**2075649**

4. Cyclic Voltammetry and Differential Pulse Voltammetry

4.10 Cyclic Voltammetry and Differential Pulse Voltammetry of [RuL$_1$](PF$_6$)$_2$

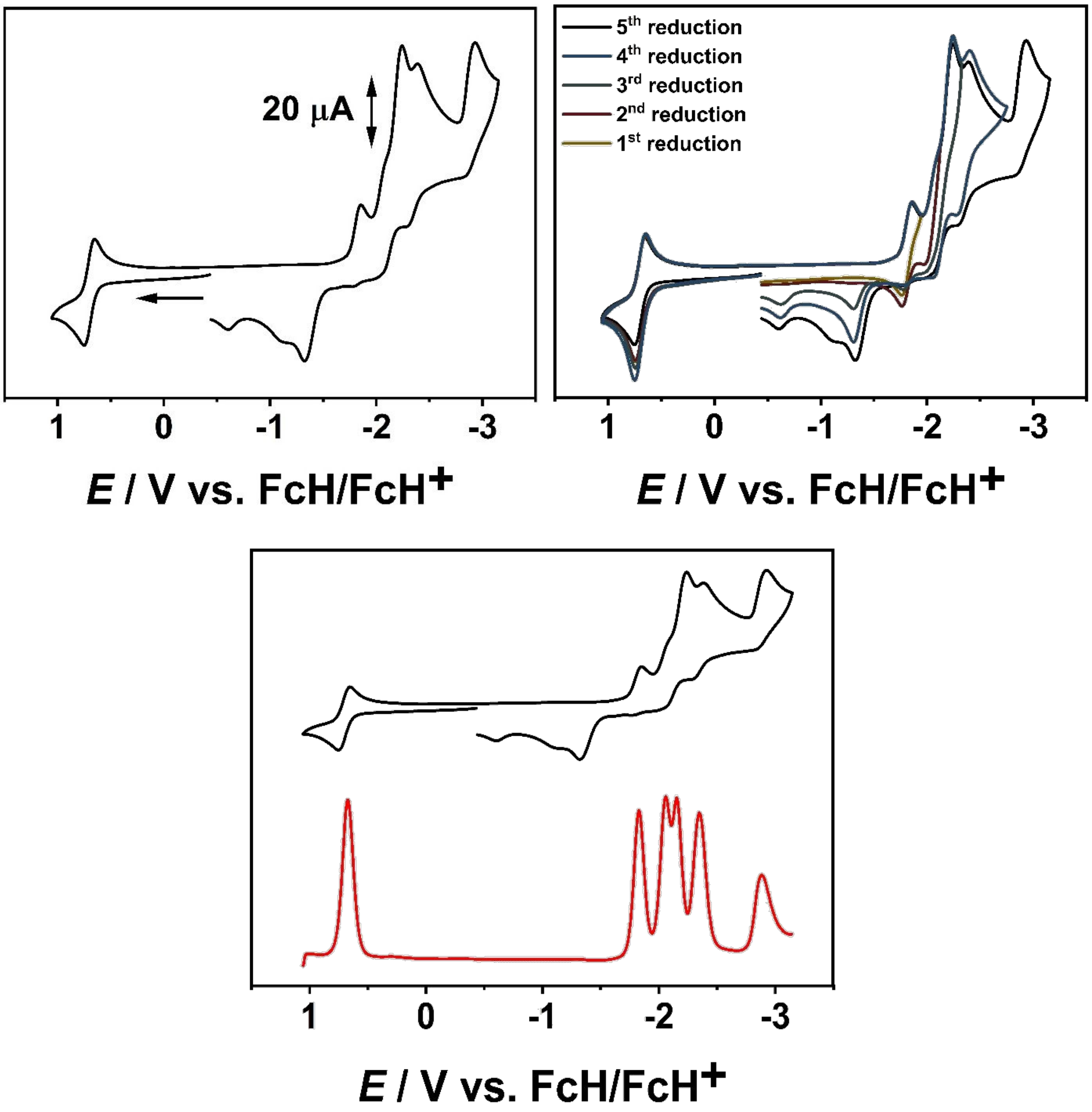

Figure S13. Cyclic voltammograms of **[RuL$_1$](PF$_6$)$_2$** in CH_3CN and 0.1 M Bu_4NPF_6 with a scan rate of 100 mV/s (top). Differential pulse voltammogramm of **[RuL$_1$](PF$_6$)$_2$** in CH_3CN and 0.1 M Bu_4NPF_6 with a scan rate of 20 mV/s (bottom).

4.20 Cyclic Voltammetry and Differential Pulse Voltammetry of $[RuL_2](PF_6)_2$

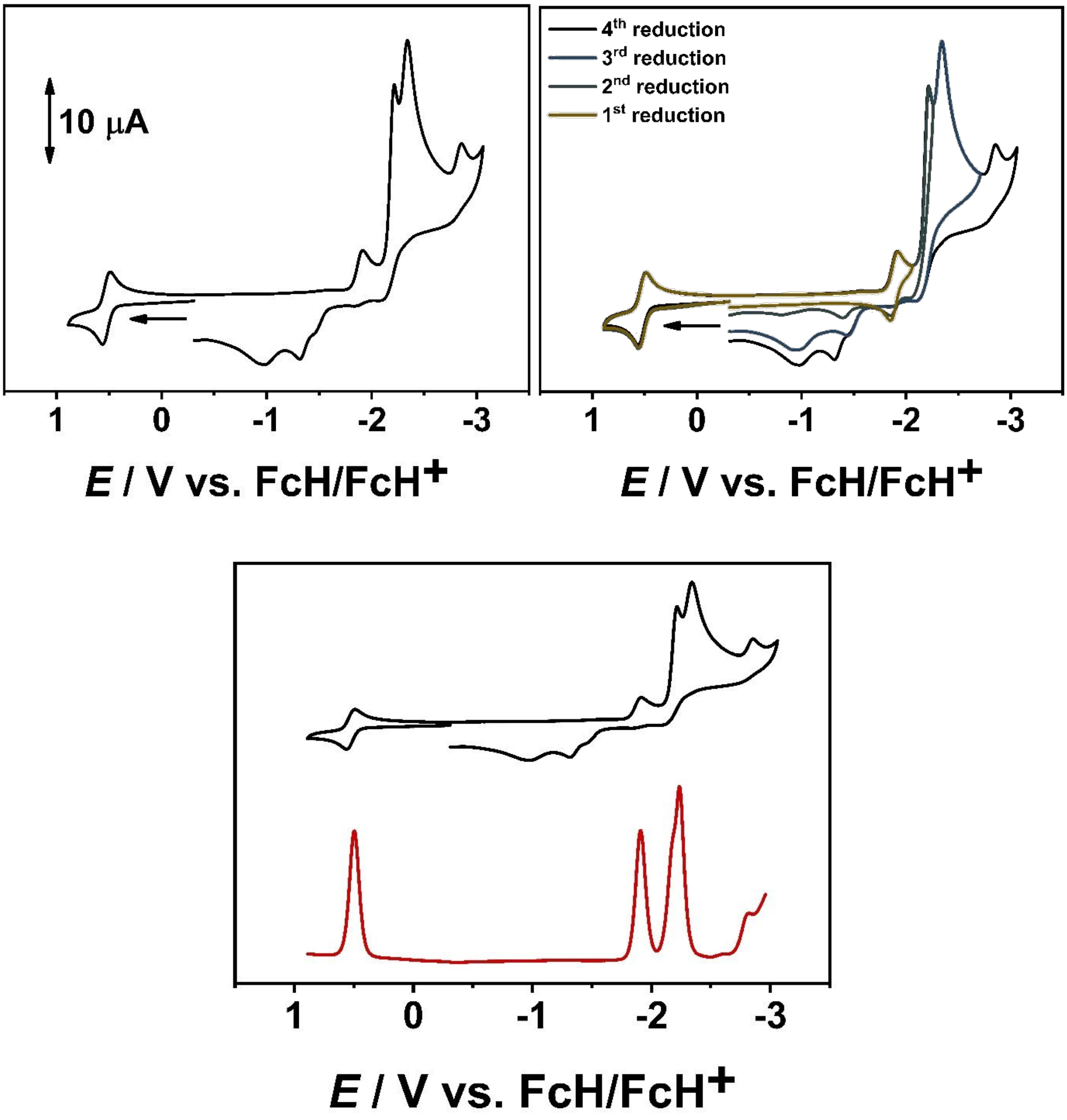

Figure S14. Cyclic voltammograms of $\mathbf{[RuL_2](PF_6)_2}$ in CH_3CN and 0.1 M Bu_4NPF_6 with a scan rate of 100 mV/s (top). Differential pulse voltammogramm of $\mathbf{[RuL_2](PF_6)_2}$ in CH_3CN and 0.1 M Bu_4NPF_6 with a scan rate of 20 mV/s (bottom).

4.30 Cyclic Voltammetry and Differential Pulse Voltammetry of [RuL$_3$](PF$_6$)$_2$

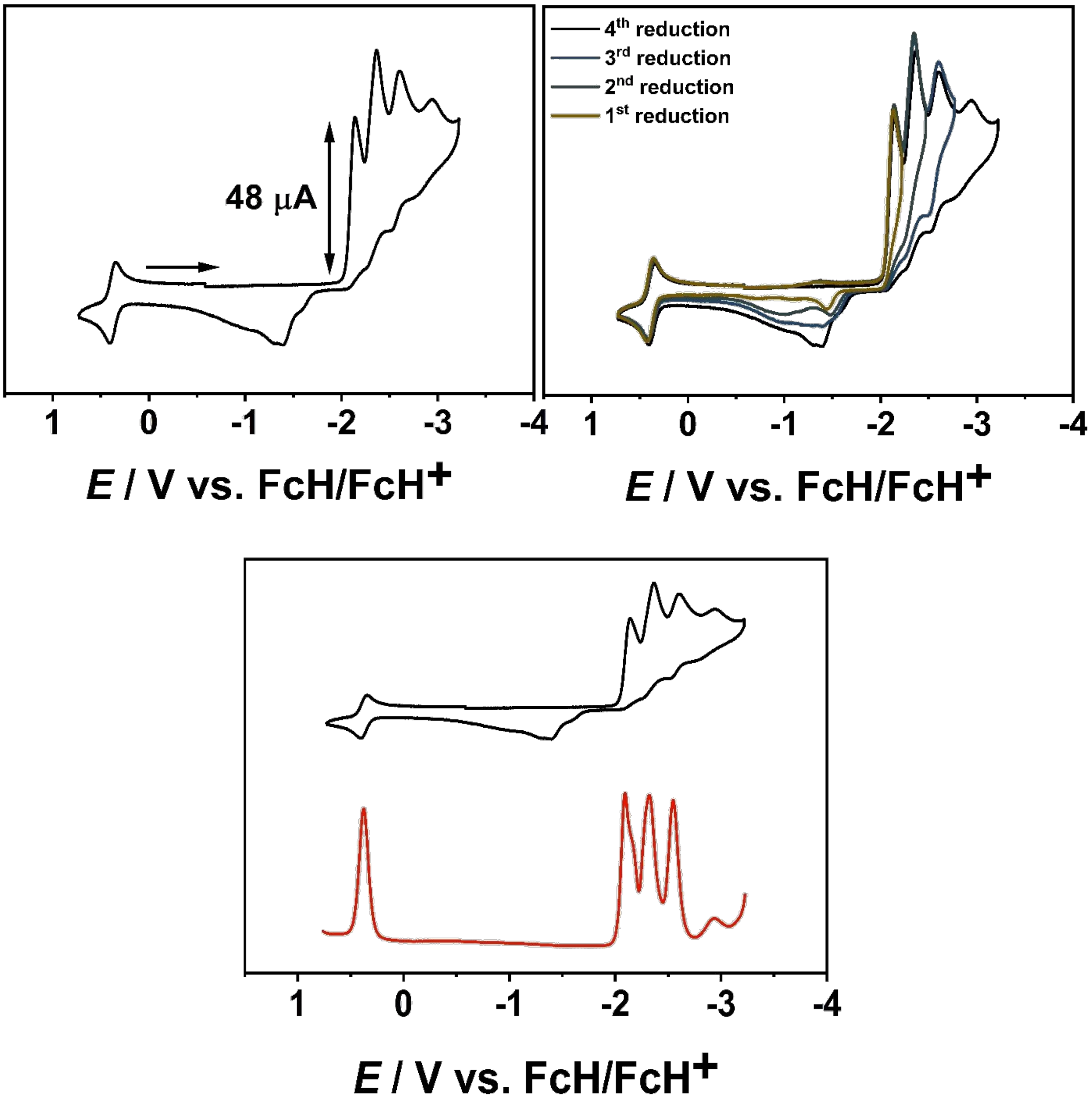

Figure S15. Cyclic voltammograms of **[RuL$_3$](PF$_6$)$_2$** in CH_3CN and 0.1 M Bu_4NPF_6 with a scan rate of 100 mV/s (top). Differential pulse voltammogramm of **[RuL$_3$](PF$_6$)$_2$** in CH_3CN and 0.1 M Bu_4NPF_6 with a scan rate of 20 mV/s (bottom).

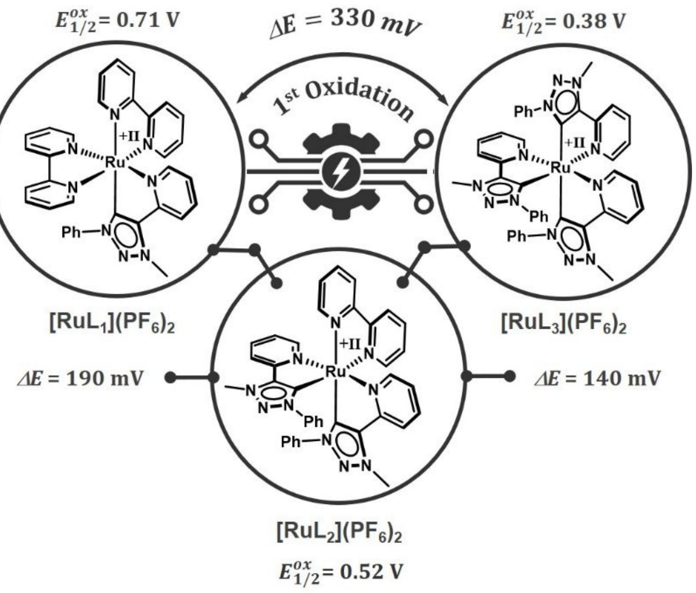

Scheme S3. Electrochemical correlation between the oxidation potentials of **[RuL$_1$](PF$_6$)$_2$**, **[RuL$_2$](PF$_6$)$_2$** and **[RuL$_3$](PF$_6$)$_2$** with respect to **L**.

Table S3. Redox potentials of **[RuL$_1$](PF$_6$)$_2$**, **[RuL$_2$](PF$_6$)$_2$**, **[RuL$_3$](PF$_6$)$_2$**, and **[Ru(bpy)$_3$](Cl)$_2$**[19] in CH_3CN and 0.1 M NBu_4PF_6 at 100 mV/s.

	$E^{ox}_{1/2}$ (ΔE)/ V	$E^{red1}_{1/2}$(ΔE)/ V	E^{red2}_{p}/ V	E^{red3}_{p}/ V	E^{red4}_{p}/ V	E^{red5}_{p}/ V
**[RuL$_1$]$^{2+}$**	0.71 (0.11)	−1.81 (0.09)	−2.02	−2.11	−2.34	−2.89
**[RuL$_2$]$^{2+}$**	0.52 (0.10)	−1.89 (0.08)	−2.22	−2.34	−2.86	-
**[RuL$_3$]$^{2+}$**	0.38 (0.08)	−2.14^a	−2.31	−2.56	−2.85	-
**[Ru(bpy)$_3$]$^{2+}$**	0.89	−1.73	−1.92	−2.16	-	-

^{a} $E^{red1}_{1/2} = E^{red1}_{p}$

[19] H. C. Zhao, B.-L. Fu. D. Schweinfurth, J. P. Harney, B. Sarkar, M.-K. Tsai, J. Rochford, *Eur. J. Inorg. Chem*. **2013**, 4410.

5. EPR-Spectroelectrochemistry and Spin Density Calculations of [RuL$_1$](PF$_6$)$_2$, [RuL$_2$](PF$_6$)$_2$ and [RuL$_3$](PF$_6$)$_2$

5.10 EPR-Spectroelectrochemistry and Spin Density Calculations of $[RuL_1](PF_6)_2$

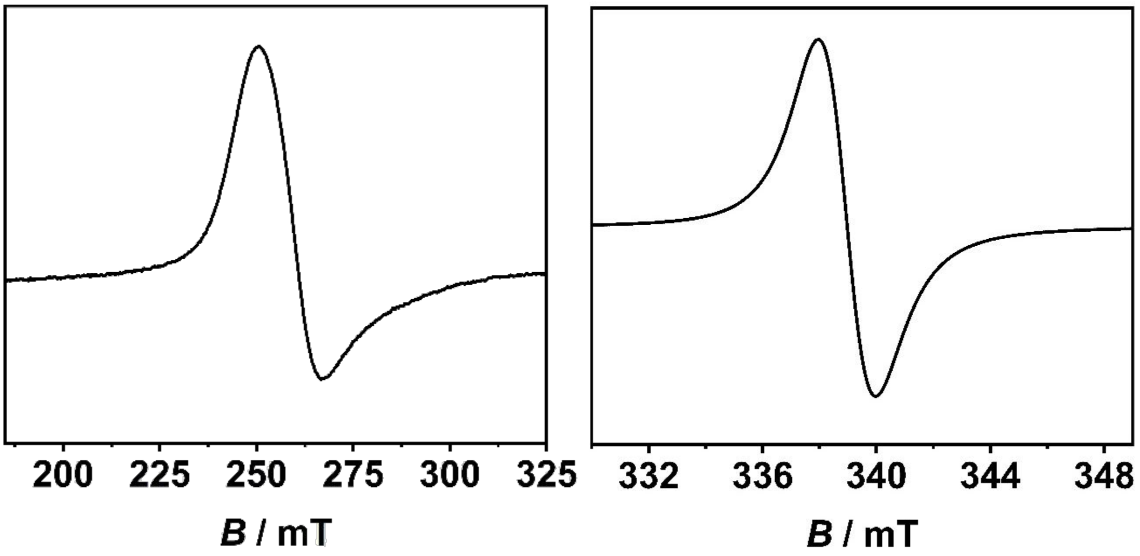

Figure S17. EPR-spectrum of $\mathbf{[RuL_1]^{3+}}$ (left, $g = 2.5953$ at -175°C) and $\mathbf{[RuL_1]^{+}}$ (right, $g = 1.9961$ at 20 °C) in CH_3CN/0.1 M Bu_4NPF_6.

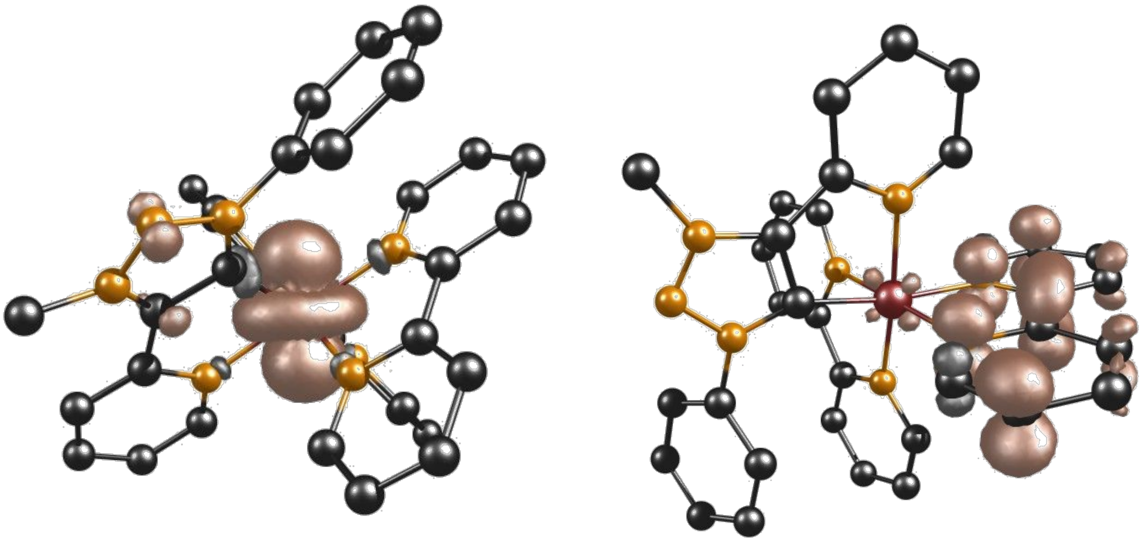

Figure S18. Spin density plot of complex $\mathbf{[RuL_1]^{3+}}$ (left) and $\mathbf{[RuL_1]^{+}}$ (right) (iso value = 0.003).

5.20 EPR-Spectroelectrochemistry and Spin Density Calculations of [RuL$_2$](PF$_6$)$_2$

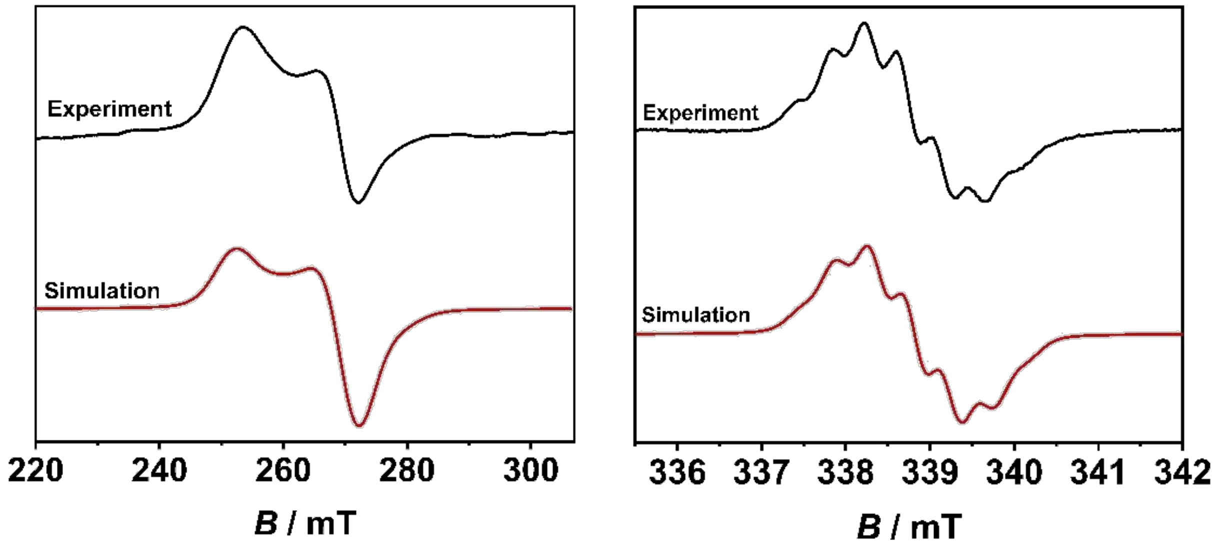

Figure S19. EPR-spectrum of **[RuL$_2$]$^{3+}$** (left, $\boldsymbol{g}_{\perp} = \mathbf{2.5036}$ and $\boldsymbol{g}_{||} = \mathbf{2.6850}$ at $-$175°C) and **[RuL$_2$]$^{+}$** (right, $\boldsymbol{g} =$ 1.9980 at 20 °C) in CH$_3$CN/0.1 M Bu$_4$NPF$_6$.

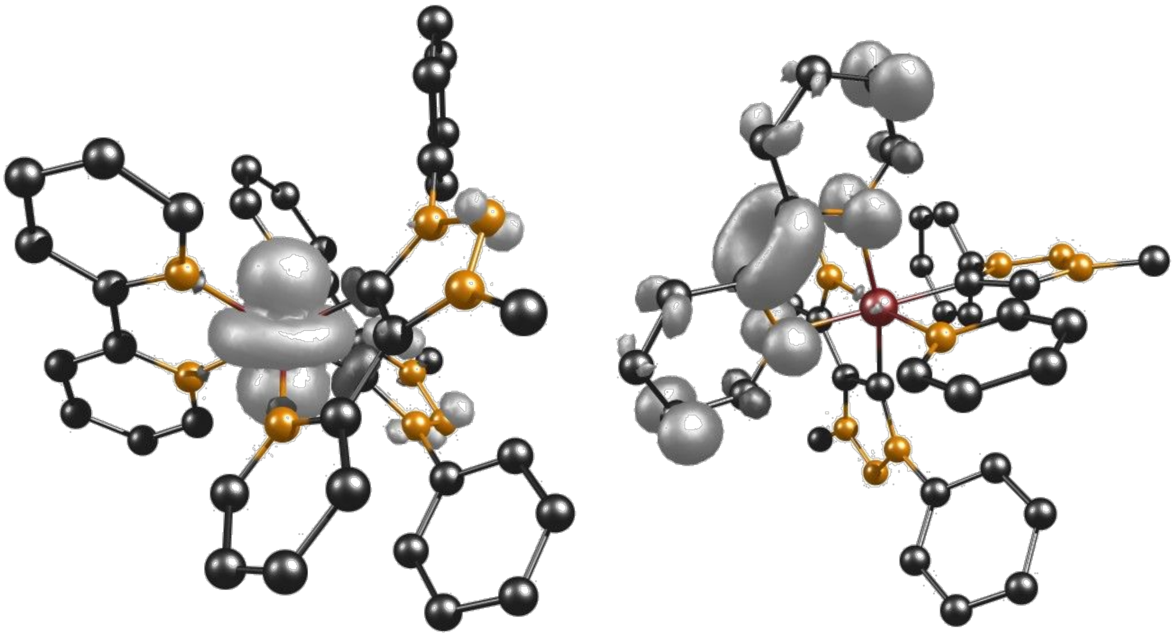

Figure S20. Spin density plot of complex **[RuL$_2$]$^{3+}$** (left) and **[RuL$_2$]$^{+}$** (right) (iso value = 0.003).

Table S4. EPR simulation data of **$[RuL_2]^{3+}$** and **$[RuL_2]^{+}$**.

**$[RuL_2]^{3+}$**	
$g_{\|\|}$	2.5036
$g_{\perp}$	2.6850
**ARu**	[120 2]
**Isotropic line broadening**	[0.70 1.49]
**Anisotropic line broadening**	[220 230]
**$[RuL_2]^{+}$**	
**g**	1.9980
**AN1**	12.73
**AH1**	11.13
**AH2**	11.96
**AH3**	12.00
**AH4**	8.20
**Line width for isotropic broadening / mT**	[0.340 0.010]

5.30 EPR-Spectroelectrochemistry and Spin Density Calculations of $[RuL_3](PF_6)_2$

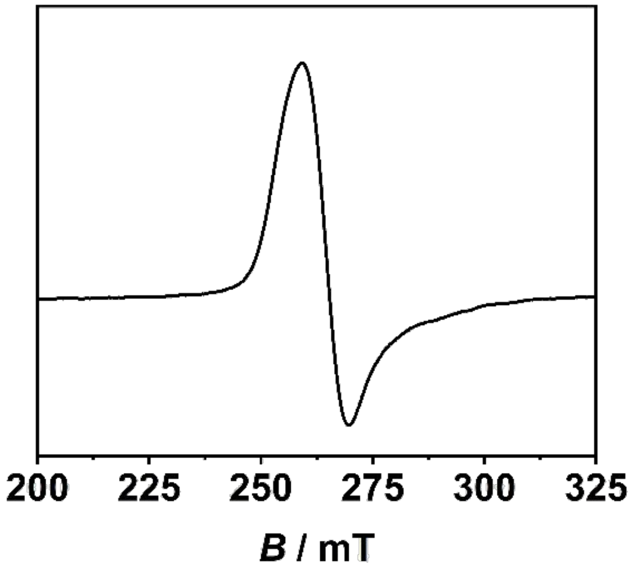

Figure S21. EPR-spectrum of $\mathbf{[RuL_3]^{3+}}$ (g = 2.5526 at – 175 °C) in CH_3CN/0.1 M Bu_4NPF_6.

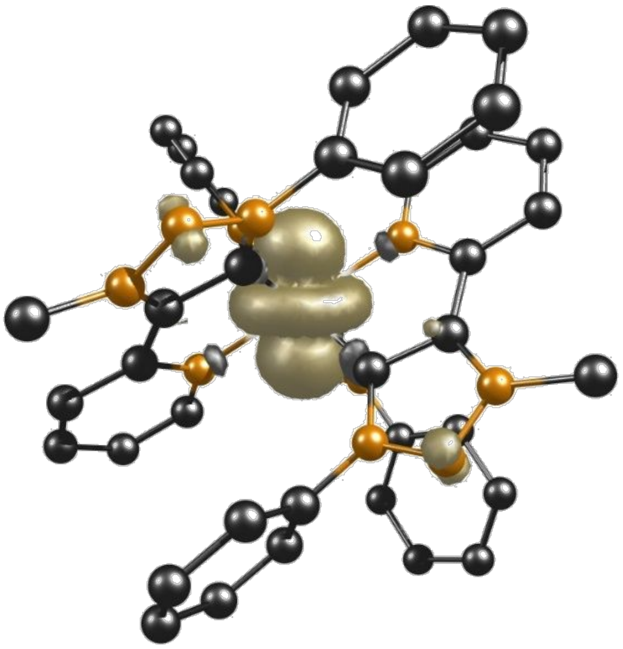

Figure S22. Spin density plot of complex $\mathbf{[RuL_3]^{3+}}$ (iso value = 0.003).

6. UV/vis/NIR-Spectroelectrochemistry of $[RuL_1](PF_6)_2$ and $[RuL_3](PF_6)_2$

6.10 UV/vis/NIR-Spectroelectrochemistry of $[RuL_1](PF_6)_2$

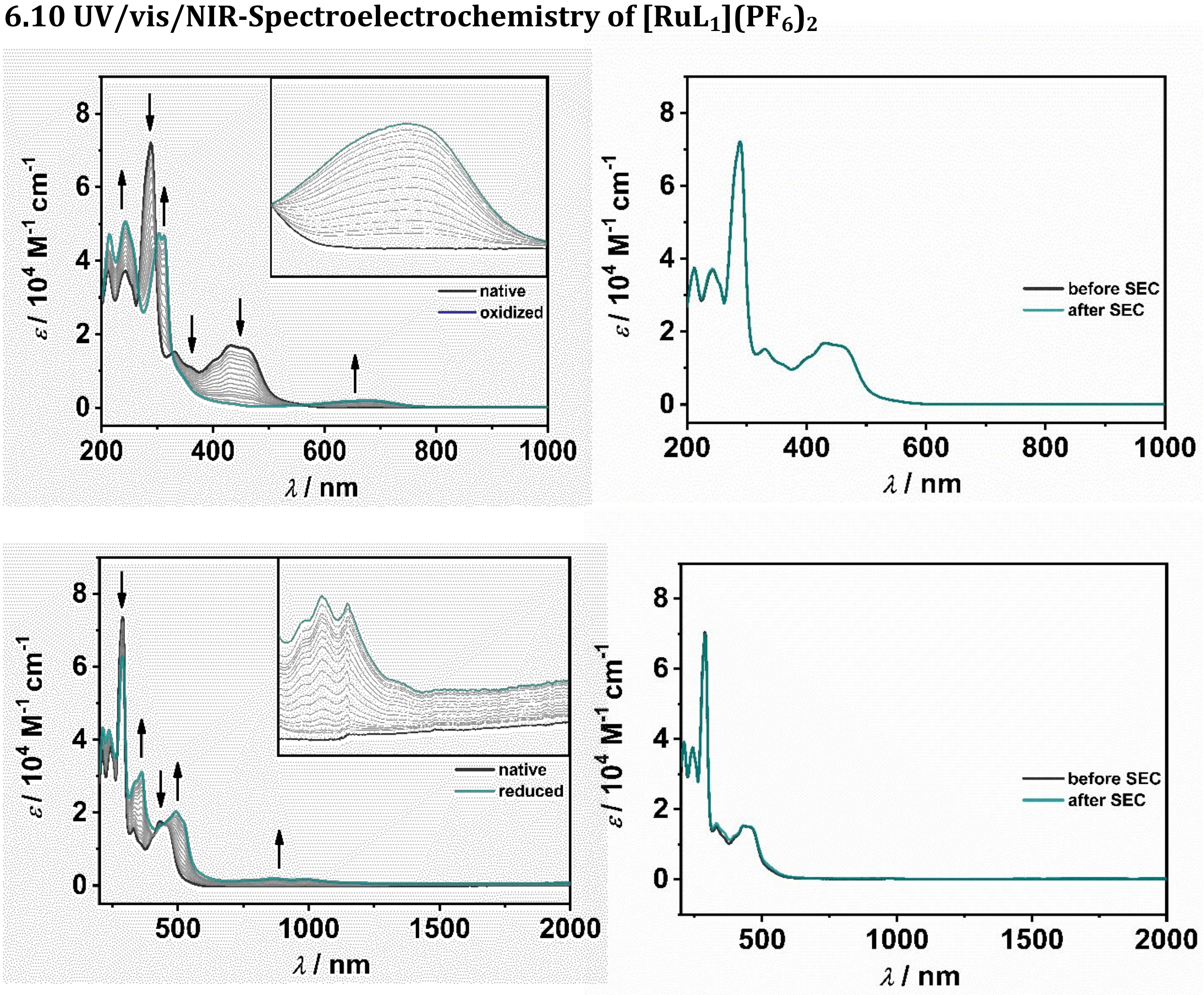

Figure S23. Changes in the UV/vis/NIR spectra of $\mathbf{[RuL_1](PF_6)_2}$ in CH_3CN/0.1 M Bu_4NPF_6 during the first oxidation (top, inset: 540-800 nm) and first reduction (bottom, inset: 550 nm-2000 nm) with a Au working electrode.

6.20 UV/vis/NIR-Spectroelectrochemistry of [RuL$_2$](PF$_6$)$_2$

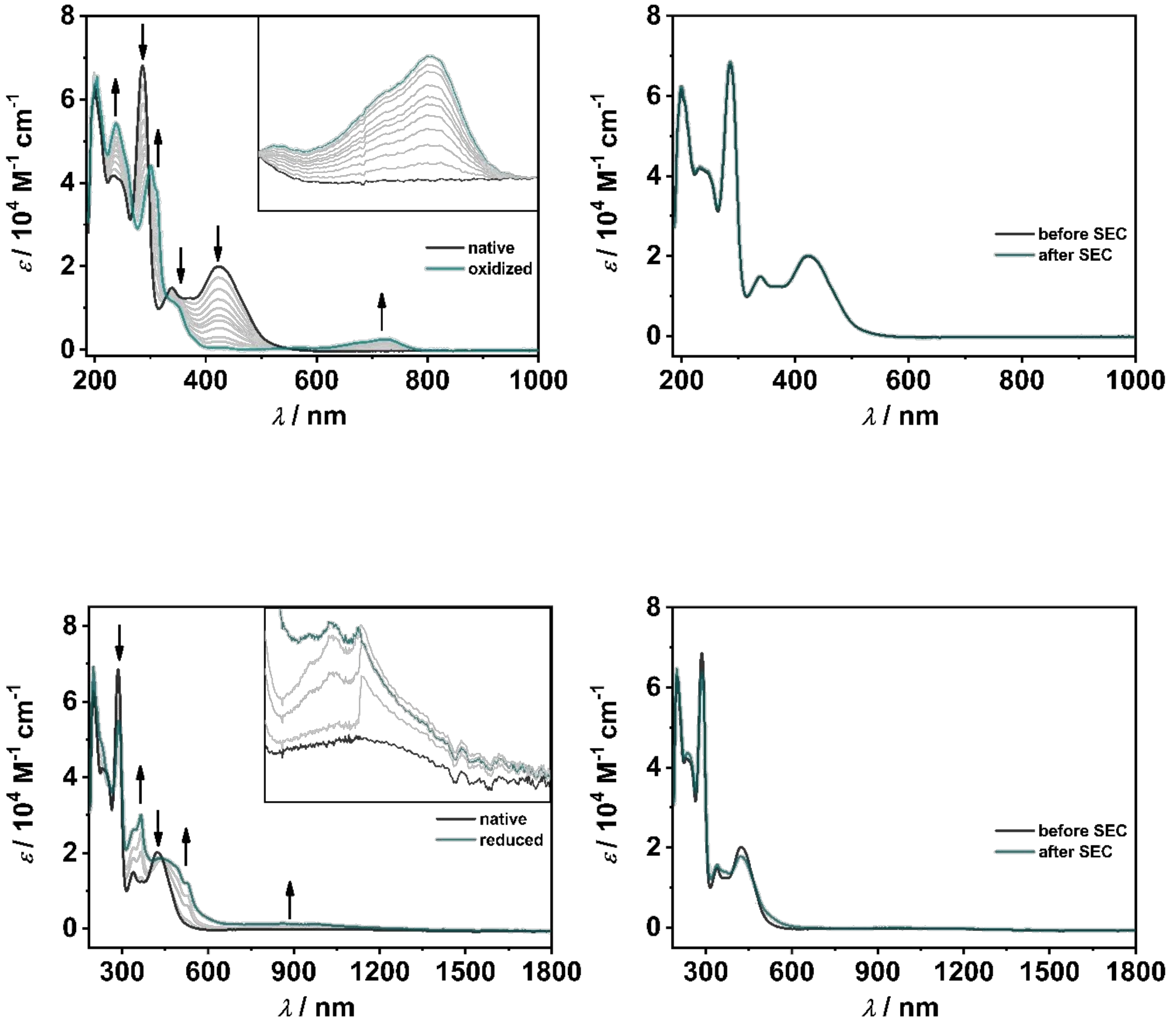

Figure S24. Changes in the UV/vis/NIR spectra of **[RuL$_2$](PF$_6$)$_2$** in CH_3CN/0.1 M Bu_4NPF_6 during the first oxidation (top, inset: 550-830 nm) and first reduction (bottom, inset: 580 nm-1800 nm) with a Au working electrode.

6.30 UV/vis/NIR-Spectroelectrochemistry of [RuL$_3$](PF$_6$)$_2$

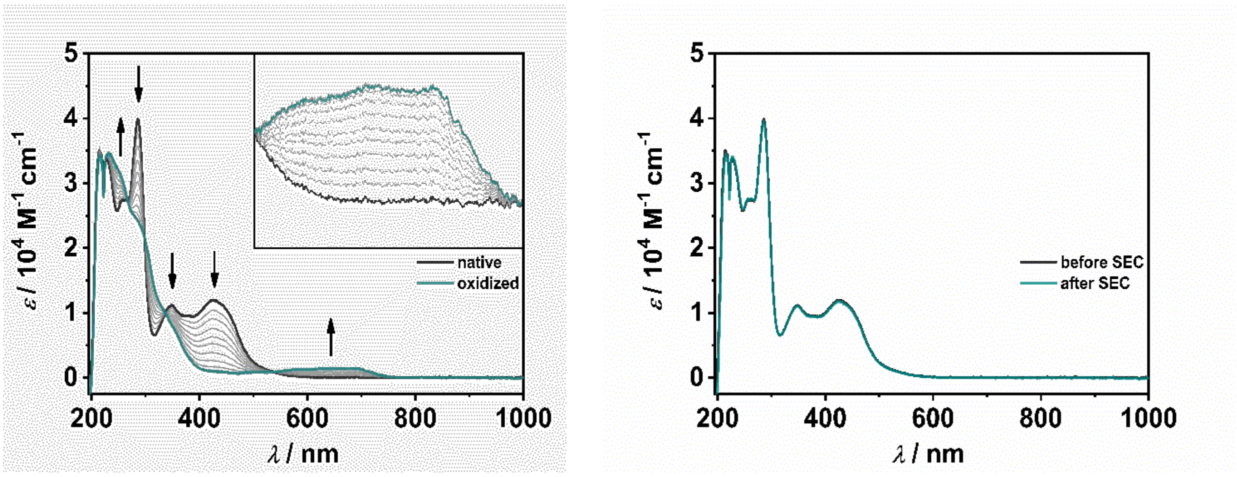

Figure S25. Changes in the UV/vis/NIR spectra of **[RuL$_3$](PF$_6$)$_2$** in CH_3CN/0.1 M Bu_4NPF_6 during the first oxidation with a Au working electrode (Inset: 520-750 nm).

7. (TD)-DFT of [RuL$_1$](PF$_6$)$_2$, [RuL$_2$](PF$_6$)$_2$ and [RuL$_3$](PF$_6$)$_2$

7.10 (TD)-DFT of [RuL$_1$](PF$_6$)$_2$

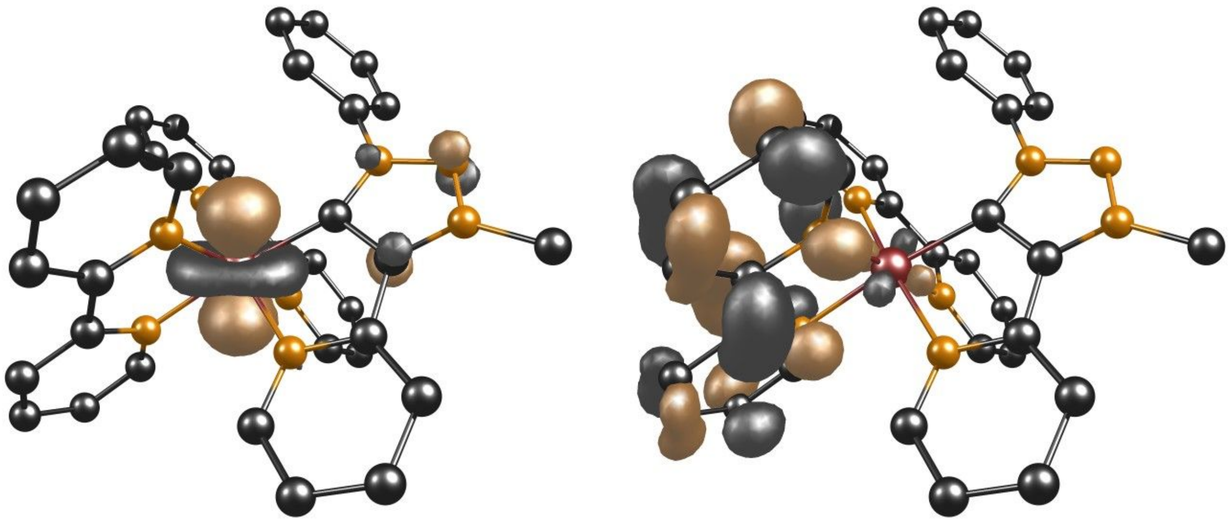

Figure S26. HOMO (left) and LUMO (right) of complex **[RuL$_1$]$^{2+}$**.

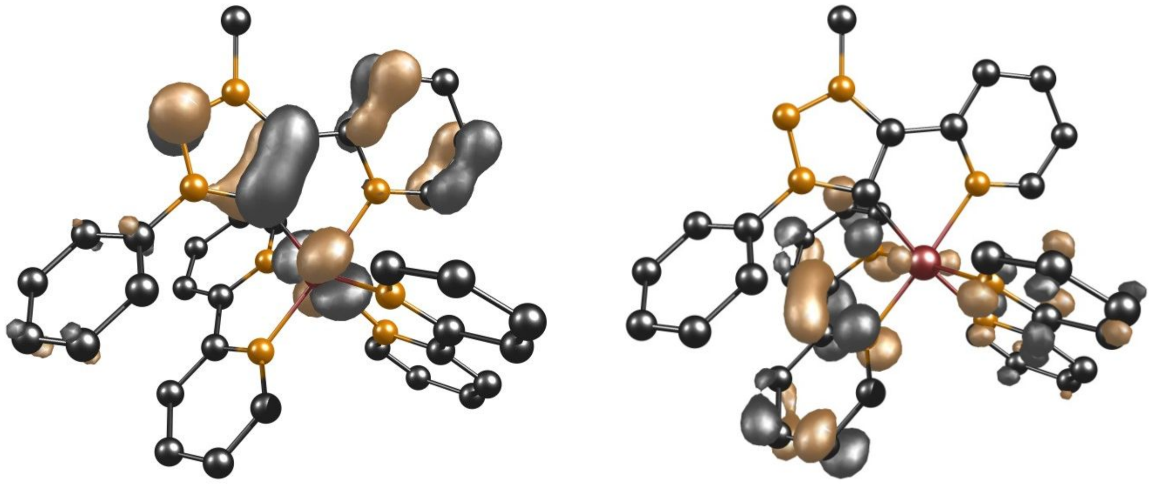

Figure S27. α-HOMO (left) and α-LUMO (right) of complex **[RuL$_1$]$^{3+}$**.

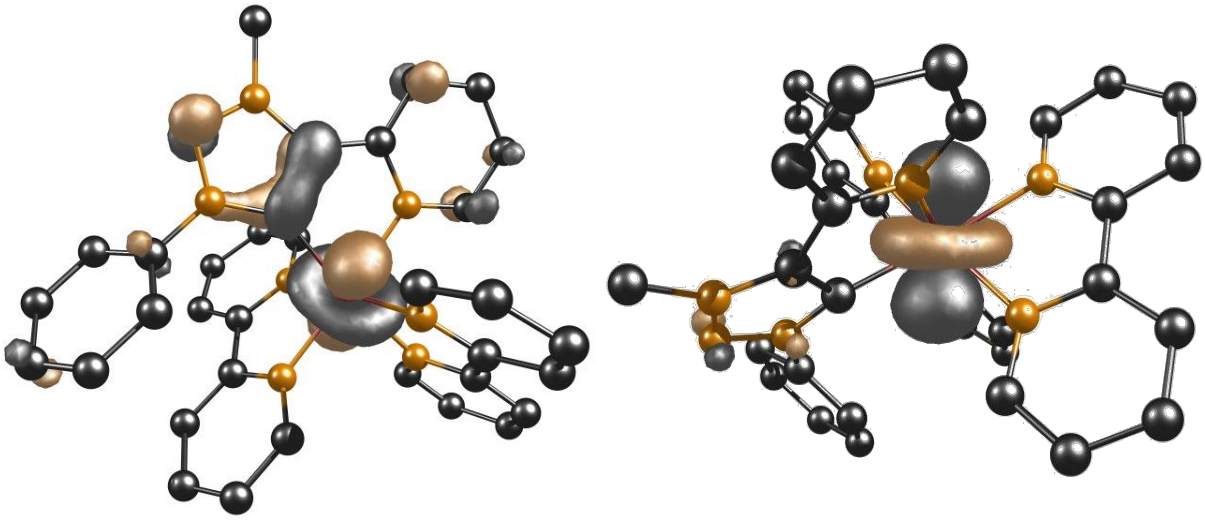

Figure S28. β-HOMO (left) and β -LUMO (right) of complex **[RuL$_1$]$^{3+}$**.

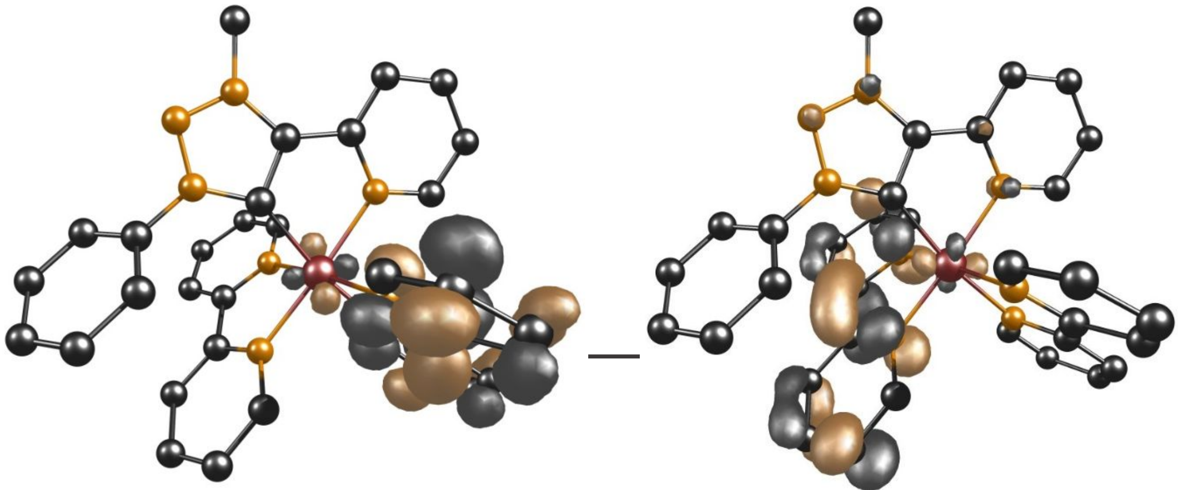

Figure S29. α-HOMO (left) and α-LUMO (right) of complex $\mathbf{[RuL_1]^{1+}}$.

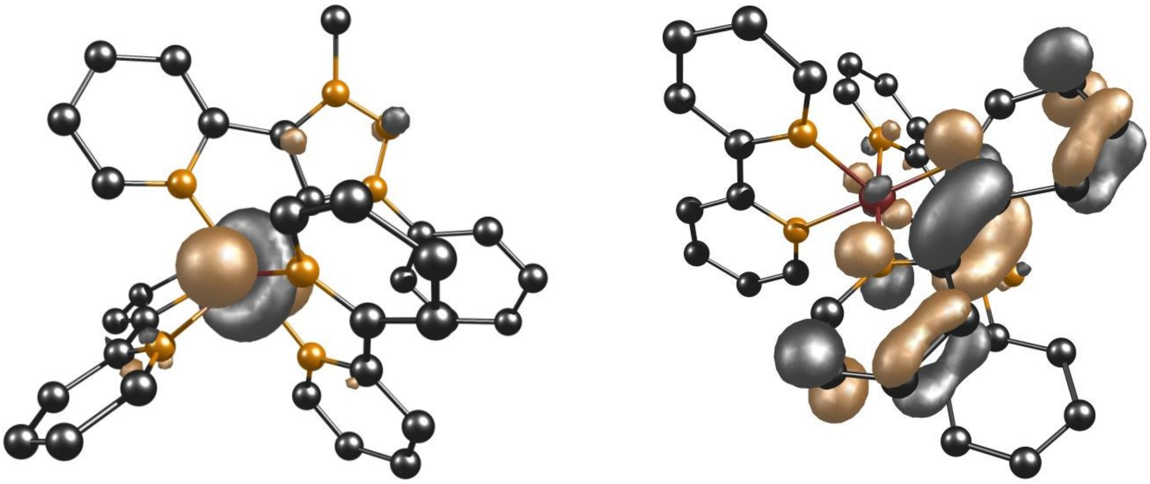

Figure S30. β-HOMO (left) and β-LUMO (right) of complex $\mathbf{[RuL_1]^{1+}}$.

Table S5. Energies of selected orbitals $\mathbf{[RuL_1](PF_6)_2}$.

MO	Energy / eV
$[\mathbf{RuL_1}]^{\mathbf{2+}}_{\mathbf{HOMO}}$	− 6.0608
$[\mathbf{RuL_1}]^{\mathbf{2+}}_{\mathbf{LUMO}}$	− 2.3630
$[\mathbf{RuL_1}]^{\mathbf{3+}}_{\boldsymbol{\alpha}-\mathbf{HOMO}}$	− 7.8724
$[\mathbf{RuL_1}]^{\mathbf{3+}}_{\boldsymbol{\alpha}-\mathbf{LUMO}}$	− 3.0122
$[\mathbf{RuL_1}]^{\mathbf{3+}}_{\boldsymbol{\beta}-\mathbf{HOMO}}$	− 7.7344
$[\mathbf{RuL_1}]^{\mathbf{3+}}_{\boldsymbol{\beta}-\mathbf{LUMO}}$	− 4.8165
$[\mathbf{RuL_1}]^{\mathbf{1+}}_{\boldsymbol{\alpha}-\mathbf{HOMO}}$	− 3.4015
$[\mathbf{RuL_1}]^{\mathbf{1+}}_{\boldsymbol{\alpha}-\mathbf{LUMO}}$	− 2.1663
$[\mathbf{RuL_1}]^{\mathbf{1+}}_{\boldsymbol{\beta}-\mathbf{HOMO}}$	− 5.5247
$[\mathbf{RuL_1}]^{\mathbf{1+}}_{\boldsymbol{\beta}-\mathbf{LUMO}}$	− 2.1636

Table S6. Selected experimental UV/vis data of **[RuL$_1$](PF$_6$)$_2$** together with selected TD-DFT calculations.

State	$\lambda_{exp}(\lambda_{DFT})$ / nm	ε / 10^4 M^{-1}cm^{-1}(*f*)	Main contributing excitation (%)
**5**	458 (452)	1.61 (0.077)	HOMO → LUMO+2 (78)
**4**	458 (447)	1.61 (0.005)	HOMO-2 → LUMO+1 (54)<br>HOMO-1 → LUMO+1 (19)
**6**	458 (421)	1.61 (0.127)	HOMO-2 → LUMO (17)<br>HOMO-2 → LUMO+1 (27)<br>HOMO-1 → LUMO+1 (50)
**8**	432 (413)	1.69 (0.068)	HOMO-2 → LUMO+2 (67)<br>HOMO-1 → LUMO+2 (23)
**7**	432 (407)	1.69 (0.065)	HOMO-1 → LUMO (78)<br>HOMO-1 → LUMO+1 (20)<br>HOMO-1 → LUMO+2 (11)
**9**	402 (381)	1.29 (0.011)	HOMO-2 → LUMO+2 (16)<br>HOMO-1 → LUMO+2 (38)<br>HOMO → LUMO+3 (29)
**10**	402 (371)	1.29 (0.055)	HOMO-1 → LUMO+2 (17)<br>HOMO → LUMO+3 (63)

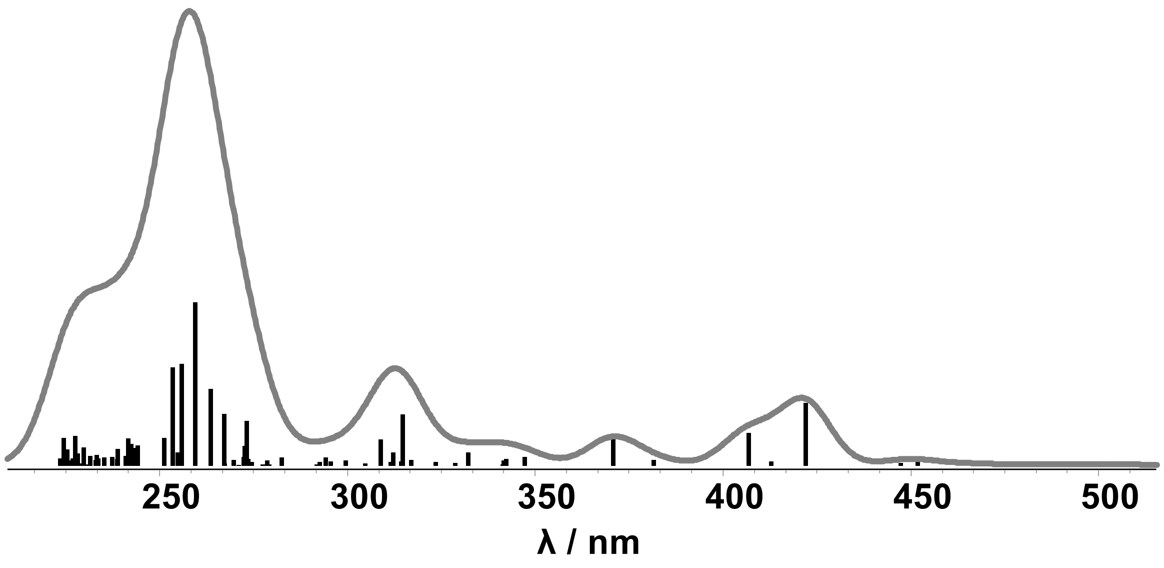

Figure S31. Calculated TD-DFT spectrum with discrete transitions of **[RuL$_1$]**$^{2+}$.

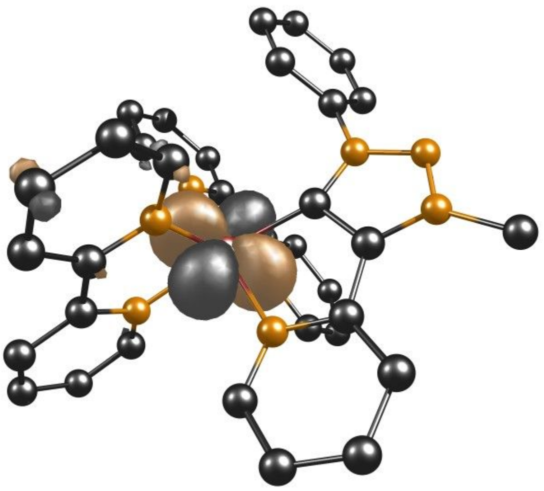

HOMO-2

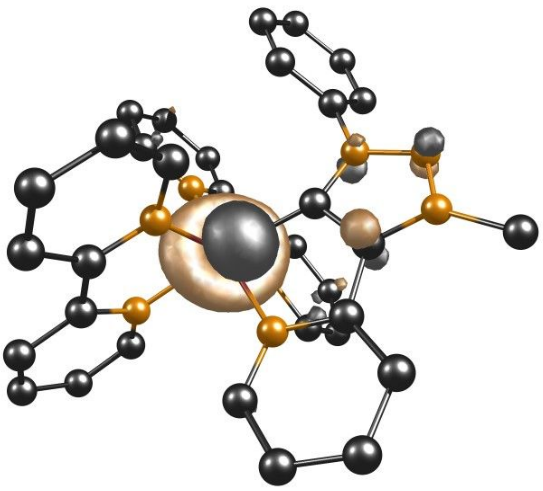

HOMO-1

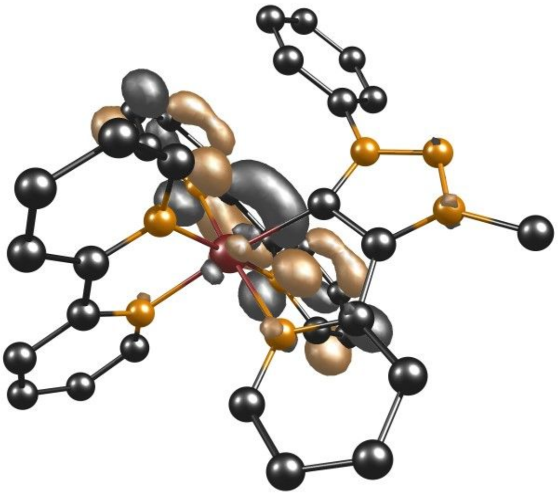

LUMO+1

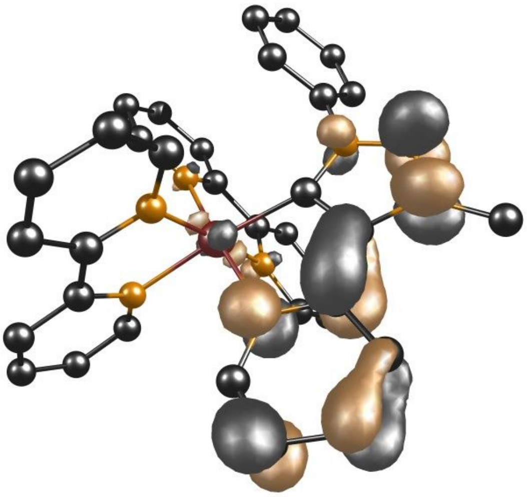

LUMO+2

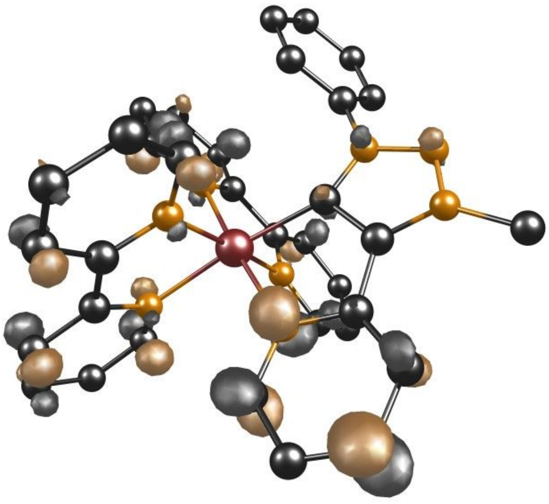

LUMO+3

Figure S32. Involved TD-DFT orbitals of complex $\mathbf{[RuL_1]}^{2+}$.

Table S7. Selected experimental UV/vis data of **[RuL$_1$]$^{3+}$** together with selected TD-DFT calculations.

State	$\lambda_{exp}(\lambda_{DFT})$ / nm	ε / 10^4 $M^{-1}cm^{-1}$(f)	Main contributing excitation (%)
**4**	562-788 (555)	0.19 (0.006)	β-HOMO-4 → β-LUMO (75)
**3**	562-788 (554)	0.19 (0.012)	β-HOMO-6 → β-LUMO (17)
			β-HOMO-2 → β-LUMO (48)
			β-HOMO-1 → β-LUMO (22)
**5**	562-788 (532)	0.19 (0.003)	β-HOMO-5 → β-LUMO (72)
			β-HOMO-1 → β-LUMO (11)

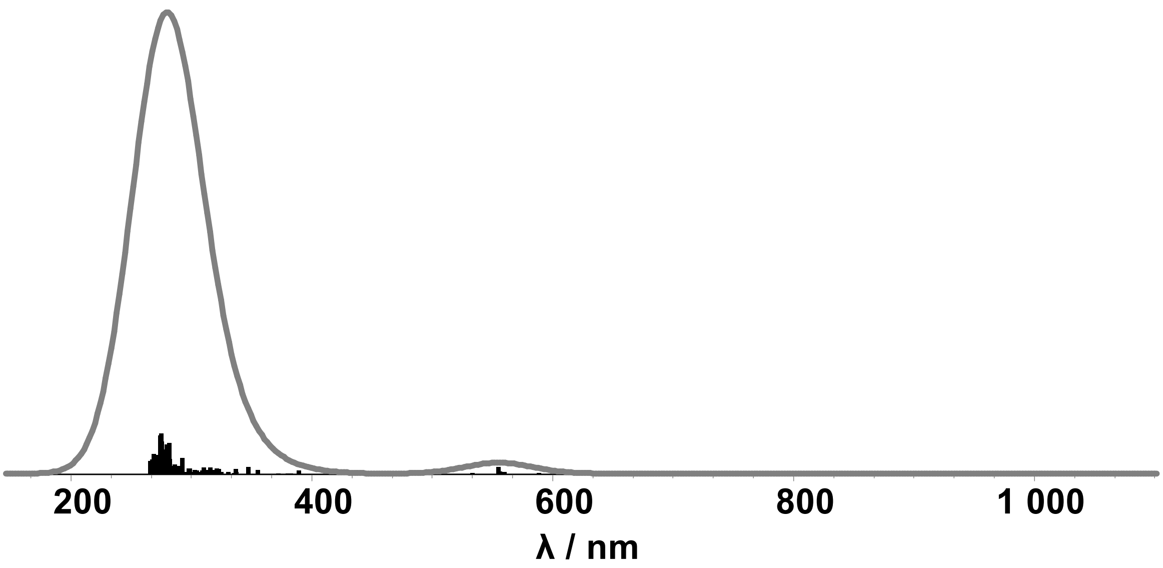

Figure S33. Calculated TD-DFT spectrum with discrete transitions of **[RuL$_1$]$^{3+}$**.

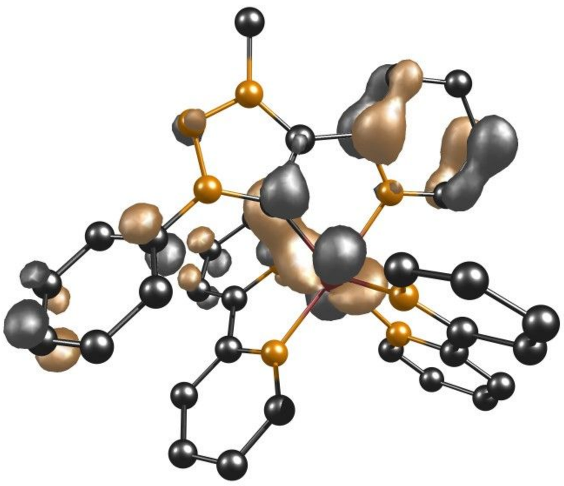

***β*-HOMO-6**

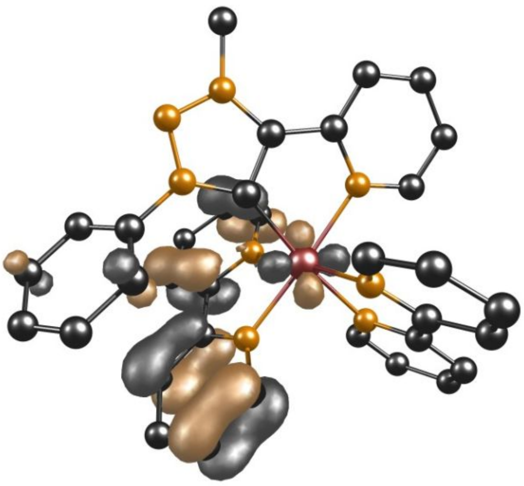

***β*-HOMO-5**

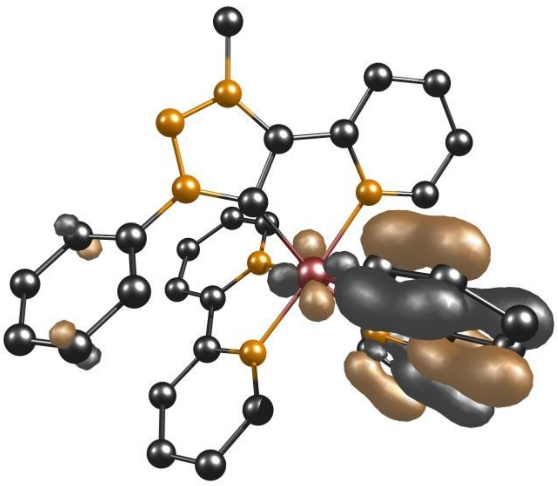

***β*-HOMO-4**

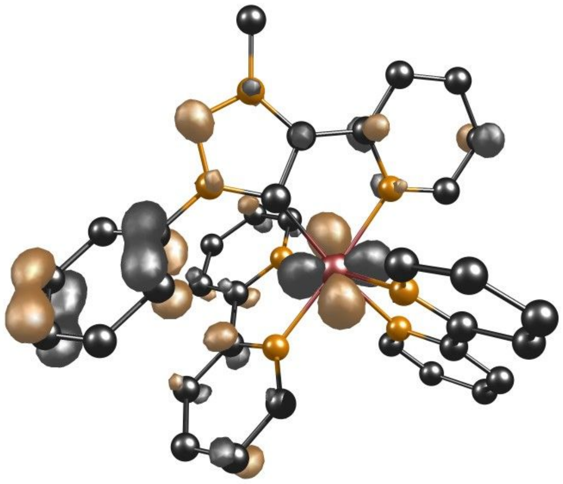

***β*-HOMO-2**

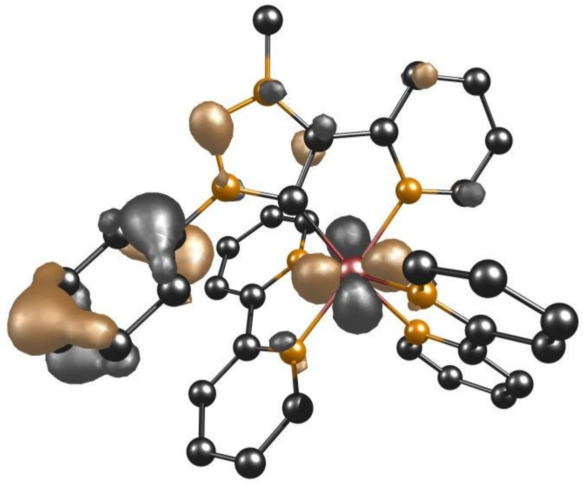

***β*-HOMO-1**

Figure S34. Involved TD-DFT orbitals of complex **$[RuL_1]^{3+}$**.

Table S8. Selected experimental UV/vis data of **$[RuL_1]^{1+}$** together with selected TD-DFT calculations.

State	$\lambda_{exp}(\lambda_{DFT})$ / nm	ε / 10^4 $M^{-1}cm^{-1}$(f)	Main contributing excitation (%)
**3**	1300-2000 (1263)	0.13 (0.001)	α-HOMO → α-LUMO+2 (83)<br>α-HOMO → α-LUMO+6 (11)
**6**	980 (947)	0.18 (0.001)	α-HOMO → α-LUMO+4 (78)<br>α-HOMO → α-LUMO+8 (11)
**5**	980 (932)	0.18 (0.002)	α-HOMO → α-LUMO+3 (49)<br>α-HOMO → α-LUMO+6 (29)
**4**	980 (930)	0.18 (0.003)	α-HOMO → α-LUMO+3 (38)<br>α-HOMO → α-LUMO+4 (12)<br>α-HOMO → α-LUMO+6 (37)
**8**	862 (861)	0.19 (0.003)	α-HOMO → α-LUMO+5 (84)<br>α-HOMO → α-LUMO+6 (12)
**7**	772 (738)	0.16 (0.016)	α-HOMO → α-LUMO+8 (71)
**10**	518 (588)	1.79 (0.002)	α-HOMO → α-LUMO (55)<br>β-HOMO → β-LUMO (36)
**18**	518 (586)	1.79 (0.003)	α-HOMO → α-LUMO+9 (93)
**16**	492 (516)	2.03 (0.006)	α-HOMO-1 → α-LUMO+1 (45)<br>β-HOMO → β-LUMO+1 (35)
**15**	492 (510)	2.03 (0.001)	α-HOMO-2 → α-LUMO+1 (27)<br>β-HOMO-1 → β-LUMO+1 (34)
**17**	492 (506)	2.03 (0.004)	α-HOMO-3 → α-LUMO (66)<br>α-HOMO-2 → α-LUMO (17)

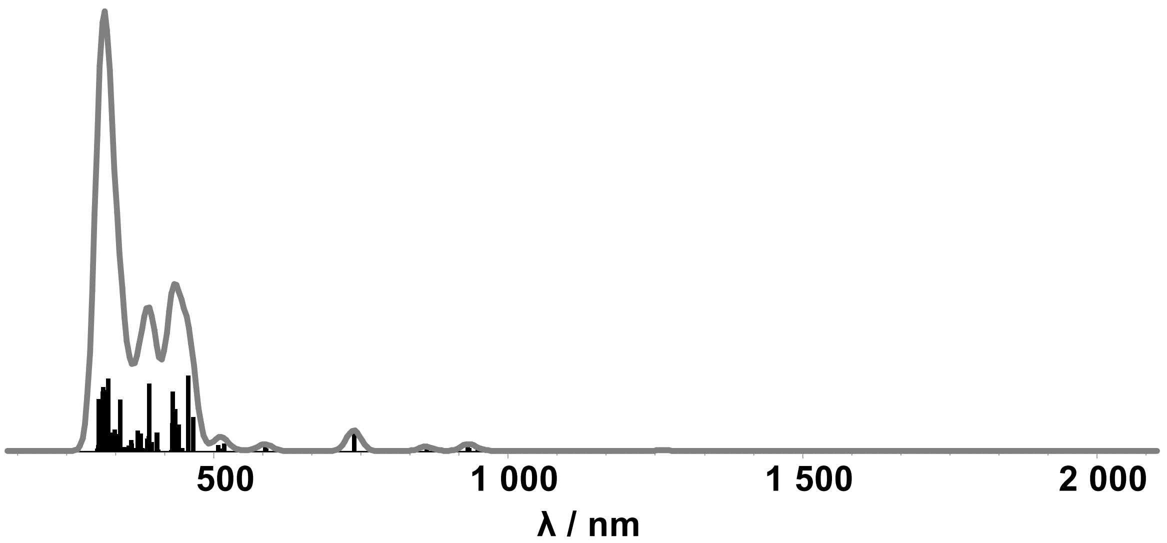

Figure S35. Calculated TD-DFT spectrum with discrete transitions of **$[RuL_1]^{1+}$**.

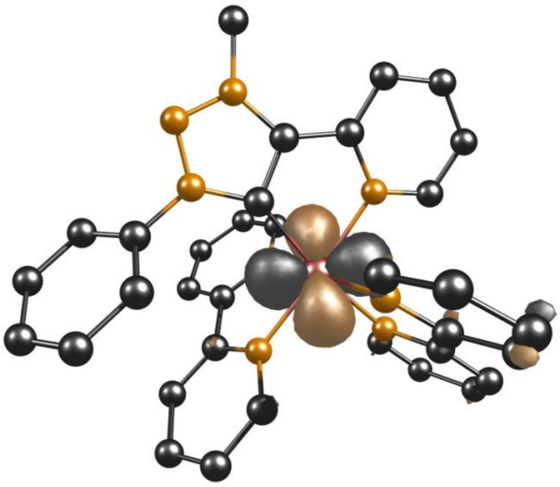

α-HOMO-3

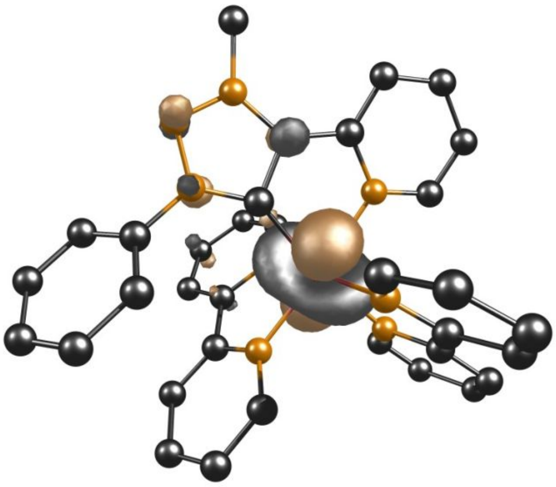

α-HOMO-2

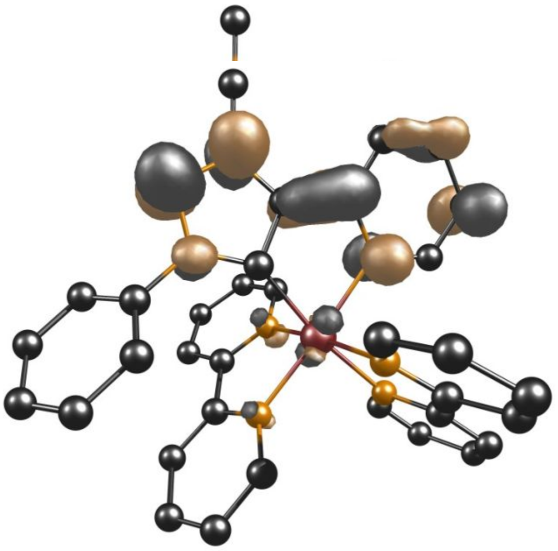

α-HOMO-1

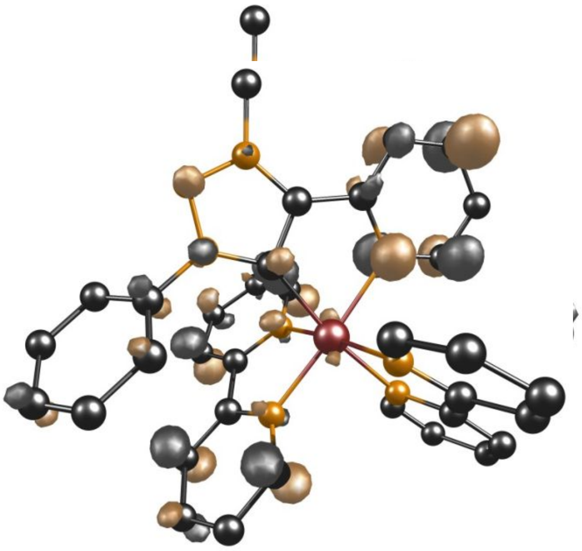

β-HOMO-1

α-LUMO+1

α-LUMO+2

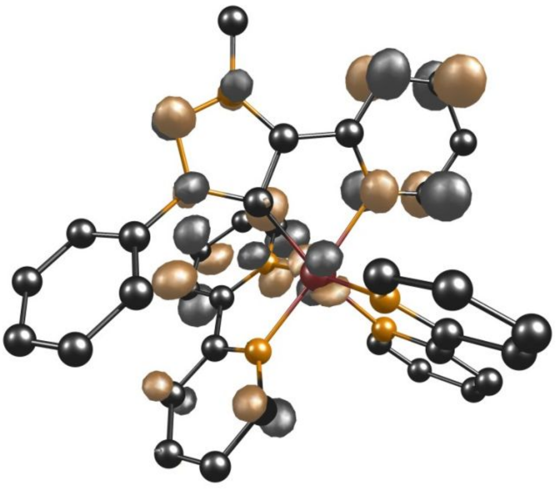

α-LUMO+3

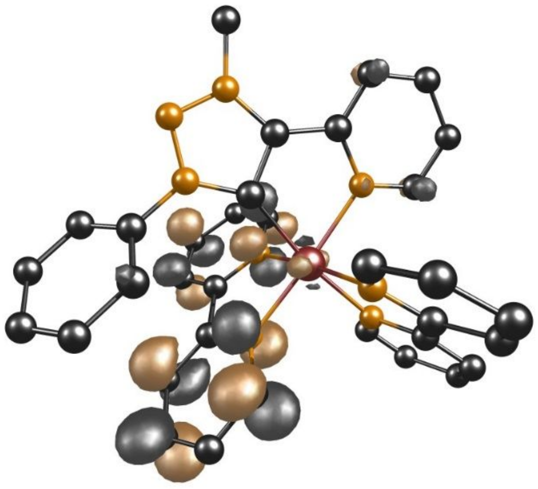

α-LUMO+4

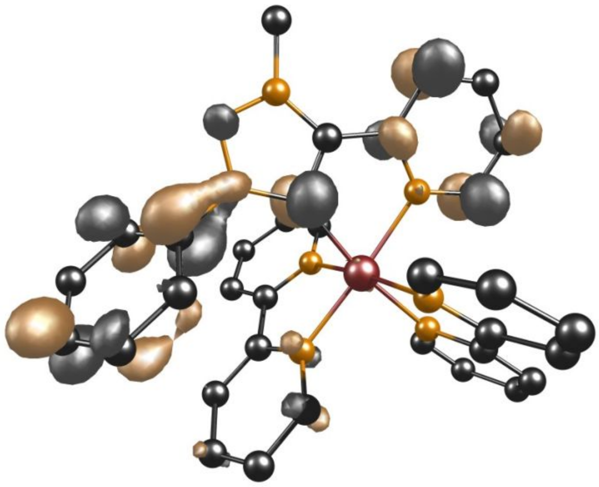

α-LUMO+5

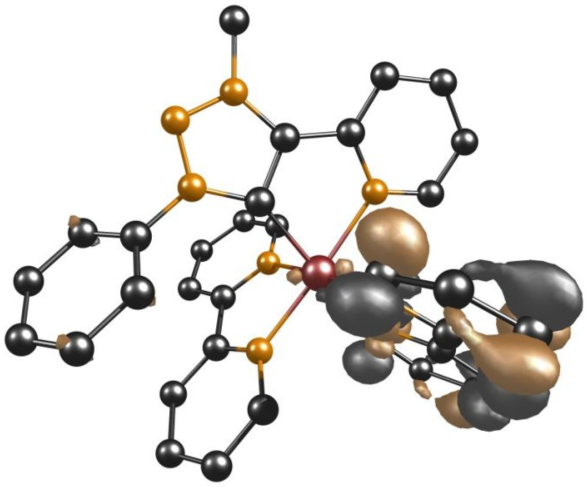

α-LUMO+6

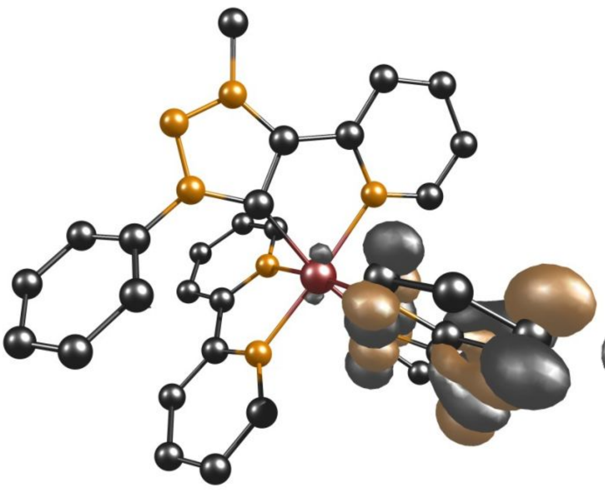

α-LUMO+8

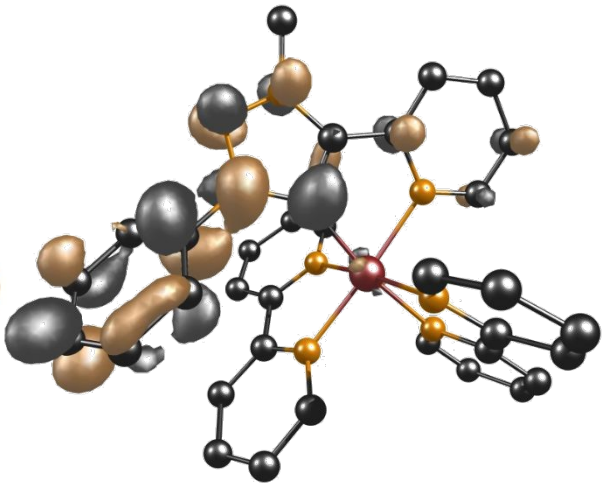

α-LUMO+9

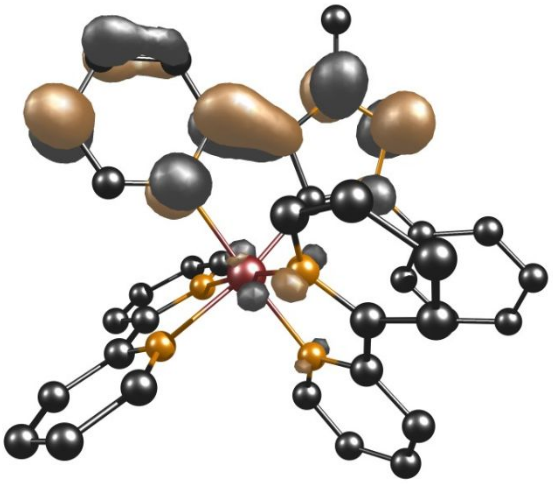

***β*-LUMO+1**

Figure S36. Involved TD-DFT orbitals of complex **$[RuL_1]^{1+}$**.

Table S9. Selected MO energies of **$[RuL_1]^{2+}$**, **$[RuL_1]^{3+}$** and **$[RuL_1]^{1+}$**.

$[RuL_1]^{2+}$		$[RuL_1]^{3+}$		$[RuL_1]^{1+}$	
**MO**	**Energy / eV**	**MO**	**Energy / eV**	**MO**	**Energy / eV**
**HOMO-2**	– 6.3418	***β*-HOMO-6**	– 8.4661	***α*-HOMO-3**	– 5.9483
**HOMO-1**	– 6.2865	***β*-HOMO-5**	– 8.1463	***α*-HOMO-2**	– 5.8002
**LUMO+1**	– 2.3089	***β*-HOMO-4**	– 8.1262	***α*-HOMO-1**	– 5.5733
**LUMO+2**	– 2.1969	***β*-HOMO-2**	– 7.9970	***β*-HOMO-1**	– 5.7429
**LUMO+3**	– 1.7661	***β*-HOMO-1**	– 7.9531	***α*-LUMO+1**	– 1.9378
				***α*-LUMO+2**	– 1.4598
				***α*-LUMO+3**	– 1.1542
				***α*-LUMO+4**	– 1.0696
				***α*-LUMO+5**	– 0.9183
				***α*-LUMO+6**	– 0.6764
				***α*-LUMO+8**	– 0.4930
				***α*-LUMO+9**	– 0.1069
				***β*-LUMO+1**	– 1.9362

Table S10. XYZ coordinates of optimized $\mathbf{[RuL_1]^{2+}}$.

	x	y	z
Ru	13.40733	6.01451	9.408
N	14.39195	4.28817	9.84511
N	12.30621	5.83832	11.17504
N	15.1361	6.7456	10.2319
N	11.65257	5.18753	8.73069
N	12.54525	7.87894	8.90973
C	14.30311	6.42194	7.65019
C	13.8754	3.05862	9.77937
C	15.67295	4.43744	10.24939
C	12.75023	6.12466	12.40007
C	11.02364	5.45377	11.01308
C	16.10532	5.82312	10.42199
C	15.40934	8.03163	10.46622
C	11.39739	4.84711	7.46335
C	10.6505	5.11389	9.63734
C	12.96087	8.46248	7.75471
C	11.58984	8.49059	9.61181
N	15.34025	6.04664	6.84992
C	13.98766	7.67183	7.12598
H	12.84382	2.98779	9.46204
C	14.60831	1.92861	10.08098
C	16.47358	3.34093	10.5279
H	13.78904	6.41891	12.47616
C	11.93825	6.07393	13.51629
C	10.15031	5.39231	12.09008
C	17.38881	6.19528	10.7919
H	14.59352	8.72789	10.32495
C	16.66119	8.46469	10.85696
H	12.22913	4.91619	6.77598
C	10.14694	4.44886	7.03496
C	9.36929	4.73008	9.26839
C	12.4091	9.64598	7.29197
H	11.28484	8.01348	10.53268
C	11.00191	9.67796	9.21471
N	15.67529	6.94469	5.92994
C	16.09169	4.83705	6.90298
N	14.84648	7.92823	6.11438
H	14.13715	0.95664	10.01785
C	15.9372	2.06989	10.44607
H	17.50219	3.48126	10.82984
H	12.34677	6.32519	14.48633
C	10.61026	5.71088	13.35567
H	9.12212	5.09069	11.94392
C	17.67625	7.53136	11.00031
H	18.1575	5.44526	10.91704
H	16.83072	9.51877	11.03361
H	9.99759	4.18881	5.99528
C	9.10813	4.40175	7.95148
H	8.58127	4.69261	10.0077
H	12.74376	10.07667	6.35941
C	11.41561	10.26382	8.03085
H	10.23389	10.1238	9.83249
C	15.41667	3.62909	6.85007
C	17.47377	4.89413	6.99588
C	14.95968	9.11334	5.28767
H	16.54504	1.20357	10.6741
H	9.94028	5.67125	14.20534
H	18.67469	7.8386	11.28492
H	8.11191	4.10696	7.64648
H	10.97163	11.18659	7.6785
C	16.14297	2.45076	6.90918
H	14.33816	3.6141	6.75695
H	17.97531	5.85332	7.03695
C	18.19046	3.70779	7.0514
H	15.82596	8.98741	4.64495
H	15.09263	9.98071	5.93303
H	14.05965	9.2236	4.68492
H	15.62262	1.50151	6.87187
C	17.52722	2.4887	7.01027
H	19.27016	3.73859	7.13608
H	18.09078	1.56461	7.06169

Table S11. XYZ coordinates of optimized **$[RuL_1]^{3+}$**.

	x	y	z
Ru	13.39453	6.01095	9.38774
N	14.36079	4.28207	9.86636
N	12.32499	5.80168	11.17307
N	15.14516	6.74443	10.17581
N	11.6174	5.21601	8.71942
N	12.51631	7.85927	8.87785
C	14.31454	6.43519	7.62422
C	13.81663	3.06497	9.80834
C	15.64864	4.42796	10.24942
C	12.80597	6.06146	12.38926
C	11.0357	5.43344	11.02153
C	16.09514	5.80987	10.39821
C	15.42808	8.03658	10.35547
C	11.35028	4.91939	7.44469
C	10.63839	5.11895	9.64816
C	12.95195	8.45655	7.73742
C	11.53927	8.44372	9.57458
N	15.37429	6.0681	6.86158
C	13.98958	7.67735	7.11248
H	12.77954	3.00615	9.50773
C	14.54276	1.92916	10.10081
C	16.43493	3.32468	10.52683
H	13.84993	6.3391	12.45108
C	12.01258	6.00163	13.51753
C	10.18553	5.36342	12.11221
C	17.37866	6.17601	10.7656
H	14.62605	8.74123	10.18158
C	16.68175	8.46069	10.74637
H	12.16698	5.01237	6.74197
C	10.09431	4.52384	7.03192
C	9.35484	4.74251	9.29087
C	12.4051	9.64277	7.28492
H	11.22072	7.95276	10.48308
C	10.95493	9.63201	9.18138
N	15.70518	6.96578	5.95271
C	16.12929	4.85905	6.93759
N	14.86793	7.94535	6.11865
H	14.06212	0.96209	10.0443
C	15.87579	2.06148	10.45208
H	17.46744	3.45034	10.82038
H	12.44089	6.23156	14.48373
C	10.67884	5.65697	13.37286
H	9.15147	5.07558	11.98354
C	17.67842	7.51578	10.9319
H	18.13606	5.42111	10.92372
H	16.86626	9.51692	10.88887
H	9.92603	4.29004	5.9894
C	9.07641	4.44859	7.96855
H	8.57919	4.68094	10.04105
H	12.75582	10.09172	6.36731
C	11.39216	10.23825	8.0162
H	10.17121	10.06362	9.78872
C	15.4552	3.65122	6.87141
C	17.50672	4.92727	7.0706
C	14.98075	9.13197	5.29105
H	16.47548	1.18877	10.67685
H	10.02524	5.61083	14.23463
H	18.67819	7.81824	11.21605
H	8.07613	4.15732	7.67429
H	10.94944	11.16403	7.67102
C	16.18383	2.47635	6.95886
H	14.38104	3.62994	6.7331
H	18.00308	5.88878	7.11906
C	18.22339	3.74286	7.15679
H	15.85242	9.00787	4.65569
H	15.10498	9.99821	5.93932
H	14.08303	9.2319	4.68354
H	15.6689	1.52506	6.90674
C	17.56428	2.52193	7.10323
H	19.29956	3.77729	7.27465
H	18.12898	1.60034	7.17733

Table S12. XYZ coordinates of optimized **[RuL₁]¹⁺**.

	x	y	z
Ru	13.41841	6.01449	9.38787
N	14.38117	4.28828	9.83599
N	12.3278	5.86426	11.16451
N	15.15149	6.73237	10.21715
N	11.6744	5.15901	8.71036
N	12.54378	7.86565	8.90074
C	14.30556	6.43594	7.63327
C	13.8454	3.06558	9.77735
C	15.65924	4.41978	10.25754
C	12.75339	6.16948	12.39098
C	11.02313	5.45033	10.98099
C	16.10873	5.80019	10.42311
C	15.44292	8.01679	10.44301
C	11.40426	4.79425	7.45327
C	10.66574	5.09521	9.65909
C	12.96055	8.47219	7.75715
C	11.57406	8.45381	9.60424
N	15.34934	6.07644	6.83056
C	13.99015	7.69363	7.11947
H	12.81596	3.01691	9.44871
C	14.55685	1.9272	10.0979
C	16.43865	3.31281	10.55769
H	13.79181	6.47428	12.4643
C	11.95343	6.13403	13.51149
C	10.14962	5.41996	12.09622
C	17.39545	6.15896	10.79814
H	14.63635	8.72164	10.28975
C	16.69733	8.43761	10.83829
H	12.23472	4.86343	6.76065
C	10.16726	4.36998	7.02523
C	9.36982	4.6833	9.26394
C	12.40569	9.66295	7.3135
H	11.26744	7.94505	10.50744
C	10.98111	9.6449	9.22416
N	15.68921	6.98776	5.92261
C	16.10056	4.86869	6.88057
N	14.85393	7.96567	6.11658
H	14.07138	0.96177	10.03884
C	15.8841	2.04967	10.47947
H	17.46497	3.44087	10.87375
H	12.3583	6.40443	14.47754
C	10.60369	5.75793	13.34101
H	9.11911	5.11704	11.95808
C	17.70023	7.49242	10.99709
H	18.15283	5.399	10.93294
H	16.88002	9.49109	11.00645
H	10.01767	4.08947	5.99124
C	9.11739	4.3302	7.96865
H	8.57618	4.65727	10.00009
H	12.74497	10.11337	6.39174
C	11.40216	10.25924	8.05601
H	10.2012	10.07205	9.84068
C	15.42131	3.66154	6.86365
C	17.48467	4.92071	6.94648
C	14.96877	9.1619	5.30726
H	16.47454	1.17573	10.72407
H	9.93006	5.7326	14.19008
H	18.70082	7.78918	11.28556
H	8.1225	4.02016	7.66986
H	10.95511	11.18626	7.71857
C	16.14409	2.48184	6.93398
H	14.34097	3.65128	6.79598
H	17.99057	5.87834	6.9608
C	18.19817	3.7328	7.0125
H	15.84002	9.04876	4.66873
H	15.09373	10.02156	5.96456
H	14.07289	9.27889	4.69944
H	15.61955	1.53417	6.92687
C	17.53013	2.51566	7.01047
H	19.27946	3.76103	7.07631
H	18.0911	1.59057	7.07272

7.20 (TD)-DFT of [RuL$_2$](PF$_6$)$_2$

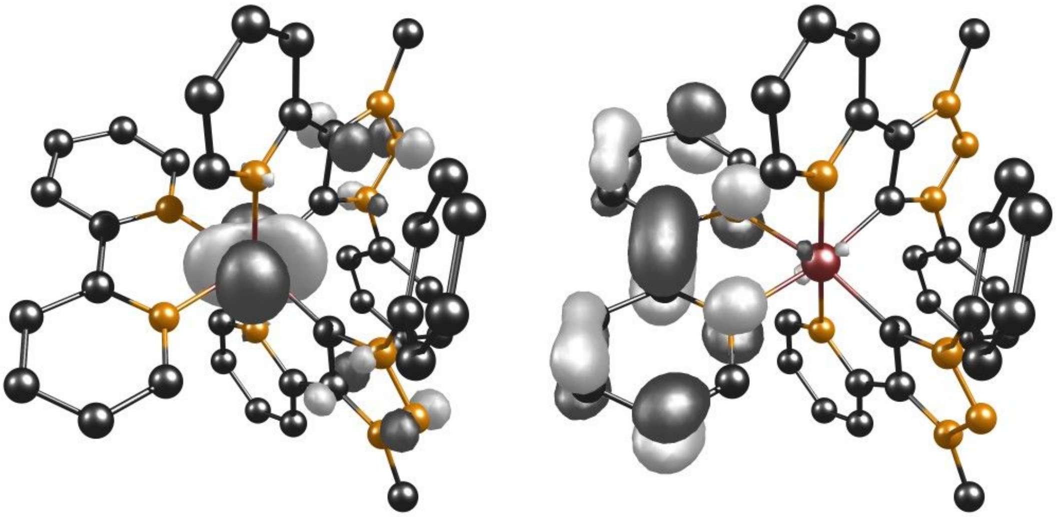

Figure S37. HOMO (left) and LUMO (right) of complex [RuL$_2$]$^{2+}$.

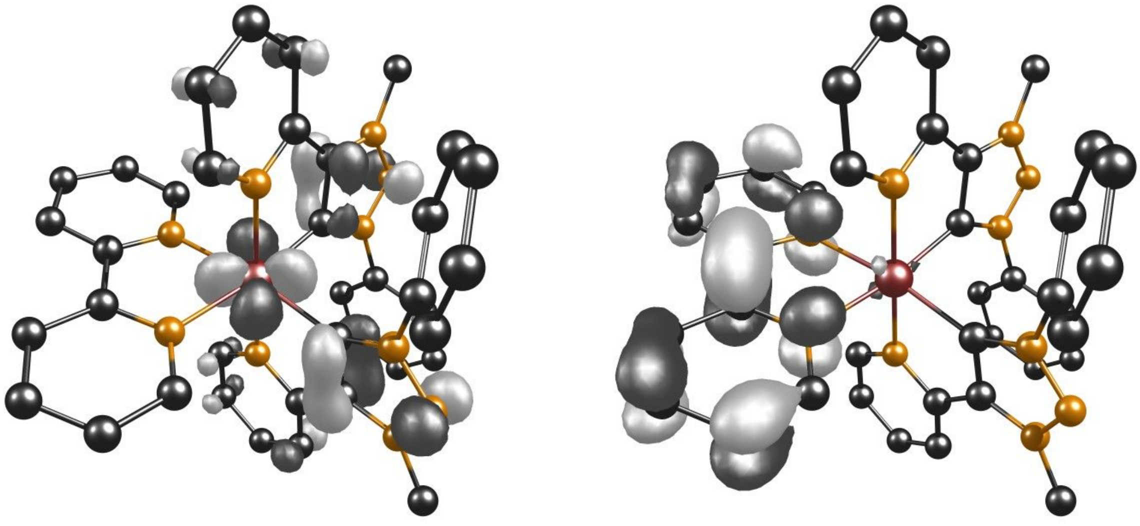

Figure S38. α-HOMO (left) and α-LUMO (right) of complex **[RuL$_2$]**$^{3+}$.

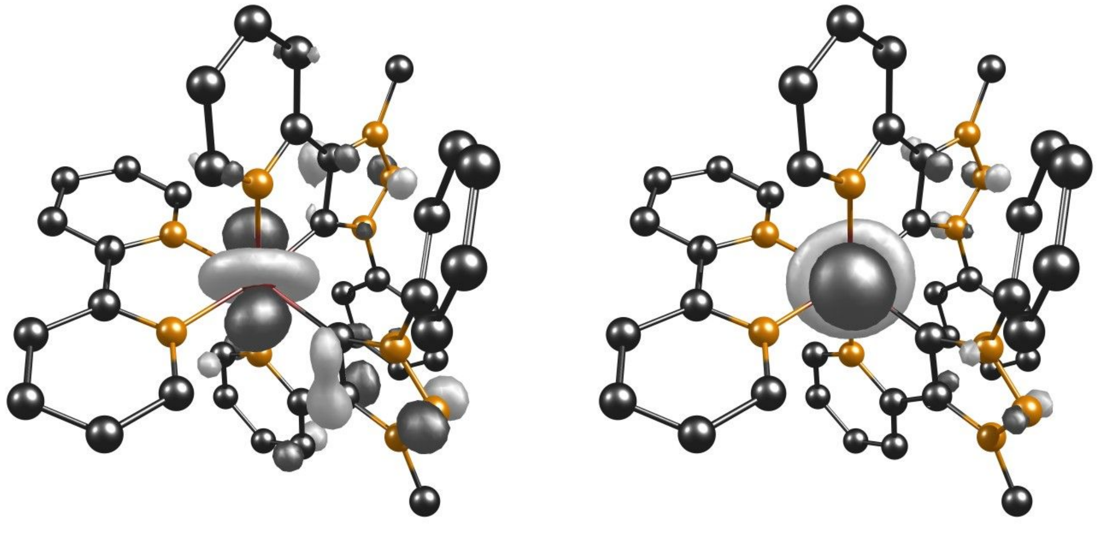

Figure S39. β-HOMO (left) and β-LUMO (right) of complex **[RuL$_2$]$^{3+}$**.

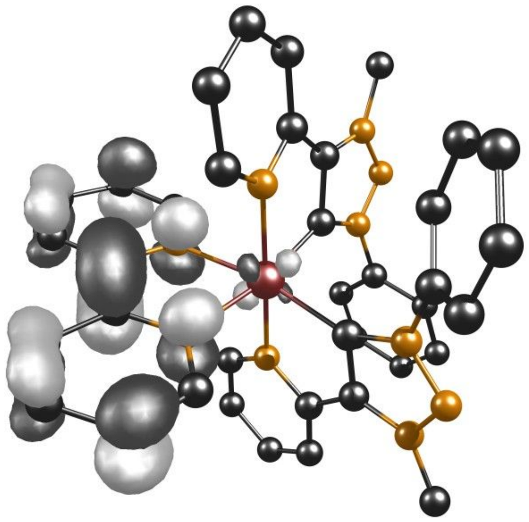
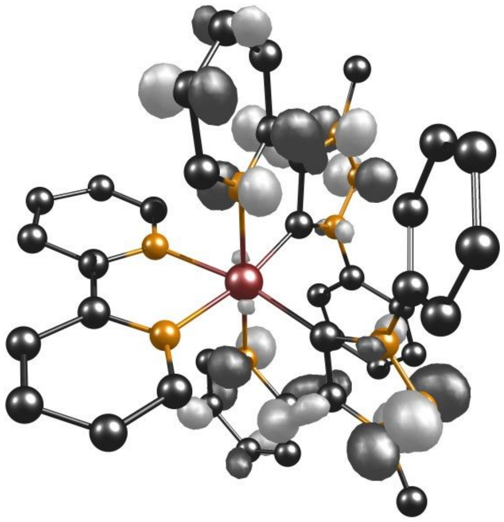

Figure S40 α-HOMO (left) and α-LUMO (right) of complex **[RuL$_2$]$^{1+}$.**

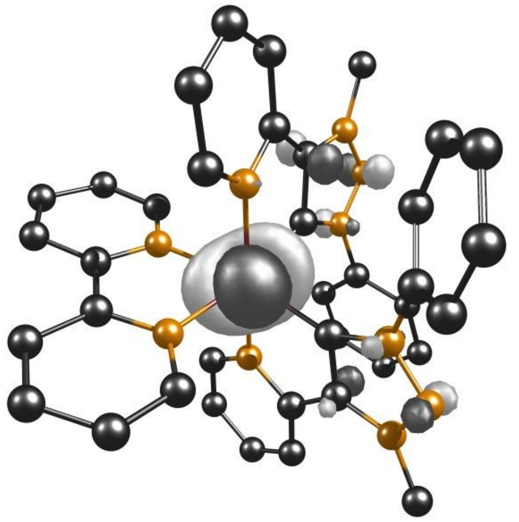
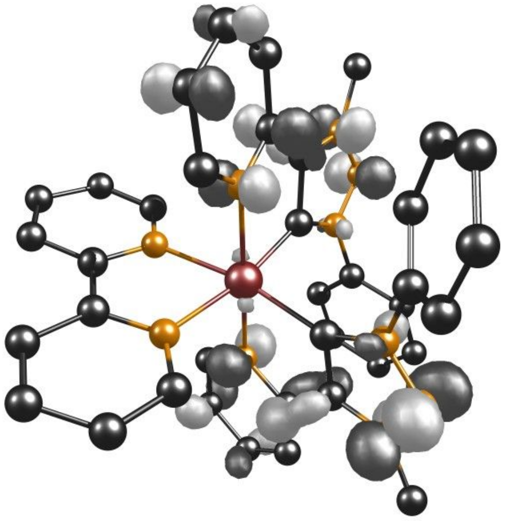

Figure S41. β-HOMO (left) and β-LUMO (right) of complex **[RuL$_2$]$^{1+}$**.

Table S13 Energies of selected orbitals.

MO	Energy / eV
$[\mathbf{RuL_2}]^{2+}_{\mathbf{HOMO}}$	− 6.0405
$[\mathbf{RuL_2}]^{2+}_{\mathbf{LUMO}}$	− 2.4643
$[\mathbf{RuL_2}]^{3+}_{\alpha-\mathbf{HOMO}}$	− 7.6380
$[\mathbf{RuL_2}]^{3+}_{\alpha-\mathbf{LUMO}}$	− 2.9195
$[\mathbf{RuL_2}]^{3+}_{\beta-\mathbf{HOMO}}$	− 7.5410
$[\mathbf{RuL_2}]^{3+}_{\beta-\mathbf{LUMO}}$	− 4.7982
$[\mathbf{RuL_2}]^{1+}_{\alpha-\mathbf{HOMO}}$	− 3.0205
$[\mathbf{RuL_2}]^{1+}_{\alpha-\mathbf{LUMO}}$	− 1.9293
$[\mathbf{RuL_2}]^{1+}_{\beta-\mathbf{HOMO}}$	− 5.4396
$[\mathbf{RuL_2}]^{1+}_{\beta-\mathbf{LUMO}}$	− 1.9090

Table S14. Selected experimental UV-Vis data of **$[RuL_2]^{2+}$** together with selected TD-DFT calculations.

State	$\lambda_{exp}(\lambda_{DFT})$ / nm	ε / 10^4 $M^{-1}cm^{-1}$(f)	Main contributing excitation (%)
**3**	424 (465)	1.99 (0.009)	HOMO → LUMO+1 (77)
**4**	424 (459)	1.99 (0.012)	HOMO → LUMO+2 (72)
**5**	424 (437)	1.99 (0.073)	HOMO-2 → LUMO+2 (90)
**8**	424 (392)	1.99 (0.110)	HOMO-1 → LUMO+2 (36)
			HOMO-2 → LUMO+1 (48)
**10**	424 (376)	1.99 (0.038)	HOMO → LUMO+3 (81)
**11**	368 (356)	1.24 (0.014)	HOMO → LUMO+4 (82)
**12**	368 (348)	1.24 (0.023)	HOMO-1 → LUMO+3 (71)
**13**	368 (330)	1.24 (0.019)	HOMO → LUMO+5 (62)
			HOMO-2 → LUMO+3 (22)
**16**	340 (331)	1.49 (0.020)	HOMO-2 → LUMO+4 (49)
			HOMO-1 → LUMO+4 (23)
**19**	340 (317)	1.49 (0.086)	HOMO-2 → LUMO+5 (38)
			HOMO-1 → LUMO+5 (13)
			HOMO → LUMO+7 (22)
**20**	340 (313)	1.49 (0.020)	HOMO-1 → LUMO+4 (12)
			HOMO-1 → LUMO+6 (26)
			HOMO → LUMO+7 (17)
**22**	340 (309)	1.49 (0.065)	HOMO-1 → LUMO+5 (52)

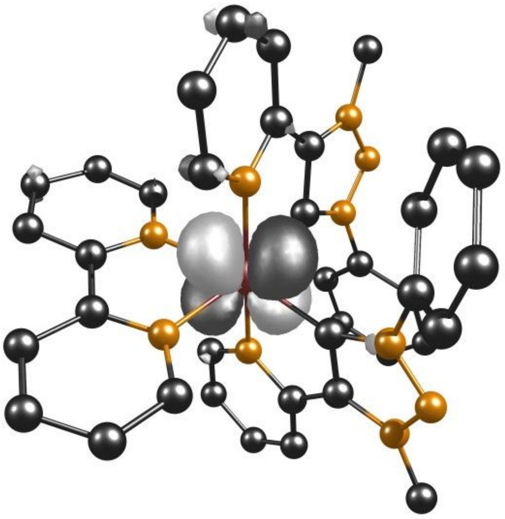

HOMO-2

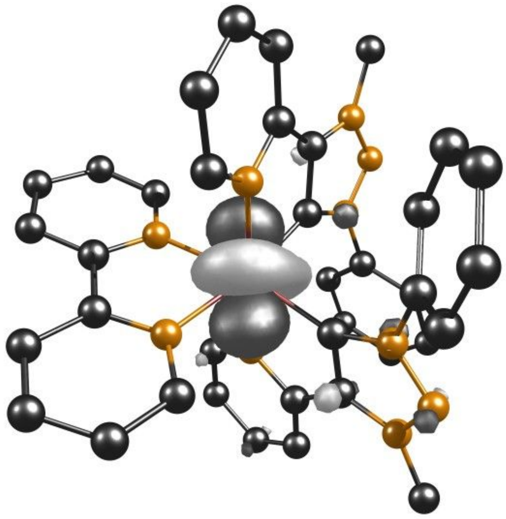

HOMO-1

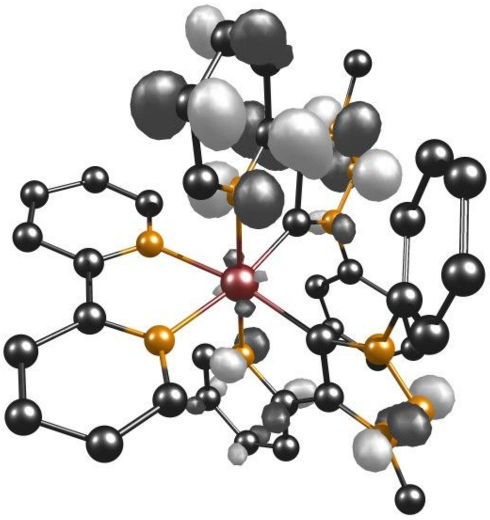

LUMO+1

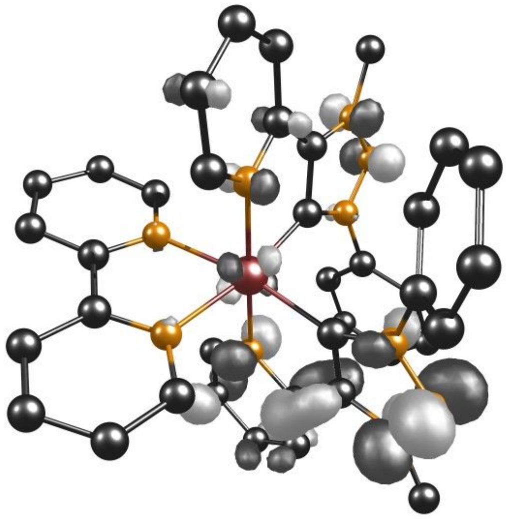

LUMO+2

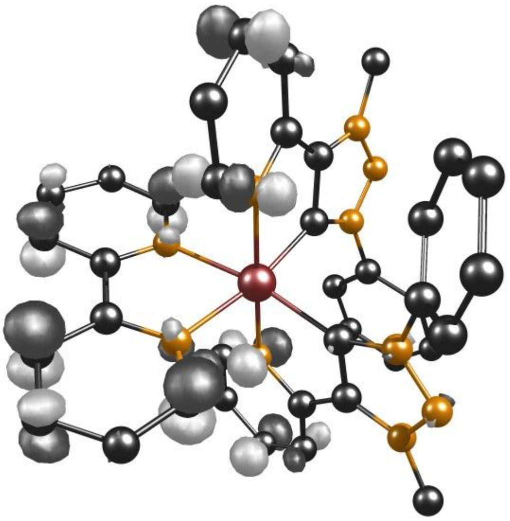

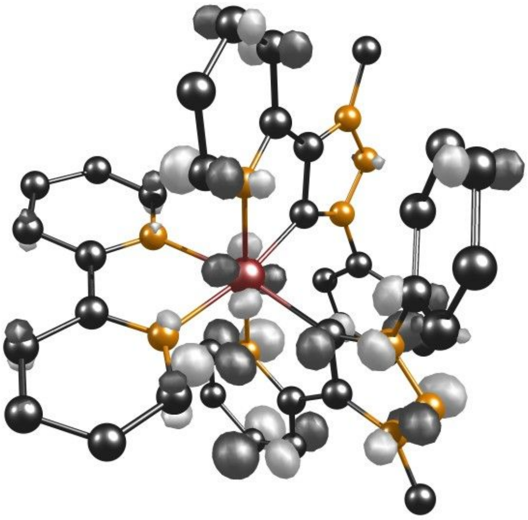

LUMO+3 **LUMO+4**

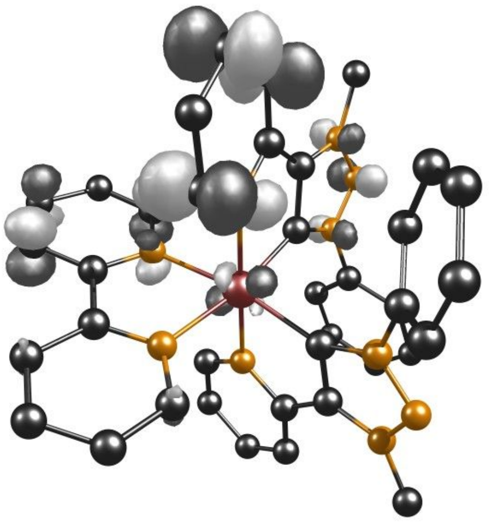

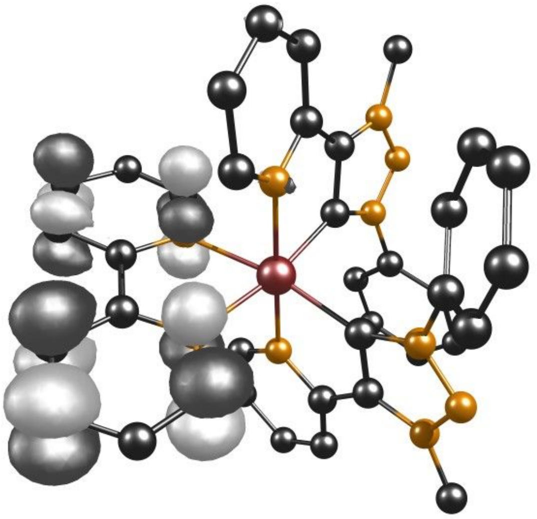

LUMO+5

LUMO+6

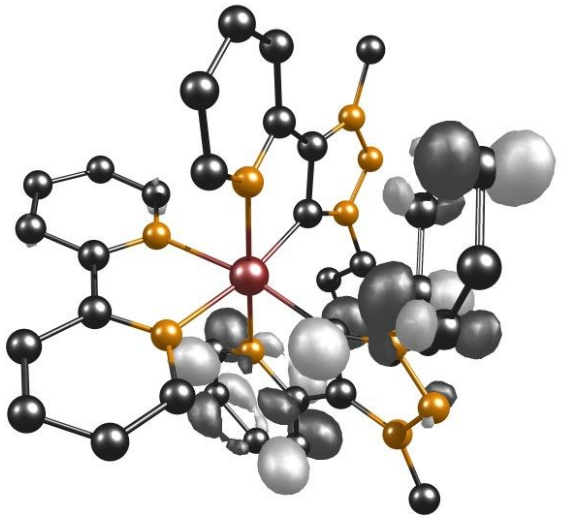

LUMO+7

Figure S42. Involved TD-DFT orbitals of complex **$[RuL_2]^{2+}$**.

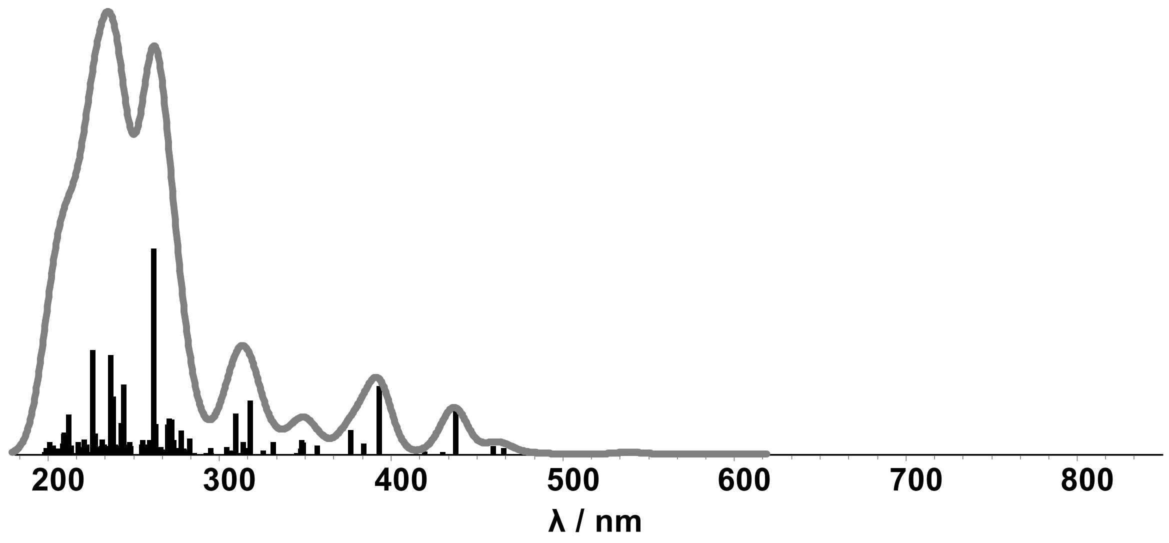

Figure S43. Calculated TD-DFT spectrum with discrete transitions of **[RuL$_2$]**$^{2+}$.

Table S15. Selected experimental UV-Vis data of **[RuL$_2$]**$^{3+}$ together with selected TD-DFT calculations.

	$\lambda_{exp}(\lambda_{DFT})$ / nm	ε / 10^4 $M^{-1}cm^{-1}$(f)	Main contributing excitation (%)
**3**	722 (556)	0.25 (0.024)	β-HOMO-2 → β-LUMO (84)
**5**	670 (511)	0.16 (0.002)	β-HOMO-8 → β-LUMO (19)
			β-HOMO-4 → β-LUMO (54)
**6**	670 (501)	0.16 (0.005)	β-HOMO-8 → β-LUMO (36)
			β-HOMO-6 → β-LUMO (30)
**8**	572 (497)	0.05 (0.002)	β-HOMO-8 → β-LUMO (10)
			β-HOMO-7 → β-LUMO (16)
			β-HOMO-6 → β-LUMO (43)
			β-HOMO-4 → β-LUMO (23)
**9**	572 (497)	0.05 (0.001)	β-HOMO-8 → β-LUMO (14)
			β-HOMO-7 → β-LUMO (54)
**10**	424 (390)	0.05 (0.005)	β-HOMO-9 → β-LUMO (76)

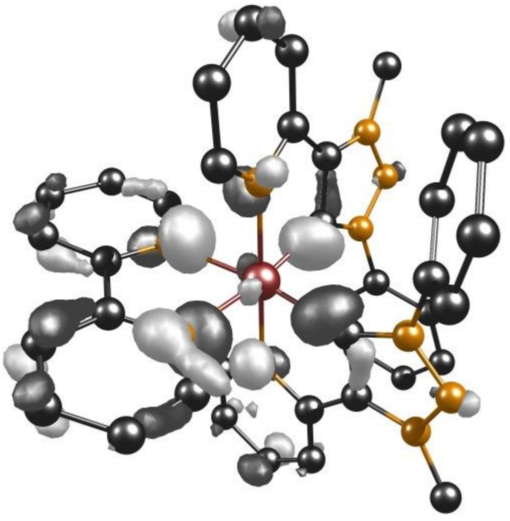

***β*-HOMO-9**

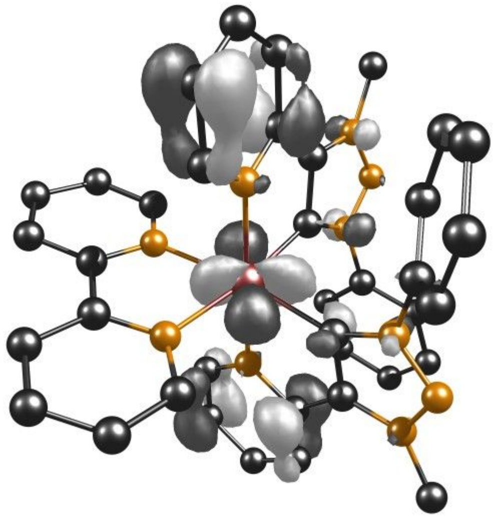

***β*-HOMO-8**

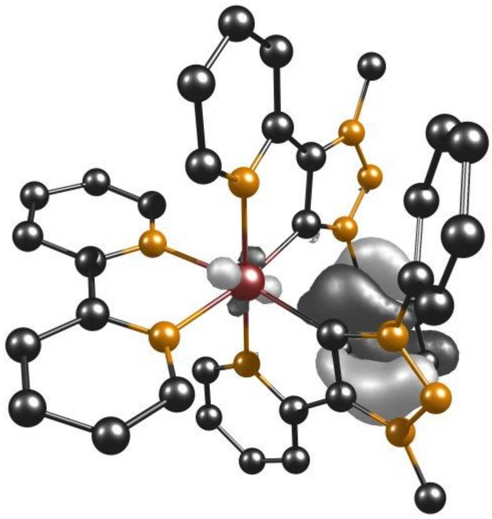

***β*-HOMO-7**

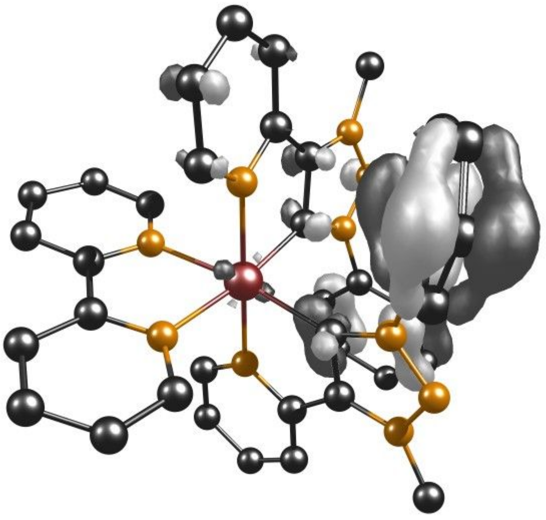

***β*-HOMO-6**

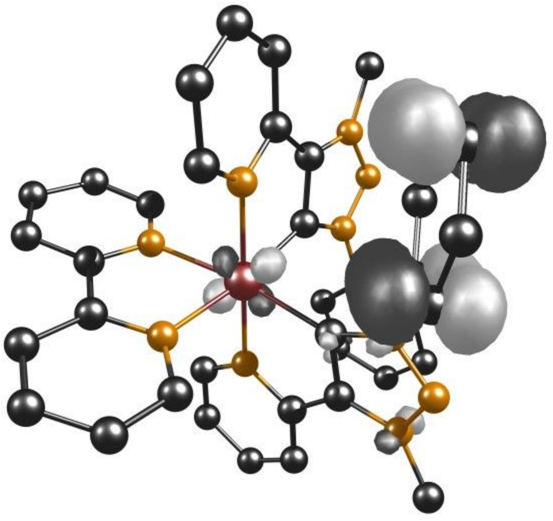

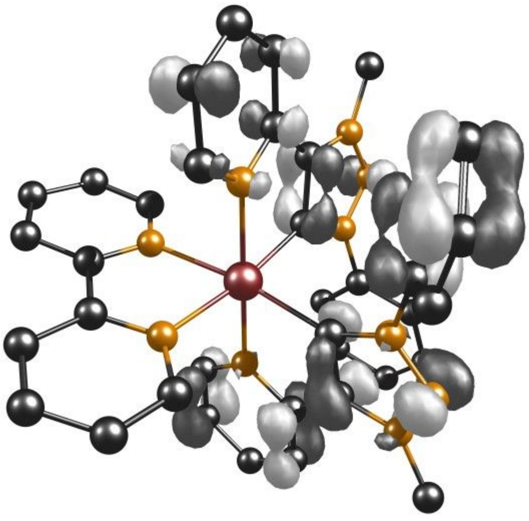

***β*-HOMO-4** **β-HOMO-2**

Figure S44. Involved TD-DFT orbitals of complex **[RuL$_2$]**$^{3+}$.

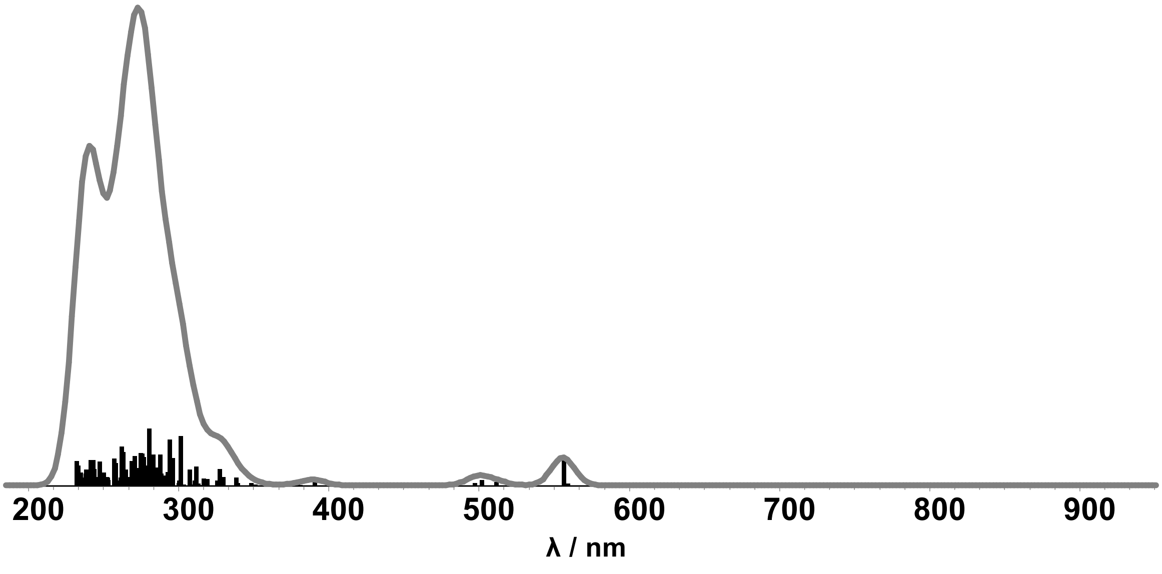

Figure S45. Calculated TD-DFT spectrum with discrete transitions of **[RuL$_2$]**$^{3+}$.

Table S16. Selected experimental UV-Vis data of **$[RuL_2]^{1+}$** together with selected TD-DFT calculations.

State	$\lambda_{exp}(\lambda_{DFT})$ / nm	ε / 10^4 $M^{-1}cm^{-1}(f)$	Main contributing excitation (%)
**3**	1500-1080 (1119)	0.01 (0.002)	α-HOMO → α-LUMO+1 (71)
			α-HOMO → α-LUMO+6 (15)
**5**	982 (887)	0.13 (0.004)	α-HOMO → α-LUMO+5 (10)
			α-HOMO → α-LUMO+6 (19)
			α-HOMO → α-LUMO+8 (15)
**10**	982 (883)	0.13 (0.001)	α-HOMO → α-LUMO+7 (46)
**7**	982 (845)	0.13 (0.006)	α-HOMO → α-LUMO+5 (88)
			α-HOMO → α-LUMO+6 (70)
			α-HOMO → α-LUMO+8 (11)
**8**	982 (825)	0.13 (0.001)	α-HOMO → α-LUMO+5 (10)
			α-HOMO → α-LUMO+10 (80)
**6**	866 (741)	0.13 (0.015)	α-HOMO → α-LUMO+8 (10)
			α-HOMO → α-LUMO+9 (16)
**9**	866 (726)	0.13 (0.002)	α-HOMO → α-LUMO+5 (20)
			α-HOMO → α-LUMO+8 (12)
			α-HOMO → α-LUMO+9 (15)
			α-HOMO → α-LUMO+10 (45)

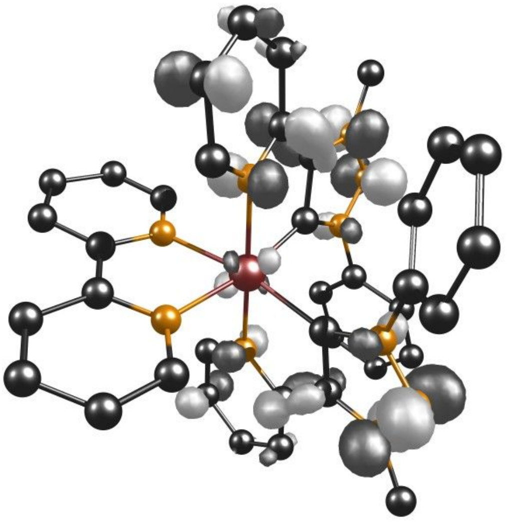

α-LUMO+1

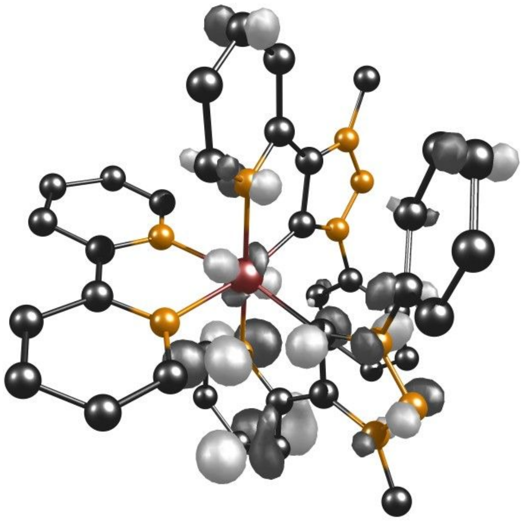

α-LUMO+2

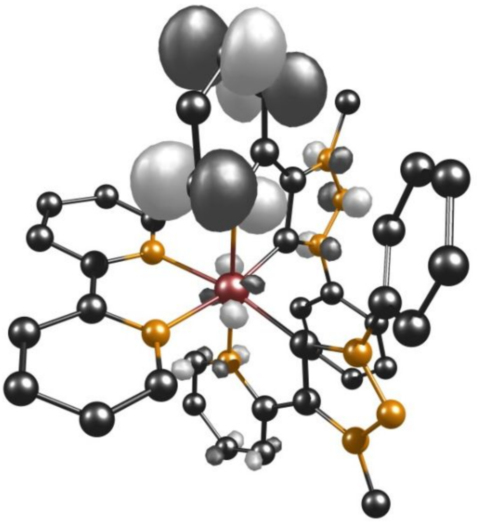

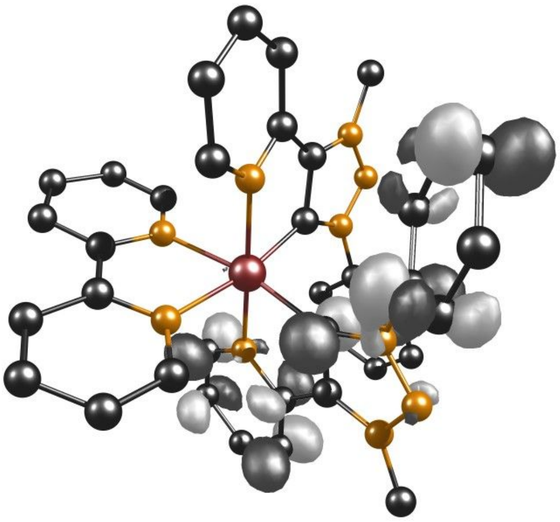

α-LUMO+3

α-LUMO+4

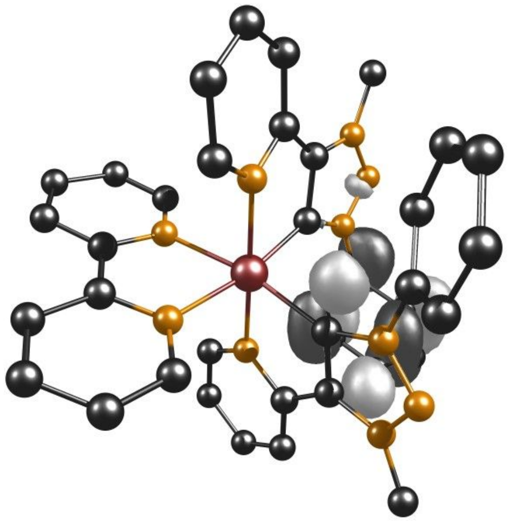

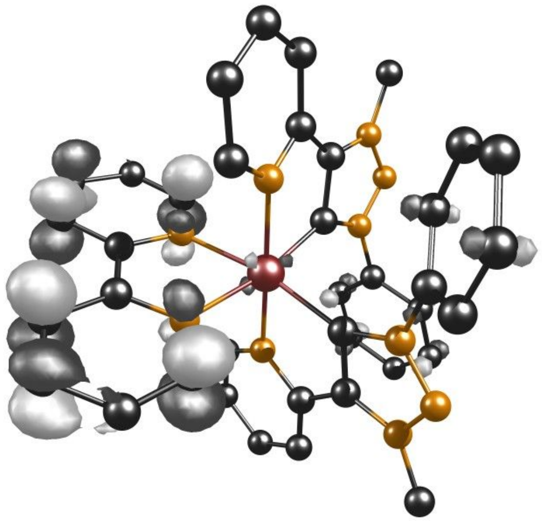

α-LUMO+5

α-LUMO+6

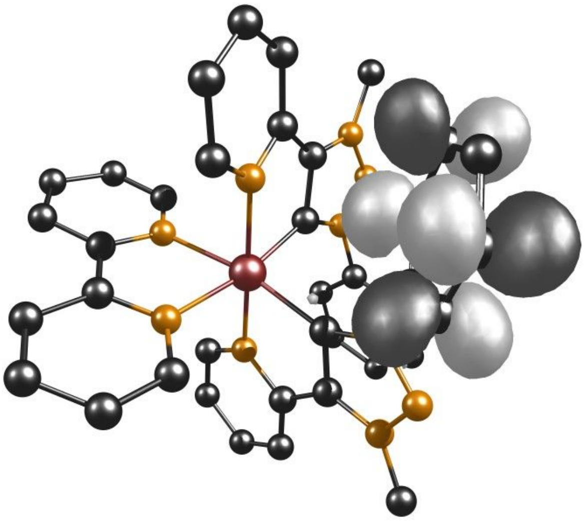

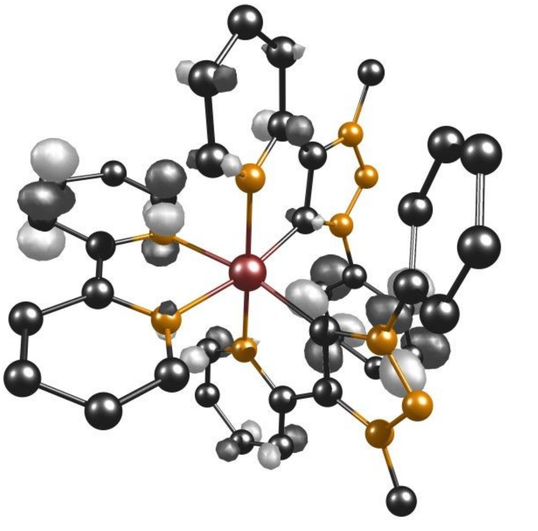

α-LUMO+7 α-LUMO+8

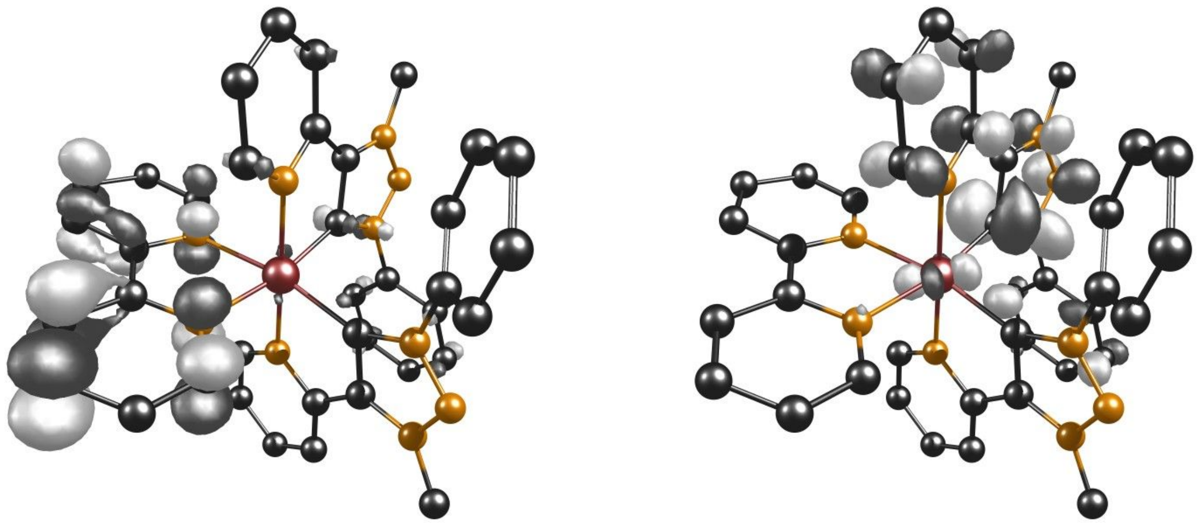

α-LUMO+9 α-LUMO+10

Figure 46. Involved TD-DFT orbitals of complex **[RuL$_2$]$^{1+}$**.

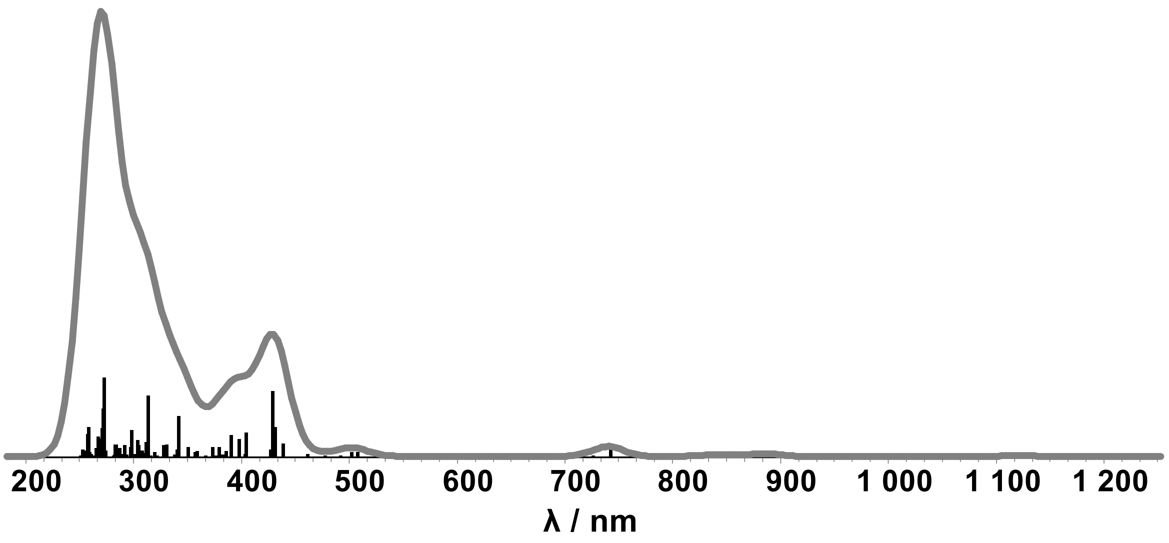

Figure S47. Calculated TD-DFT spectrum with discrete transitions of **[RuL$_2$]$^{1+}$**.

Table S17. Selected MO energies of **[RuL$_2$]$^{2+}$**, **[RuL$_2$]$^{3+}$** and **[RuL$_2$]$^{1+}$**.

**[RuL$_1$]$^{2+}$**		**[RuL$_1$]$^{3+}$**		**[RuL$_1$]$^{1+}$**	
**MO**	**Energy / eV**	**MO**	**Energy / eV**	**MO**	**Energy / eV**
**HOMO-2**	– 6.3020	***β*-HOMO-9**	– 9.3907	***α*-LUMO+1**	– 1.8892
**HOMO-1**	– 6.2797	***β*-HOMO-8**	– 8.5852	***α*-LUMO+2**	– 1.3219
**LUMO+1**	– 2.3203	***β*-HOMO-7**	– 8.2339	***α*-LUMO+3**	– 1.1467
**LUMO+2**	– 2.2247	***β*-HOMO-6**	– 8.1532	***α*-LUMO+4**	– 0.9503
**LUMO+3**	– 1.7772	***β*-HOMO-4**	– 8.0412	***α*-LUMO+5**	– 0.7124
**LUMO+4**	– 1.5824	***β*-HOMO-2**	– 7.8986	***α*-LUMO+6**	– 0.6989
**LUMO+5**	– 1.5164			***α*-LUMO+7**	– 0.5901
**LUMO+6**	– 1.3897			***α*-LUMO+8**	– 0.5374
**LUMO+7**	– 1.2295			***α*-LUMO+9**	– 0.4770
				***α*-LUMO+10**	– 0.4213

Table S18. XYZ coordinates of optimized **$[RuL_2]^{2+}$**.

	x	y	z
Ru	3.38342	10.4003	5.83929
N	4.48826	13.14392	4.72407
N	2.83431	8.55939	6.71893
N	4.71015	10.75104	7.41081
N	4.64758	9.00312	4.86466
N	1.98348	12.48964	9.04273
N	1.01962	12.05068	7.25072
N	2.13019	10.10889	4.1828
N	3.66975	13.09224	2.81111
N	4.40575	13.83211	3.59029
N	0.8428	12.63141	8.434
C	3.8298	11.9553	4.70785
C	3.27835	11.95217	3.4277
C	2.26646	11.52603	7.05969
C	2.88139	11.83876	8.27231
C	3.40521	7.45794	6.19058
C	2.33976	10.90941	3.10025
C	4.24196	11.43038	8.49109
C	2.12843	13.00469	10.38901
H	1.18587	13.4658	10.66974
H	2.92447	13.74762	10.4116
H	2.35963	12.18148	11.06369
C	4.87689	15.09487	7.82431
H	4.2712	15.71305	8.47646
C	5.03111	11.67541	9.60408
H	4.63215	12.2199	10.44754
C	5.09995	13.74612	5.86703
C	4.41689	7.70616	5.15516
C	5.57114	9.30416	3.9471
H	5.72486	10.35659	3.74623
C	5.97356	10.32415	7.43851
H	6.3197	9.79214	6.56333
C	4.31318	14.54233	6.68494
H	3.27484	14.71945	6.43039
C	6.42443	13.48159	6.16318
H	7.00788	12.84166	5.51176
C	-0.99278	12.26046	4.17362
H	-0.8596	12.51376	3.12854
C	6.97992	14.0424	7.30461
H	8.01282	13.83667	7.55819
C	-0.09362	11.99878	6.36892
C	1.92336	8.41685	7.68448
H	1.50266	9.3294	8.08401
C	6.33738	11.22275	9.61627
H	6.97147	11.41212	10.47333
C	1.22667	9.13358	4.08202
H	1.06575	8.53339	4.96656
C	1.66126	10.71495	1.90644
H	1.8582	11.35296	1.05636
C	6.20678	14.84505	8.13283
H	6.64272	15.27366	9.02759
C	6.30249	8.34251	3.27684
H	7.04095	8.64422	2.54595
C	0.08768	12.33056	5.03771
H	1.06169	12.65204	4.69565
C	3.04319	6.18613	6.61582
H	3.504	5.31172	6.17799
C	0.73906	9.68598	1.81656
H	0.20602	9.50943	0.8906
C	1.51858	7.18445	8.15636
H	0.77172	7.12856	8.93703
C	6.81749	10.54094	8.51056
H	7.83395	10.17251	8.46952
C	2.08551	6.04605	7.60401
H	1.7866	5.06119	7.94192
C	3.39524	13.54788	1.4624
H	2.32113	13.68148	1.33617
H	3.76966	12.81203	0.75259
H	3.90614	14.49603	1.32355
C	0.51217	8.8877	2.92476
H	-0.20833	8.08112	2.90486
C	-2.41188	11.57148	5.9902
H	-3.38433	11.27493	6.36487
C	-1.33299	11.61921	6.85955
H	-1.44605	11.35677	7.90423
C	-2.24257	11.88551	4.64858
H	-3.08731	11.83937	3.97148
C	6.06171	7.00815	3.56125

H	6.60882	6.22406	3.05309
C	5.10839	6.68824	4.51065
H	4.90393	5.6537	4.74846

Table S19. XYZ coordinates of optimized **$[RuL_2]^{3+}$**.

	x	y	z
Ru	3.40136	10.3728	5.83502
N	4.43373	13.18182	4.77366
N	2.87043	8.54557	6.75009
N	4.70641	10.70203	7.42455
N	4.63842	8.96725	4.83647
N	2.02132	12.59841	8.96288
N	1.0549	12.11743	7.18074
N	2.1642	10.08104	4.16584
N	3.59234	13.15574	2.86842
N	4.32287	13.8847	3.6638
N	0.89076	12.73836	8.33486
C	3.80744	11.98391	4.71859
C	3.23938	11.98802	3.45719
C	2.28528	11.56011	7.03334
C	2.90721	11.89082	8.2264
C	3.41887	7.43925	6.20608
C	2.32449	10.92863	3.11341
C	4.25382	11.44747	8.46704
C	2.16714	13.15707	10.29371
H	1.2351	13.65485	10.5442
H	2.98535	13.87564	10.29647
H	2.36357	12.3483	10.99598
C	4.88245	15.10457	7.88003
H	4.30023	15.74708	8.52965
C	5.03977	11.69894	9.5767
H	4.65998	12.2952	10.39335
C	5.06533	13.75693	5.92365
C	4.40989	7.67496	5.14976
C	5.53706	9.26971	3.89611
H	5.68446	10.31986	3.67932
C	5.94103	10.20031	7.48154
H	6.26905	9.61004	6.63748
C	4.30145	14.57886	6.73661
H	3.27392	14.80214	6.47424
C	6.3777	13.43672	6.21841
H	6.94181	12.7846	5.56202
C	-0.98651	12.29402	4.11752
H	-0.88221	12.59445	3.08197
C	6.94797	13.97405	7.36374
H	7.97295	13.73359	7.61787
C	-0.0615	12.02659	6.29767
C	1.9879	8.42888	7.74415
H	1.59196	9.34832	8.1525
C	6.32056	11.1779	9.62151
H	6.95405	11.37041	10.47797
C	1.30214	9.0703	4.0534
H	1.18478	8.42664	4.91355
C	1.6282	10.75606	1.93016
H	1.78127	11.43254	1.10135
C	6.20071	14.80136	8.19172
H	6.64948	15.21111	9.08868
C	6.25621	8.30073	3.22454
H	6.9796	8.59475	2.47631
C	0.09147	12.41343	4.9787
H	1.03316	12.82656	4.64388
C	3.05418	6.17762	6.64842
H	3.49182	5.29477	6.2046
C	0.7396	9.69846	1.82556
H	0.18814	9.54017	0.90755
C	1.58375	7.20297	8.23154
H	0.86021	7.15788	9.03389
C	6.77935	10.42309	8.55586
H	7.77426	9.99933	8.54801
C	2.1215	6.05879	7.66464
H	1.81999	5.07908	8.01437
C	3.28915	13.64377	1.53497
H	2.21196	13.77415	1.43578
H	3.65602	12.92569	0.80385
H	3.79345	14.5975	1.41317
C	0.56835	8.84846	2.90393
H	-0.12398	8.01868	2.86849
C	-2.33796	11.44046	5.9186
H	-3.2834	11.06032	6.28607
C	-1.26198	11.54032	6.78613
H	-1.34769	11.23749	7.82246
C	-2.19934	11.8094	4.58745
H	-3.04271	11.72278	3.91289
C	6.02122	6.97053	3.52965

H	6.55938	6.18246	3.01853
C	5.08638	6.6541	4.49944
H	4.88772	5.62157	4.74937

Table S20. XYZ coordinates of optimized **[RuL$_2$]$^{1+}$**.

	x	y	z
Ru	3.38159	10.42637	5.83845
N	4.47231	13.161	4.69553
N	2.81296	8.59741	6.72493
N	4.70615	10.76227	7.40382
N	4.66365	9.04712	4.8747
N	1.97885	12.45103	9.08096
N	1.01538	12.05383	7.2793
N	2.14558	10.10437	4.18575
N	3.6526	13.08775	2.78406
N	4.38324	13.84262	3.55644
N	0.83541	12.60505	8.47818
C	3.82389	11.96503	4.69233
C	3.27329	11.9483	3.40847
C	2.26443	11.53439	7.07228
C	2.87838	11.82354	8.29449
C	3.4162	7.48448	6.17117
C	2.34954	10.89139	3.09121
C	4.24004	11.4164	8.50151
C	2.12275	12.93146	10.43929
H	1.17941	13.38302	10.73294
H	2.91712	13.67528	10.48235
H	2.35722	12.09261	11.0935
C	4.8672	15.09622	7.80559
H	4.26178	15.7062	8.46577
C	5.03389	11.64078	9.61697
H	4.63613	12.16598	10.47335
C	5.08614	13.76491	5.83488
C	4.40429	7.72545	5.18413
C	5.59647	9.32855	3.96014
H	5.75508	10.38256	3.7585
C	5.97177	10.33926	7.41837
H	6.30755	9.82654	6.52759
C	4.30117	14.55231	6.66313
H	3.26013	14.72454	6.41662
C	6.41189	13.50073	6.1269
H	6.9932	12.86398	5.4705
C	-0.97357	12.27834	4.19015
H	-0.82525	12.50958	3.14186
C	6.97024	14.0522	7.27136
H	8.00379	13.84451	7.52101
C	-0.09472	12.02285	6.39572
C	1.89369	8.43363	7.67929
H	1.46627	9.34528	8.08009
C	6.34104	11.19178	9.61535
H	6.97792	11.36312	10.47429
C	1.2662	9.10633	4.09066
H	1.12256	8.51676	4.98532
C	1.68343	10.66663	1.89403
H	1.87599	11.29631	1.03639
C	6.19829	14.84644	8.10868
H	6.63566	15.26727	9.00653
C	6.33483	8.37734	3.29164
H	7.07804	8.67699	2.56511
C	0.10314	12.32911	5.06032
H	1.08973	12.61125	4.72005
C	3.01667	6.19662	6.60918
H	3.47254	5.31898	6.16856
C	0.78447	9.61732	1.81179
H	0.26233	9.41558	0.88451
C	1.47641	7.20903	8.14831
H	0.72459	7.1499	8.92352
C	6.81803	10.53384	8.49259
H	7.83506	10.16756	8.44108
C	2.06158	6.05721	7.57751
H	1.75599	5.06958	7.90571
C	3.36951	13.53216	1.43425
H	2.29322	13.64835	1.30795
H	3.75275	12.79926	0.72597
H	3.8655	14.48736	1.28874
C	0.56848	8.82814	2.93029
H	-0.13052	8.00248	2.91387
C	-2.42438	11.66101	6.00654
H	-3.40763	11.39956	6.37944
C	-1.3497	11.68919	6.88226
H	-1.47746	11.44463	7.92954
C	-2.23783	11.94875	4.66136
H	-3.07919	11.91674	3.9792
C	6.07812	7.02122	3.58657

H	6.62766	6.23439	3.08245
C	5.12801	6.70515	4.5169
H	4.92196	5.66818	4.74997

7.30 (TD)-DFT of $[RuL_3](PF_6)_2$

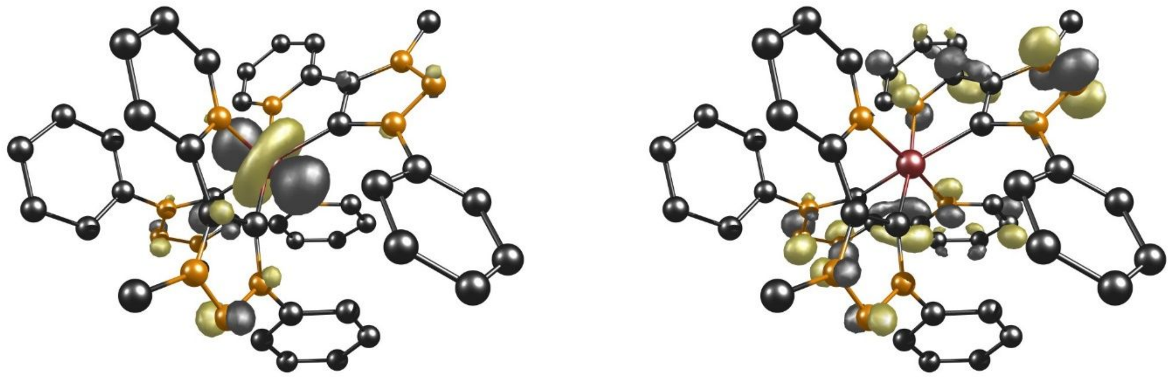

Figure S48.HOMO (left) and LUMO (right) of complex $\mathbf{[RuL_3]^{2+}}$.

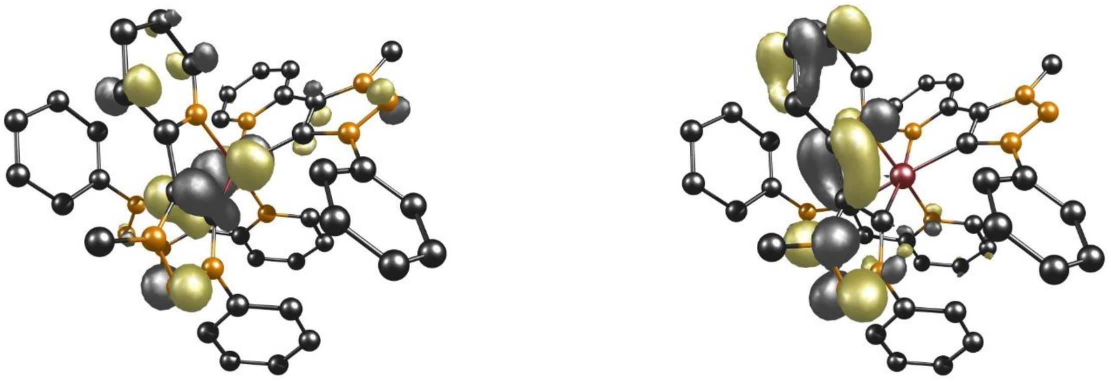

Figure S49. α-HOMO (left) and α-LUMO (right) of complex $\mathbf{[RuL_3]^{3+}}$.

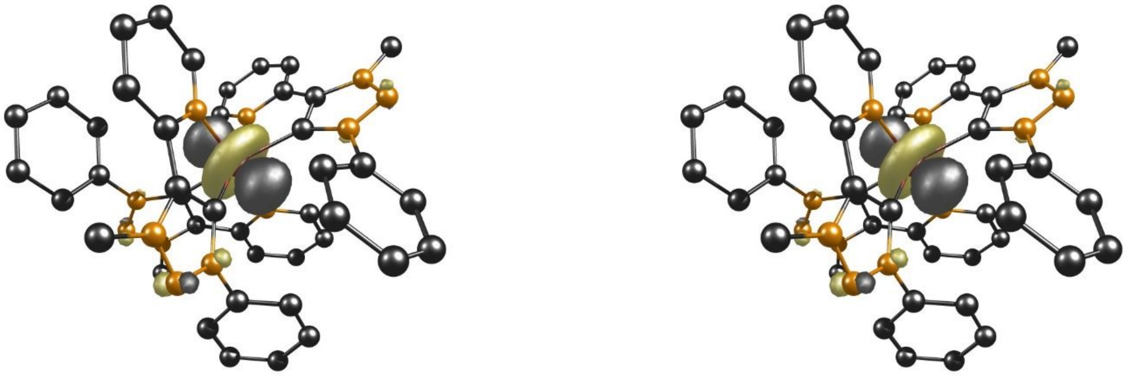

Figure S50. β-HOMO (left) and β-LUMO (right) of complex $\mathbf{[RuL_3]^{3+}}$.

Table S21. Energies of selected orbitals $\mathbf{[RuL_3](PF_6)_2}$.

MO	Energy / eV
$[\mathbf{RuL_3}]^{2+}_{\mathbf{HOMO}}$	− 5.7666
$[\mathbf{RuL_3}]^{2+}_{\mathbf{LUMO}}$	− 2.1618
$[\mathbf{RuL_3}]^{3+}_{\alpha-\mathbf{HOMO}}$	− 7.5251
$[\mathbf{RuL_3}]^{3+}_{\alpha-\mathbf{LUMO}}$	− 2.7580
$[\mathbf{RuL_3}]^{3+}_{\beta-\mathbf{HOMO}}$	− 7.3155
$[\mathbf{RuL_3}]^{3+}_{\beta-\mathbf{LUMO}}$	− 4.5919

Table S22. Selected experimental UV/vis data of $\mathbf{[RuL_3]^{2+}}$ together with selected TD-DFT calculations.

State	$\lambda_{exp}(\lambda_{DFT})$ / nm	ε / 10^4 $M^{-1}cm^{-1}$(*f*)	Main contributing excitation (%)
**2**	450 (493)	1.04 (0.005)	HOMO → LUMO (62)<br>HOMO → LUMO+1 (30)
**1**	450 (478)	1.04 (0.003)	HOMO → LUMO (31)<br>HOMO → LUMO+1 (53)
**3**	424 (467)	1.20 (0.010)	HOMO → LUMO+1 (10)<br>HOMO → LUMO+2 (82)
**4**	424 (454)	1.20 (0.011)	HOMO-1 → LUMO (42)<br>HOMO-1 → LUMO+1 (24)<br>HOMO-1 → LUMO+2 (24)
**5**	424 (453)	1.20 (0.006)	HOMO-1 → LUMO (46)<br>HOMO-1 → LUMO+1 (36)
**6**	372 (428)	0.96 (0.009)	HOMO-2 → LUMO (34)<br>HOMO-2 → LUMO+1 (33)<br>HOMO-2 → LUMO+2 (26)
**7**	372 (418)	0.96 (0.044)	HOMO-1 → LUMO+1 (23)<br>HOMO-1 → LUMO+2 (48)<br>HOMO → LUMO+3 (13)
**8**	372 (402)	0.96 (0.014)	HOMO-1 → LUMO (48)<br>HOMO-1 → LUMO+1 (12)<br>HOMO-1 → LUMO+2 (15)<br>HOMO → LUMO+3 (14)
**9**	349 (391)	1.12 (0.082)	HOMO-2 → LUMO (11)<br>HOMO-2 → LUMO+1 (11)<br>HOMO → LUMO+3 (61)
**10**	349 (389)	1.12 (0.048)	HOMO-2 → LUMO+1 (45)<br>HOMO-2 → LUMO+2 (30)

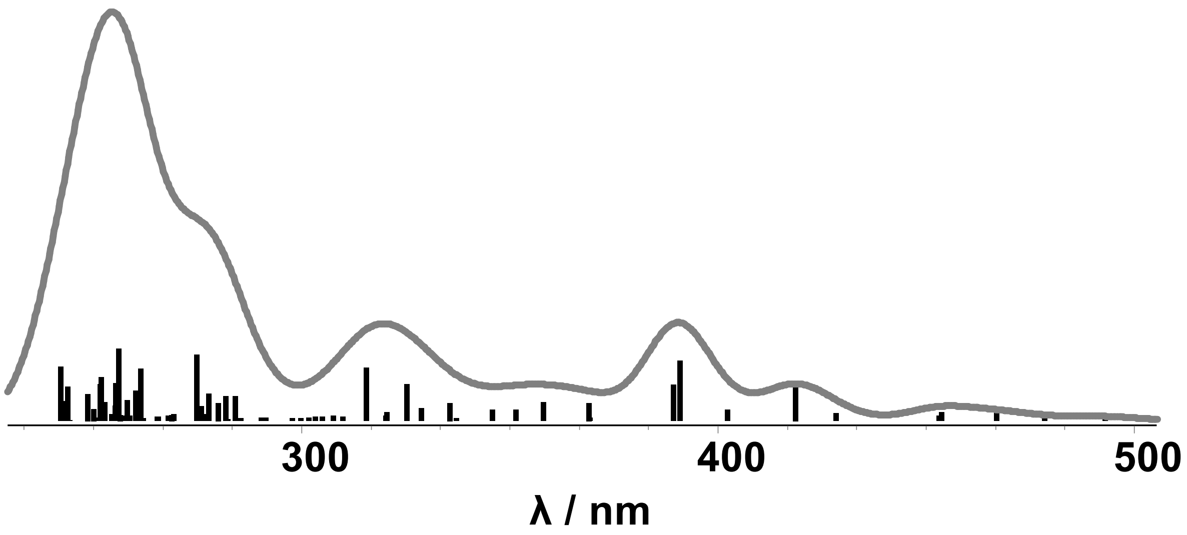

Figure S51. Calculated TD-DFT spectrum with discrete transitions of $\mathbf{[RuL_3]}^{2+}$.

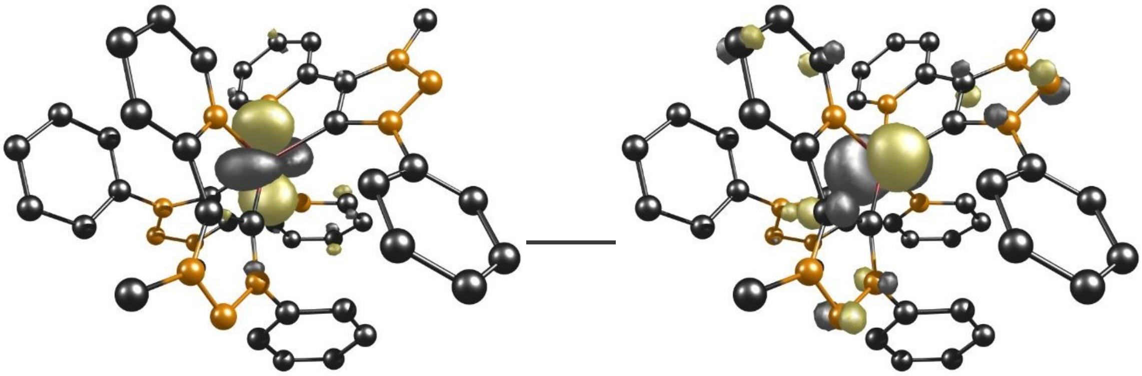

HOMO-2 **HOMO-1**

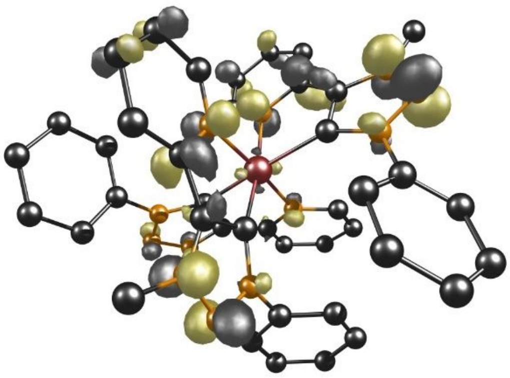

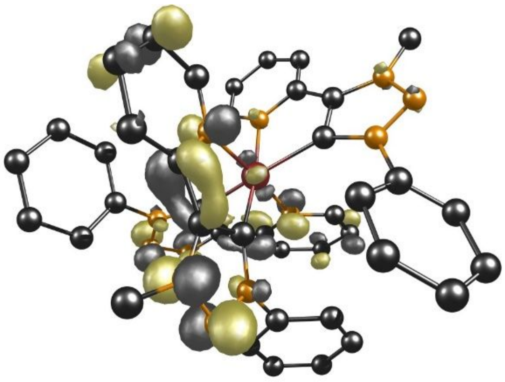

LUMO+1 **LUMO+2**

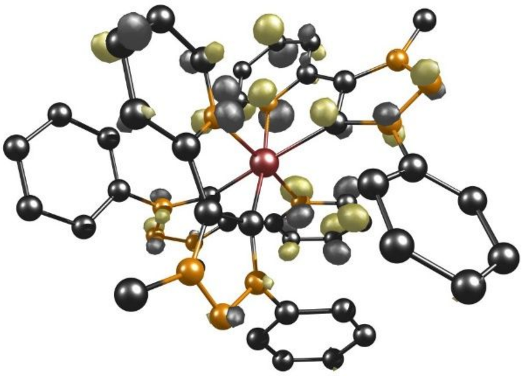

LUMO+3

Figure S52. Involved TD-DFT orbitals of complex **[RuL$_3$]**$^{2+}$.

Table S23. Selected experimental UV/vis data of **[RuL$_3$]**$^{3+}$ together with selected TD-DFT calculations.

State	$\lambda_{exp}(\lambda_{DFT})$ / nm	ε / 10^4 M^{-1}cm^{-1}(*f*)	Main contributing excitation (%)
**3**	689-579 (545)	0.15 (0.011)	β-HOMO-2→ β-LUMO (86)
**4**	689-579 (520)	0.15 (0.011)	β-HOMO-9 → β-LUMO (16)
			β-HOMO-3 → β-LUMO (60)
**7**	440-506 (477)	0.09 (0.002)	β-HOMO-9 → β-LUMO (12)
			β-HOMO-8 → β-LUMO (11)
			β-HOMO-7 → β-LUMO (32)
			β-HOMO-3 → β-LUMO (24)
**5**	440-506 (467)	0.09 (0.003)	β-HOMO-10 → β-LUMO (16)
			β-HOMO-5 → β-LUMO (31)

			β-HOMO-4 → β-LUMO (17)
**6**	440-506 (462)	0.09 (0.002)	β-HOMO-11 → β-LUMO (22)<br>β-HOMO-9 → β-LUMO (15)<br>β-HOMO-5 → β-LUMO (15)<br>β-HOMO-4 → β-LUMO (22)
**11**	440-506 (436)	0.09 (0.003)	β-HOMO-11 → β-LUMO (15)<br>β-HOMO-10 → β-LUMO (21)<br>β-HOMO-8 → β-LUMO (22)<br>β-HOMO-5 → β-LUMO (18)

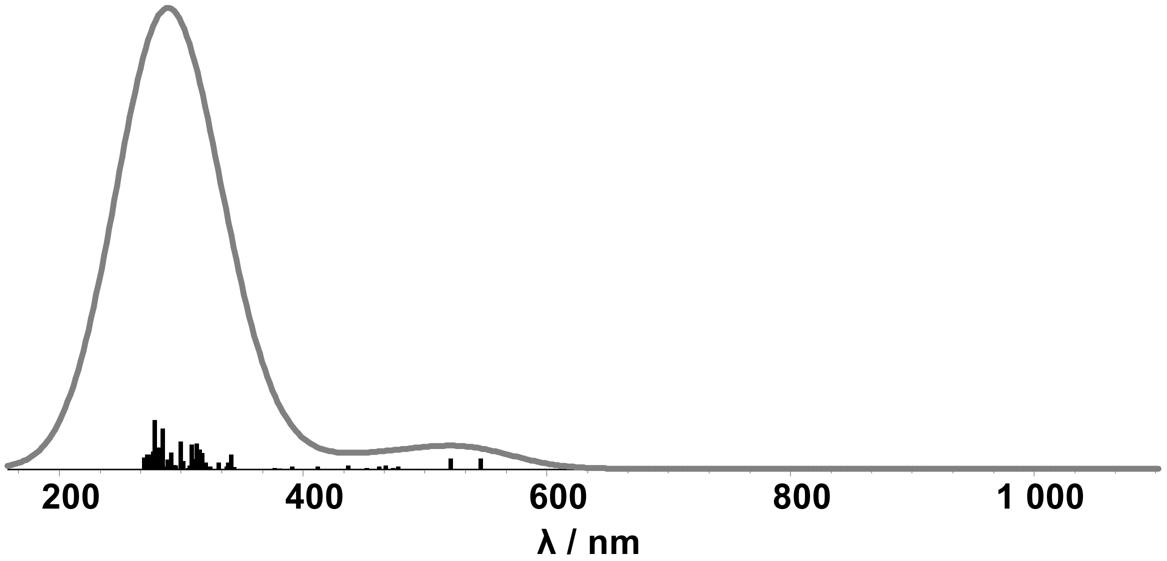

Figure S35. Calculated TD-DFT spectrum with discrete transitions of **$[RuL_3]^{3+}$**.

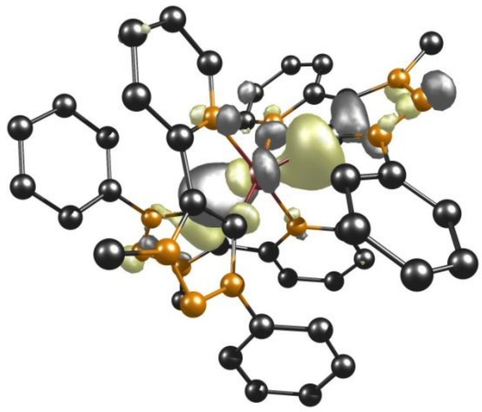

***β*-HOMO-11**

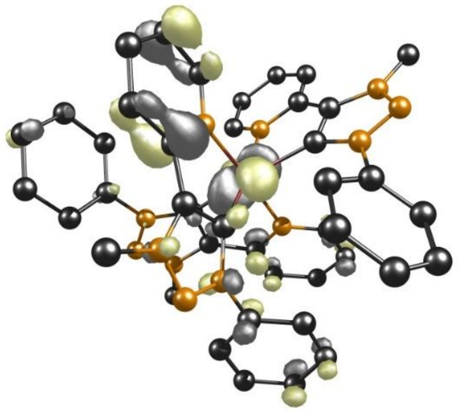

***β*-HOMO-10**

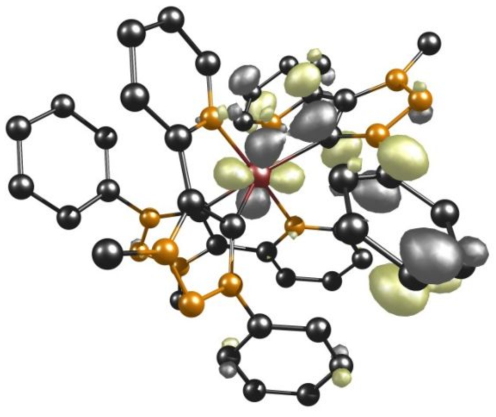

***β*-HOMO-9**

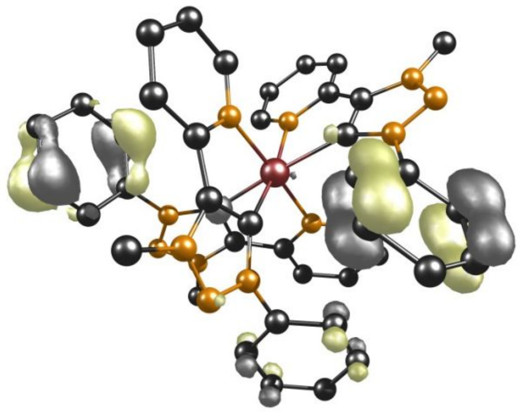

***β*-HOMO-8**

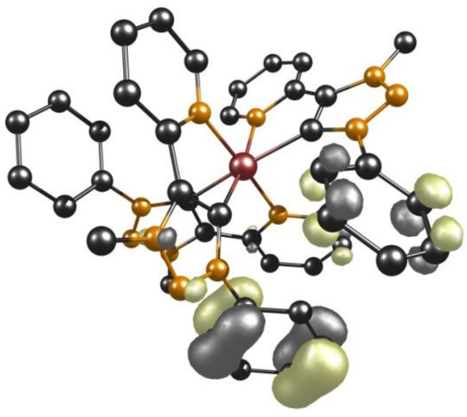

***β*-HOMO-5**

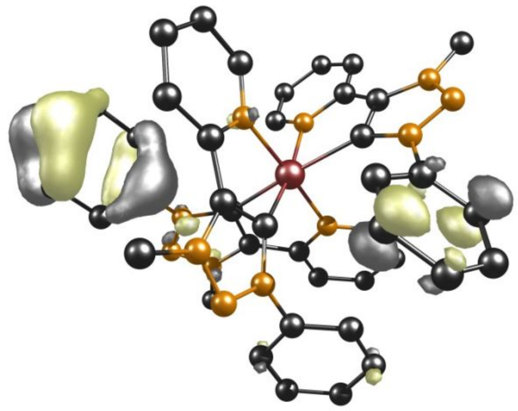

***β*-HOMO-4**

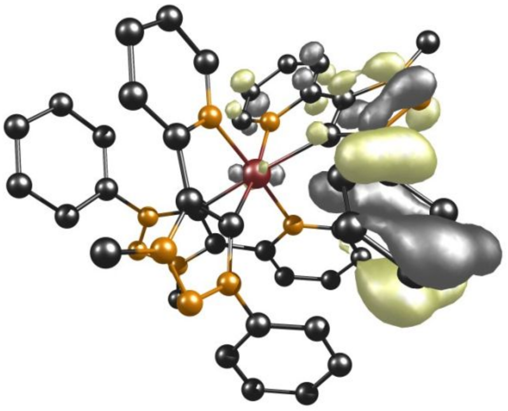

***β*-HOMO-3**

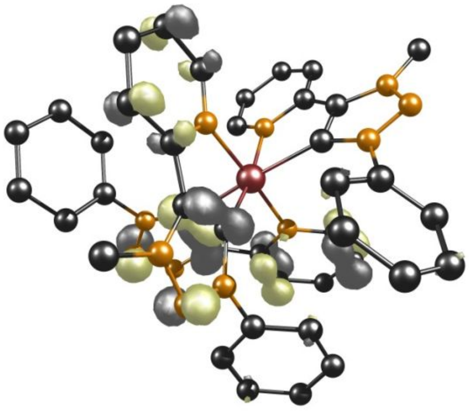

***β*-HOMO-2**

Figure S53. Involved TD-DFT orbitals of complex **[RuL$_3$]**$^{3+}$.

Table S24. Selected MO energies of **$[RuL_3]^{2+}$** and **$[RuL_3]^{3+}$**.

$[RuL_3]^{2+}$		$[RuL_3]^{3+}$	
**MO**	**Energy / eV**	**MO**	**Energy / eV**
**HOMO-2**	– 6.1287	***β*-HOMO-11**	– 9.1442
**HOMO-1**	– 6.0243	***β*-HOMO-10**	– 8.6291
**LUMO+1**	– 2.1231	***β*-HOMO-9**	– 8.3284
**LUMO+2**	– 2.0728	***β*-HOMO-8**	– 8.1778
**LUMO+3**	– 1.6921	***β*-HOMO-5**	– 8.0808
		***β*-HOMO-4**	– 8.0765
		***β*-HOMO-3**	– 7.9272
		***β*-HOMO-2**	– 7.8010

Table S25. XYZ coordinates of optimized **$[RuL_3]^{2+}$**.

	x	y	z
Ru	4.198085606185	7.252233401860	5.683479938083
C	3.274812917558	6.264059237148	7.224373057722
N	1.430041092940	5.786106241924	8.368805906055
N	5.456652116228	8.670954931964	3.065855604971
C	1.883725092738	6.368899496301	7.233189992258
C	0.074524073741	5.657552471004	8.862742139648
H	0.115097490669	5.134125679007	9.813279176554
H	-0.518200948793	5.087304680955	8.149580934193
H	-0.351222262216	6.650381601943	9.001011824179
N	4.176858488165	9.074073884145	6.743282093220
C	1.207849936111	7.082539524645	6.173326902464
N	8.373594959687	6.662909348183	5.817526855902
C	-0.158792175240	7.265473505212	6.011209431348
H	-0.858477845926	6.859767640177	6.727297485702
N	7.298188238723	7.307709446995	6.266803164541
C	4.762857575216	5.275727380042	8.953158081420
C	1.646543856740	8.265763197090	4.237239824575
H	2.396085659190	8.661296572364	3.565257589570
C	0.300364153463	8.475454194427	4.004892406554
H	-0.012355270036	9.032428511577	3.131868083772
C	-0.617897208165	7.966062190950	4.907521004317
H	-1.680181043820	8.113469550463	4.755029998078
N	3.498235782285	5.599709220666	8.392677694377
N	5.537862347728	10.624327845936	3.767400584799
N	4.314286704464	5.498216524163	4.554907208163
N	5.873832209054	9.891320389487	2.745409575493
N	2.104529721759	7.583096352889	5.286060046582
N	2.404673591929	5.308020390297	9.084386495595
N	7.891300879601	5.705742442212	5.078349690437
C	5.705634854776	4.650871303034	8.154737667024
H	5.447129694704	4.371108983295	7.143019557426
C	6.959432155572	4.384349800016	8.680915174430

H	7.705139626289	3.893082619021	8.067444240933
C	7.253709795052	4.730759174377	9.993761138134
H	8.233712945588	4.515623727073	10.403009753273
C	6.293142395885	5.346268866325	10.785171290691
H	6.524108630465	5.626018326305	11.806190104997
C	5.036965553529	5.625000204715	10.266301040943
H	4.282039205485	6.120141239505	10.864539760751
C	7.090015947675	8.489530551559	8.377830388123
H	6.646189982730	7.603404520561	8.816298369773
C	7.494151350787	8.477831264022	7.054459335012
C	3.261595811187	4.874707974857	4.021264104058
H	2.294607904810	5.317305456114	4.216185478894
C	3.373501398469	3.733497823668	3.250361974263
H	2.480517912327	3.278735026845	2.841663696211
C	4.632003877315	3.207613496717	3.005942586037
H	4.755813401463	2.325439550217	2.390381588561
C	5.734319583791	3.830340463444	3.562990951114
H	6.726873341193	3.437037800034	3.394379137202
C	5.549487892475	4.962312595935	4.343719384003
C	8.809327554826	4.815935474402	4.399792738222
H	8.623115600873	3.790848500108	4.717066946466
H	9.818096024263	5.113783093129	4.670166626781
H	8.666610279965	4.911166050493	3.323425357343
C	6.541199004225	5.728896137827	5.043943645601
C	6.113511459761	6.799124441874	5.834576319268
C	4.615105828135	6.931933919101	1.612376009318
H	3.609070840526	7.228660891097	1.880789231003
C	4.843595640439	5.887425793599	0.731876830279
H	4.003016330056	5.352016144021	0.308114403172
C	6.142395301474	5.525151401079	0.402943608877
H	6.316164160473	4.702286453096	-0.280046379081
C	7.219830794939	6.207776813715	0.953560247735
H	8.232979459437	5.915882665091	0.702037640705
C	7.002344232925	7.258012331798	1.834352491973
H	7.829762827726	7.797724807213	2.280191307800
C	5.698730733201	7.605959545597	2.149833334414
C	3.812958430555	9.202588484049	8.020261872859
H	3.607094604201	8.285688046775	8.551987952052
C	3.694738373415	10.422718040306	8.657707206945
H	3.398588703693	10.450464721719	9.698103463090
C	3.945836026795	11.579778061523	7.941762172299
H	3.837400905328	12.553979703021	8.401864302396
C	4.345220969275	11.469219029897	6.621927104520
H	4.548959746243	12.354336765866	6.037064072087
C	4.470113861443	10.210122006912	6.054843074641
C	5.873727706688	12.033499420729	3.760235219329
H	6.440826416618	12.269041320288	4.660028813375
H	4.960492329220	12.625550135802	3.726803190850
H	6.478991386319	12.226240903494	2.879750006866
C	4.911760956053	9.896311284449	4.719714696886
C	4.851570230974	8.578634985041	4.278521027491
C	7.266164006894	9.649020676050	9.118494852632
H	6.952417051315	9.677234150471	10.155179958425
C	7.838824193410	10.771647592995	8.535937132479
H	7.972785527490	11.673577788563	9.121479032172
C	8.238895095630	10.742350790729	7.206517446504
H	8.682030307543	11.617837683524	6.746888913273
C	8.067157212804	9.589233642785	6.457868156298
H	8.367655883300	9.548580680105	5.417436460229

Table S26. XYZ coordinates of optimized **[RuL$_3$]$^{3+}$**.

	x	y	z
Ru	4.176640193505	7.279207192563	5.702485566792
C	3.245491584091	6.231280959093	7.224925022962
N	1.409513795323	5.681168815376	8.318658694791
N	5.511597066357	8.677385384079	3.094717297882
C	1.862504759520	6.319239686993	7.211841655031
C	0.048939045245	5.503770788307	8.787781297259
H	0.090448185989	4.951073954406	9.721293546393
H	-0.515100332123	4.941999202065	8.045469748889
H	-0.399724614123	6.482258245912	8.950924928190
N	4.135725095246	9.098379123100	6.747444688072
C	1.190889855993	7.057860709020	6.165587571357
N	8.362481612915	6.709825872008	5.783408001083
C	-0.172892126004	7.218233583066	5.984243158451
H	-0.877326313249	6.763171989448	6.664823957174
N	7.301639837764	7.368774862008	6.221472532868
C	4.757579884030	5.224043518204	8.914029725762
C	1.643382179777	8.351072453939	4.294277733811
H	2.393676634244	8.798457249472	3.656607857748
C	0.297617469150	8.539376031588	4.047713037070
H	-0.011595590542	9.132320627081	3.198013960400
C	-0.622989555988	7.961807281207	4.904315019323
H	-1.685053700404	8.090247658754	4.735525772163
N	3.481496439262	5.531424627075	8.360919725410
N	5.627874830458	10.625047818620	3.811734211049
N	4.290751698406	5.497501105559	4.612242520235
N	5.956284518034	9.884873141785	2.793790709455
N	2.088305223815	7.620285532729	5.316299840082
N	2.389847398347	5.199233591936	9.023276221846
N	7.881164730864	5.732442079684	5.072116107117
C	5.689139362563	4.586479143421	8.113352212302
H	5.411153323858	4.262230087469	7.119699933348
C	6.954631519247	4.346860015630	8.624081106701
H	7.693386313161	3.841006368601	8.014248699650

C	7.265757382372	4.733401510508	9.921699853641
H	8.254273639927	4.537923158470	10.319723277767
C	6.313700842171	5.360857144992	10.714097194342
H	6.559987119896	5.669435725913	11.722884635377
C	5.045448985989	5.613630864337	10.211753179231
H	4.294998535601	6.116897473411	10.808862983673
C	7.080472907562	8.580220970492	8.314523894592
H	6.647493100436	7.696758593024	8.769961825412
C	7.494512463344	8.553209442266	6.994593584511
C	3.224798872565	4.871862753640	4.108747353662
H	2.258586236112	5.306548218343	4.321285813448
C	3.331960039676	3.729469548350	3.341068687752
H	2.435021524229	3.267279945714	2.951361457407
C	4.589006214297	3.210515425504	3.079093617682
H	4.704626180601	2.323574017135	2.469139943971
C	5.702659629709	3.839072926336	3.608322865691
H	6.692429279913	3.446242495394	3.425092458252
C	5.526533638002	4.974208900005	4.380860708220
C	8.794588472080	4.819331590293	4.413800415767
H	8.597009548692	3.805299253181	4.757279569624
H	9.803940020594	5.117425960593	4.680727441190
H	8.652750043017	4.893074530213	3.335922086685
C	6.528335477680	5.752083947871	5.054091559389
C	6.125096880084	6.834163694470	5.820648401939
C	4.628339993669	6.940198433457	1.665021761678
H	3.628153229312	7.264568216416	1.924982762909
C	4.834407023857	5.877551489519	0.801003039788
H	3.982589067699	5.352420469780	0.387294859249
C	6.125666159394	5.488702971872	0.471571878002
H	6.281453184205	4.652269760305	-0.198843322043
C	7.218640896028	6.163330626400	1.001421174144
H	8.224780360482	5.852744963687	0.745356021797
C	7.026322851452	7.231841336457	1.865582670452
H	7.865770057280	7.767057959609	2.293906481036
C	5.729569959541	7.601259132068	2.182724721764
C	3.723776986325	9.215620132452	8.011372132480
H	3.446014312214	8.303445839298	8.517529465720
C	3.649952444074	10.429957276293	8.662599738515
H	3.318747943280	10.459282725509	9.691765012676
C	3.987689622091	11.580266251494	7.972966413727
H	3.916550572213	12.551188918295	8.446443319838
C	4.423801259557	11.474525443618	6.663950084798
H	4.689749383806	12.358734265810	6.103926373264
C	4.504921414846	10.223278694656	6.079011258848
C	5.993235066275	12.028592735145	3.815007822985
H	6.567752790163	12.243447416513	4.715252425991
H	5.089445221207	12.634689902148	3.787759837187
H	6.599810863551	12.213452524829	2.934003368581
C	4.964308992841	9.908012959506	4.749728014421
C	4.881231026253	8.606605584922	4.289854446255
C	7.247946238832	9.752593650940	9.036553003193
H	6.931256115449	9.795505466939	10.071522503555
C	7.819989576023	10.866978437286	8.437607606648
H	7.946499234732	11.778672508585	9.009000622096
C	8.232387285433	10.817574263854	7.112573503353
H	8.679682519400	11.685792771099	6.644003815605
C	8.071672408443	9.652190514978	6.380609050320
H	8.388453580792	9.592766565610	5.345939261280

7.40 Latimer Diagram

Table S27. Latimer-Diagram with relevant potentials for photoexcitation. All Potentials with FcH/FcH$^+$ were converted vs SCE = 0.38 V in MeCN/0.1M NBu_4PF_6 at room temperature.[19]

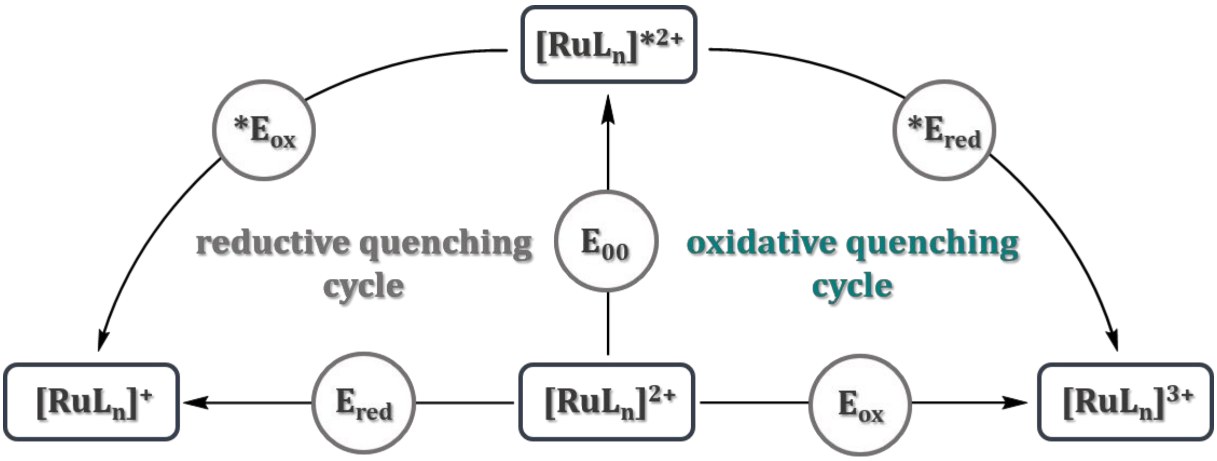

	E_{red}/ V	E_{ox}/ V	$^*E_{red}$/ V	$^*E_{ox}$/ V	E_{00}/ eV
**$[RuL_1]^{2+}$**	−1.43	1.09	−1.12	0.78	2.21
**$[RuL_2]^{2+}$**	−1.51	0.90	−1.17	0.56	2.07
**$[RuL_3]^{2+}$**	−1.76	0.76	−1.29	0.29	2.05
**$[Ru(bpy)_3]^{2+}$**	−1.34[20]	1.27[20]	−0.83[20]	0.76[20]	2.10[20]

[19] N. G. Connelly, W. E. Geiger, *Chem. Rev.* **1996**, *96*, 877−910.

[20] A. Juris, V. Balzani, F. Barigelletti, S. Campagna, P. Belser, A. von Zelewsky, *Coord. Chem. Rev.* **1988**, *84*, 85−277.

8. Photophysical Measurments

8.10 Photophysic of $[RuL_1](PF_6)_2$

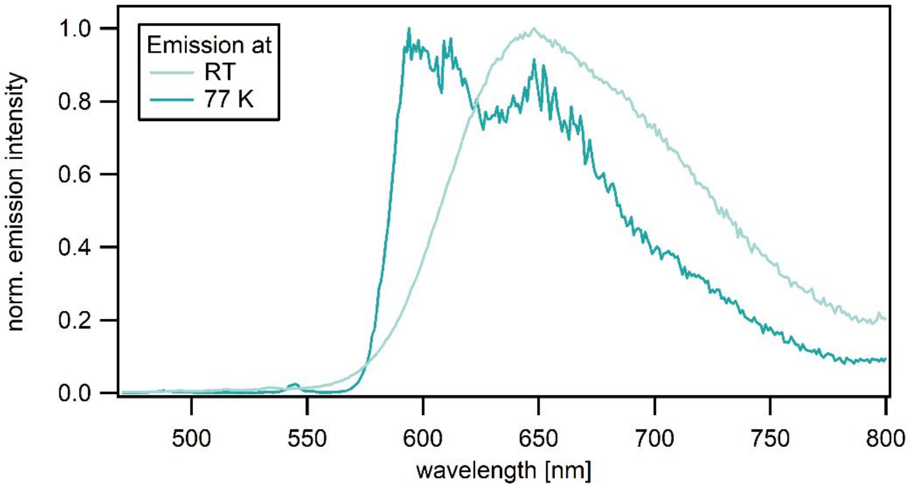

Figure S54. Emission at RT (light blue, in MeCN) and 77 K (dark blue, in 2-methyl-THF) of **$[RuL_1](PF_6)_2$** with an excitation wavelength of 460 nm.

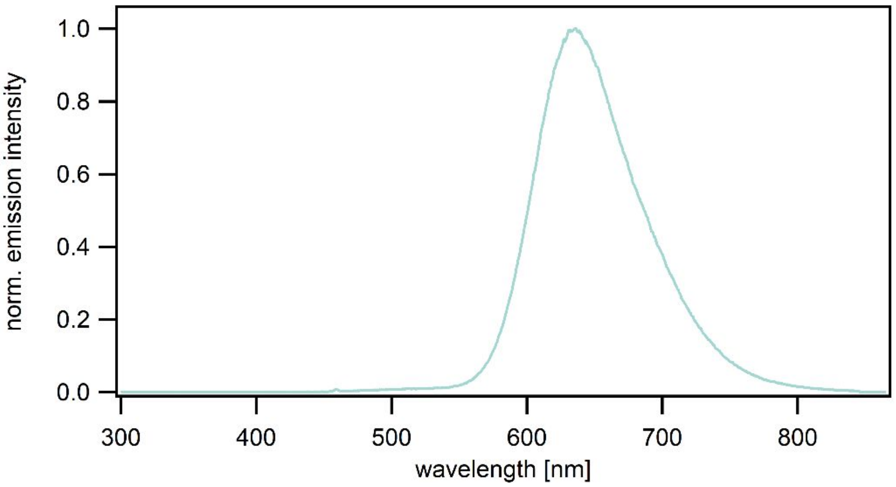

Figure S55. Spectral emission of **[RuL$_1$](PF$_6$)$_2$** (in MeCN) after 0 ns delay with an excitation wavelength of 460 nm.

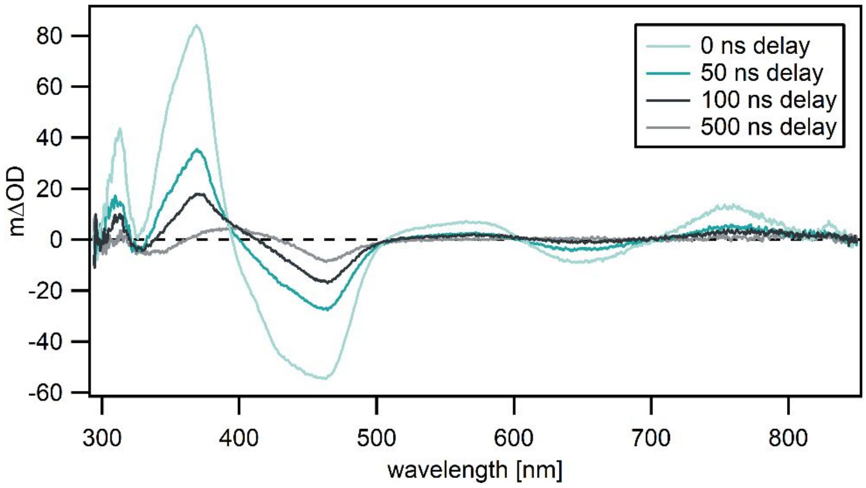

Figure S56. Transient absorption spectrum of **[RuL$_1$](PF$_6$)$_2$** (in MeCN) after 0 ns (light blue), 50 ns (dark blue), 100 ns (black) and 500 ns (grey) delay with an excitation wavelength of 460 nm.

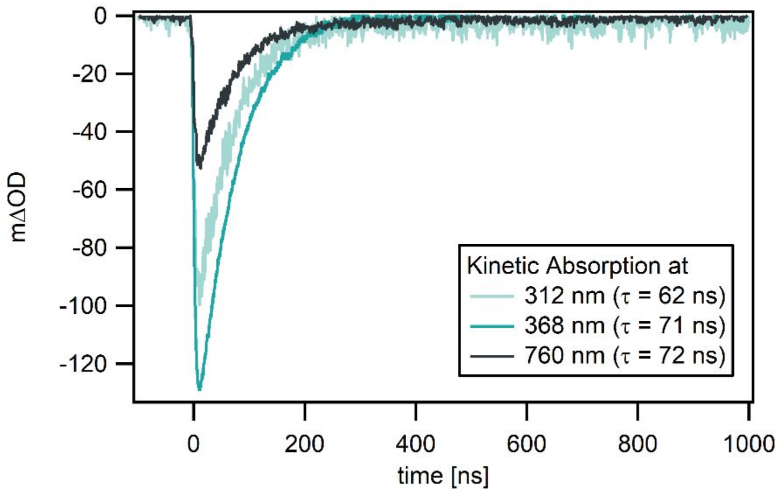

Figure S57. Kinetic absorption at 312 nm (τ = 62 ns) (light blue), 368 nm (τ = 71 ns) (dark blue) and 760 nm (τ = 72 ns) (black) of **$[RuL_1](PF_6)_2$** (in MeCN) with an excitation wavelength of 460 nm.

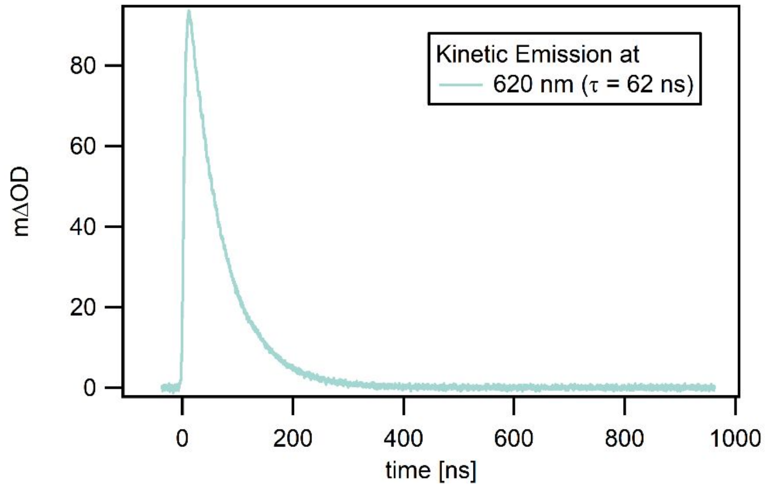

Figure S58. Kinetic emission of **$[RuL_1](PF_6)_2$** (in MeCN) at 620 nm (τ = 62 ns) with an excitation wavelength of 460 nm.

8.20 Photophysic of $[RuL_2](PF_6)_2$

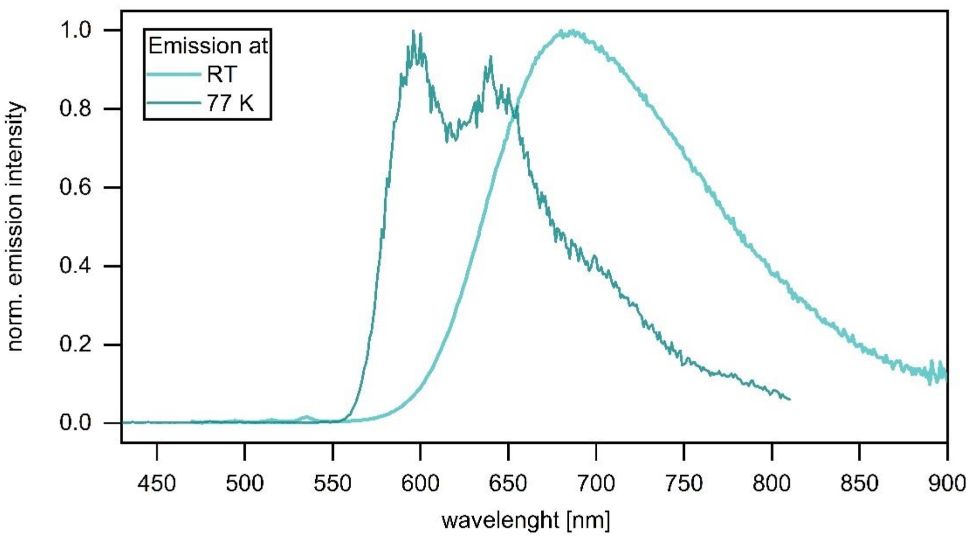

Figure S59. Emission at RT (light blue, in MeCN) with an excitation wavelength of 460 nm and 77 K (dark blue, in 2-methyl-THF) of **[RuL$_2$](PF$_6$)$_2$** with an excitation wavelength of 420 nm.

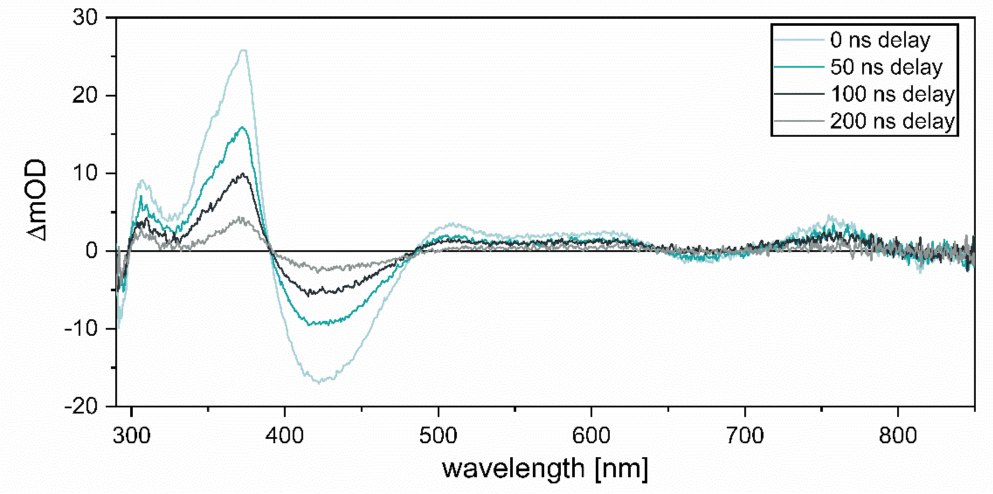

Figure S60. Transient absorption spectrum of **[RuL$_2$](PF$_6$)$_2$** (in MeCN) after 0 ns (light blue), 50 ns (dark blue), 100 ns (black) and 200 ns (grey) delay with an excitation wavelength of 430 nm.

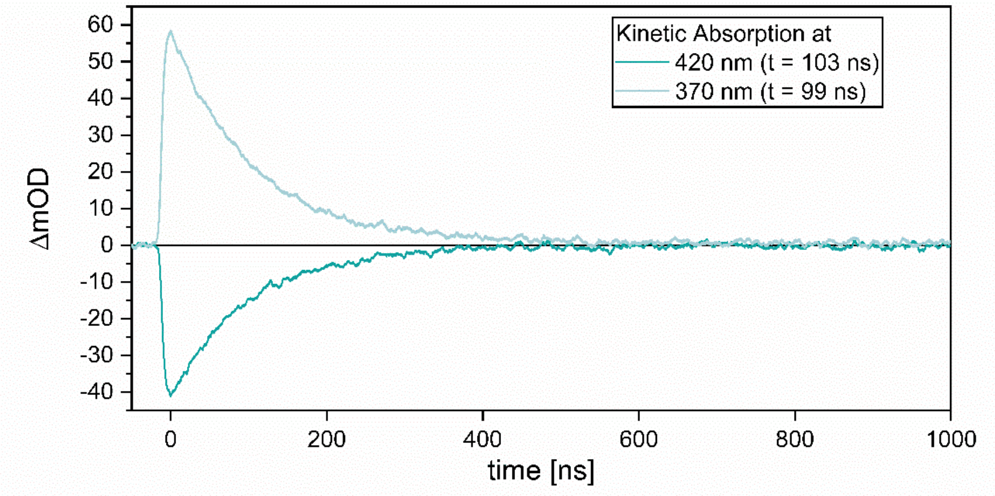

Figure S61. Kinetic absorption at 370 nm (τ = 99 ns) (light blue), 420 nm (τ = 103 ns) (dark blue) of **[RuL$_2$](PF$_6$)$_2$** (in MeCN) with an excitation wavelength of 430 nm.

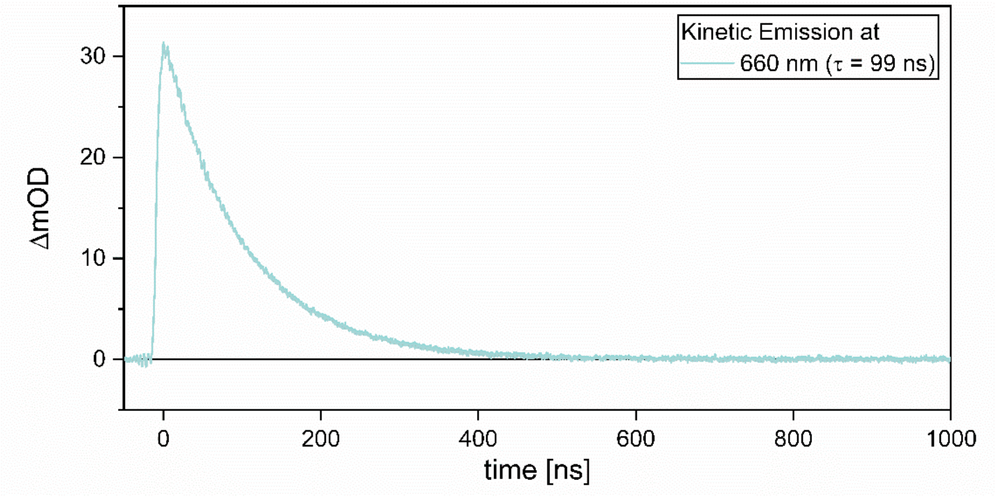

Figure S62. Kinetic emission of **[RuL$_2$](PF$_6$)$_2$** (in MeCN) at 660 nm (τ = 99 ns) with an excitation wavelength of 430 nm.

8.30 Photophysic of [RuL$_3$](PF$_6$)$_2$

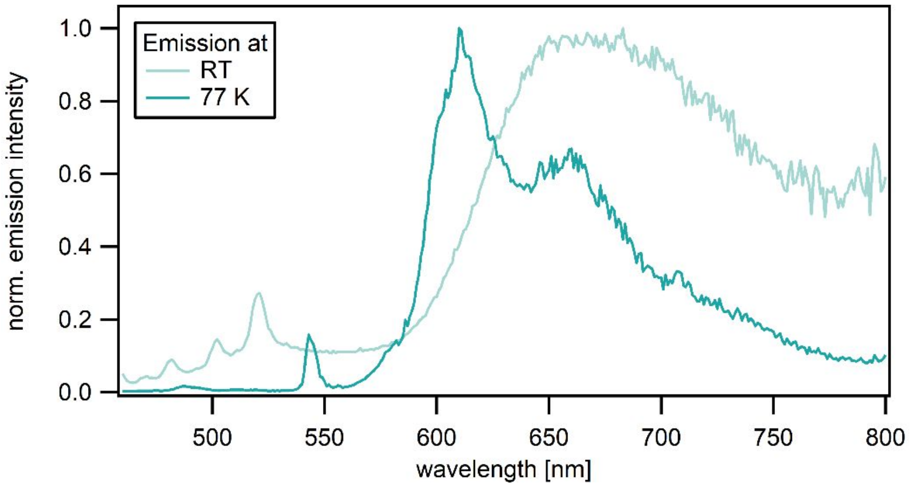

Figure S63. Emission at RT (light blue, in MeCN) and 77 K (dark blue, in 2-methyl-THF) of **[RuL$_3$](PF$_6$)$_2$** with an excitation wavelength of 435 nm and 450 nm, respectively.

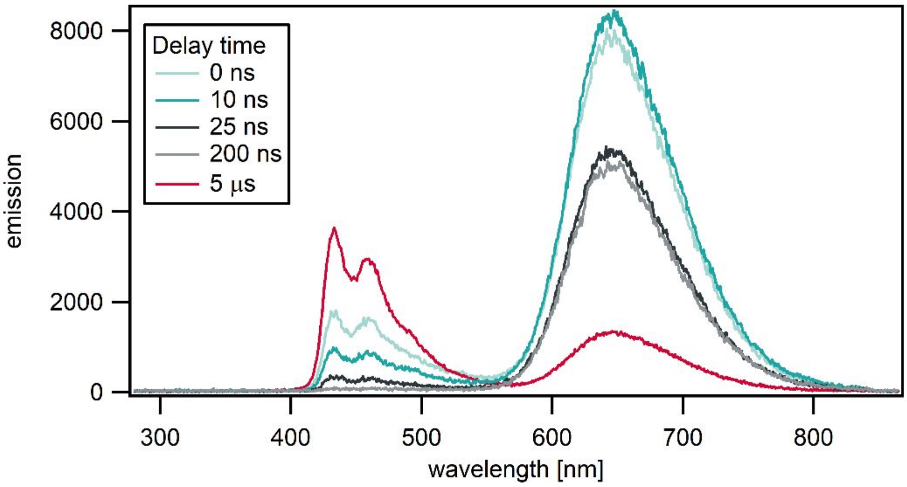

Figure S64. Spectral emission of **[RuL$_3$](PF$_6$)$_2$** (in MeCN) after different time delays.

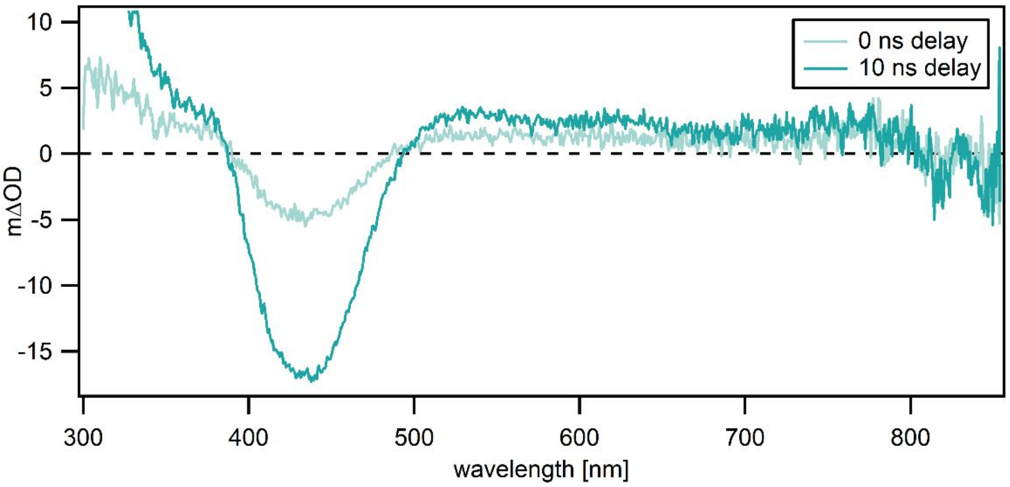

Figure S65. Transient absorption spectrum of **[RuL$_3$](PF$_6$)$_2$** (in MeCN) after 0 ns (light blue) and 10 ns (dark blue) delay.

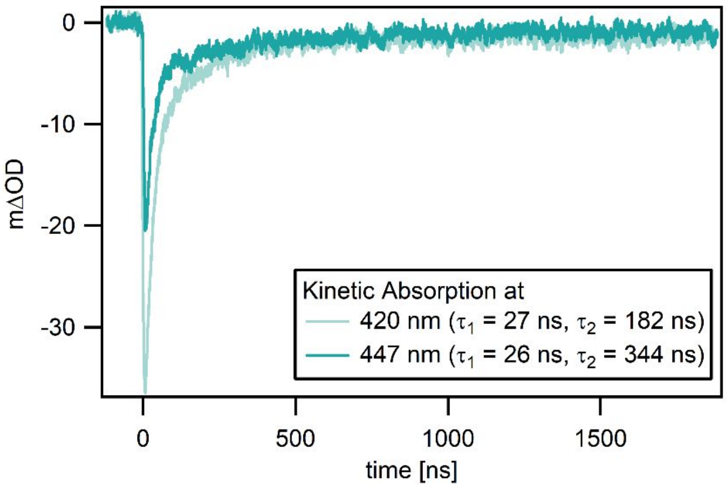

Figure S66. Kinetic absorption at 420 nm (τ_1 = 27 ns, τ_2 = 182 ns) and 447 nm (τ_1 = 26 ns, τ_2 = 344 ns) of **[RuL$_3$](PF$_6$)$_2$** (in MeCN) with an excitation wavelength of 435 nm.

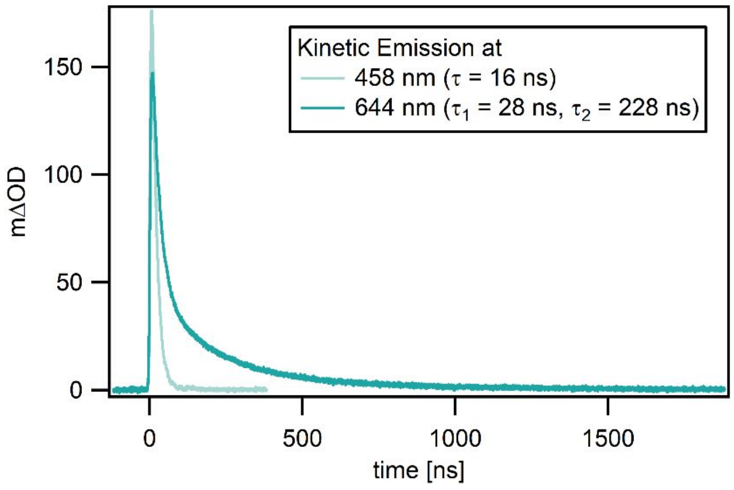

Figure S67. Kinetic emission of **$[RuL_3](PF_6)_2$** (in MeCN) at 458 nm (τ = 16 ns) and 644 nm (τ_1 = 28 ns, τ_2 = 228 ns) with an excitation wavelength of 435 nm.

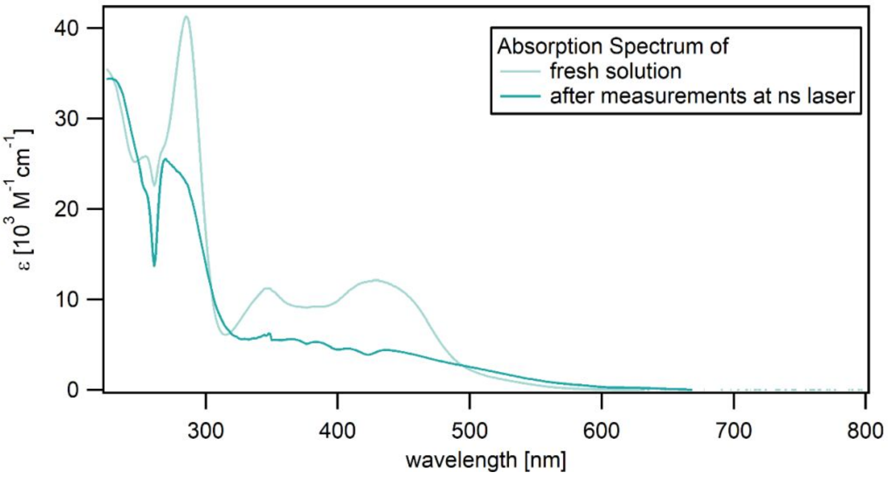

Figure S68. Absorption spectra of **$[RuL_3](PF_6)_2$** (in MeCN) before (light blue) and after (dark blue) irradiation with ns-Laser.
